# Comparative Effectiveness of Pharmacological Interventions for Covid-19: A Systematic Review and Network Meta-Analysis

**DOI:** 10.3389/fphar.2021.649472

**Published:** 2021-05-03

**Authors:** Franco De Crescenzo, Laura Amato, Fabio Cruciani, Luke P Moynihan, Gian Loreto D’Alò, Simona Vecchi, Rosella Saulle, Zuzana Mitrova, Valeria Di Franco, Antonio Addis, Marina Davoli

**Affiliations:** ^1^ Department of Psychiatry, University of Oxford, Oxford, United Kingdom; ^2^ Department of Epidemiology of the Regional Health Service Lazio, Rome, Italy; ^3^ Paediatric University Hospital-Department (DPUO), Bambino Gesù Children’s Hospital, Rome, Italy; ^4^ Department of Acute Medicine, University Hospital Southampton NHS Foundation Trust, Southampton, United Kingdom; ^5^ Department of Anaesthesiology and Intensive Care Medicine, Fondazione Policlinico Universitario A. Gemelli IRCCS, Rome, Italy

**Keywords:** COVID-19, systematic (literature) review, network meta analysis, adults (MeSH), pharmacologic (drug) therapy

## Abstract

**Background:** Several pharmacological interventions are now under investigation for the treatment of Covid-19, and the evidence is evolving rapidly. Our aim is to assess the comparative efficacy and safety of these drugs.

**Methods and Findings:** We performed a systematic review and network meta-analysis searching Medline, Pubmed, Embase, Cochrane Covid-19 register, international trial registers, medRxiv, bioRxiv, and arXiv up to December 10, 2020. We included all randomised controlled trials (RCTs) comparing any pharmacological intervention for Covid-19 against any drugs, placebo or standard care (SC). Data extracted from published reports were assessed for risk of bias in accordance with the Cochrane tool, and using the GRADE framework. Primary outcomes were all-cause mortality, adverse events (AEs) and serious adverse events (SAEs). We estimated summary risk ratio (RR) using pairwise and network meta-analysis with random effects (Prospero, number CRD42020176914). We performed a systematic review and network meta-analysis searching Medline, Pubmed, Embase, Cochrane Covid-19 register, international trial registers, medRxiv, bioRxiv, and arXiv up to December 10, 2020. We included all randomised controlled trials (RCTs) comparing any pharmacological intervention for Covid-19 against any drugs, placebo or standard care (SC). Data extracted from published reports were assessed for risk of bias in accordance with the Cochrane tool, and using the GRADE framework. Primary outcomes were all-cause mortality, adverse events (AEs) and serious adverse events (SAEs). We estimated summary risk ratio (RR) using pairwise and network meta-analysis with random effects (Prospero, number CRD42020176914). We included 96 RCTs, comprising of 34,501 patients. The network meta-analysis showed in terms of all-cause mortality, when compared to SC or placebo, only corticosteroids significantly reduced the mortality rate (RR 0.90, 95%CI 0.83, 0.97; moderate certainty of evidence). Corticosteroids significantly reduced the mortality rate also when compared to hydroxychloroquine (RR 0.83, 95%CI 0.74, 0.94; moderate certainty of evidence). Remdesivir proved to be better in terms of SAEs when compared to SC or placebo (RR 0.75, 95%CI 0.63, 0.89; high certainty of evidence) and plasma (RR 0.57, 95%CI 0.34, 0.94; high certainty of evidence). The combination of lopinavir and ritonavir proved to reduce SAEs when compared to plasma (RR 0.49, 95%CI 0.25, 0.95; high certainty of evidence). Most of the RCTs were at unclear risk of bias (42 of 96), one third were at high risk of bias (34 of 96) and 20 were at low risk of bias. Certainty of evidence ranged from high to very low.

**Conclusion:** At present, corticosteroids reduced all-cause mortality in patients with Covid-19, with a moderate certainty of evidence. Remdesivir appeared to be a safer option than SC or placebo, while plasma was associated with safety concerns. These preliminary evidence-based observations should guide clinical practice until more data are made public.

## Introduction

The emergence of the novel coronavirus SARS-CoV-2 in December 2019 has posed both the scientific community and wider society challenges of an unprecedented scale and nature. It is highly transmissible resulting in a rapid outbreak globally and was declared a pandemic by the world health organisation (WHO) on March 11th.

Coronavirus disease (Covid-19) can be asymptomatic or can manifest with a wide range of symptoms ranging from mild respiratory ailments to a fatal acute respiratory syndrome and multi-organ failure. The mortality rate is associated with age, gender and comorbidity (Horby and Lim, 2020). Until recently there has been no compelling evidence that any pharmacological treatment of Covid-19 improves outcomes, meaning that supportive care has been the mainstay of management. Dexamethasone has been shown in a large multi-arm trial to be superior to standard care for all-cause mortality (Karagiannidis et al., 2020).

Various other pharmacological agents have been touted as potential treatments for Covid-19, with a preponderance for established antiviral drugs licensed in the treatment of other infections (Sanders et al., 2020). None of these has yet come to the forefront or obtained a strong evidence base as an effective and safe treatment for Covid-19. Since the outbreak of the SARS-CoV-2 epidemic anecdotal evidence, non-peer reviewed articles and strong claims from small clinical trials have exposed clinicians and patients to the risks associated with the use of off-label medicines with very low level evidence (Fauci et al., 2020; Kalil, 2020).

This study comes at a pivotal time whereby a substantial amount of research has been simultaneously carried out in a coordinated global effort and over a short timescale. Prospectively designed network meta-analyses based on existing and future randomised trials can generate high quality comparative evidence, which can be used to assess drugs used against Covid-19 (Cipriani et al., 2020; Naci et al., 2020). Therefore, in this study, we aimed to do a systematic review and network meta-analysis of randomised controlled trials to inform clinical practice and regulatory agencies by comparing different pharmacological interventions versus standard care, placebo or any other intervention for the treatment of Covid-19.

## Materials and Methods

This study is part of a living review of pharmacological agents for the treatment of Covid-19 conducted by the Department of Epidemiology of the Regional Health Service Lazio, Italy, to inform national regulatory agencies and clinicians, available at https://www.deplazio.net/farmacicovid. This living review is also part of the rolling collaborative reviews published on a monthly basis with the European Network of Health Technology Assessment (EUnetHTA) and available at https://eunethta.eu/covid-19-treatment/.

This living review was conducted following a pre-established protocol registered on PROSPERO (CRD42020176914). The amended protocol with a full search strategy is detailed in Supplementary Appendix S1 and the review is hereby reported according to the Preferred Reporting Items for Systematic Reviews and Meta-analyses (PRISMA) guidelines, detailed in Supplementary Appendix S2 (Hutton et al., 2015). In order to have a full evaluation of the safety, the evaluation of adverse events and serious adverse events were included as primary outcomes in the amended version of the protocol.

### Search Strategy and Selection Criteria

We searched Medline, PubMed, and embase from December 2019 to December, 10 2020. We searched medRxiv.org (https://www.medrxiv.org/), bioRxiv.org (https://www.bioRxiv.org/), and arXiv.org (https://www.arXiv.org/) for preprints of preliminary reports of randomised trials. We also searched the Cochrane Covid-19 Study Register (https://covid-19.cochrane.org/), ClinicalTrials.gov (www.clinicaltrials.gov) and World Health Organization (WHO) International Clinical Trials Registry Platform (ICTRP) (www.who.int/ictrp/en/). Additional sources included journal alerts, contact with researchers, websites such as Imperial College, London School of Hygiene and Tropical Medicine, and Eurosurveillance. We applied no restriction on language of publication.

We included parallel randomised controlled trials (RCTs) comparing any pharmacological intervention against another pharmacological intervention or placebo or standard care (SC), for the treatment of individuals with Covid-19. We included individuals >18 years of age affected by Covid-19 as defined by the authors of the studies. There were no limits in terms of gender or ethnicity or severity of disease. We included pharmacological interventions without restrictions on dosage, regimen, dosing interval, route of administration, or intervention duration. We included standard care as defined by study authors. All studies had standard care underlying the control arms, and we grouped together standard care and placebo as a common comparator. We did not include quasi-randomized controlled trials, cross-over trials, or pilot studies with a single arm.

We excluded studies comparing two dosages of the same pharmacological agent. We did not exclude studies on individuals with a comorbid disorder.

### Data Extraction

Four authors (FC, GLD, SV, ZM) independently screened the references retrieved by the search, selected the studies, and extracted the data, using a predefined data-extraction sheet, including the following data:

Methods: first author or acronym, year of publication, study design.
Participants: diagnosis, sample size, mean age, gender distribution, severity of illness, setting.
Interventions: number of patients allocated to each arm, drug name, dose, duration of the interventions and follow-up.
Outcomes: all-cause mortality, adverse events and serious adverse events.
Additional outcomes: Patients with SARS-CoV-2 nasal or pharyngeal swab RT-PCR clearance, time to nasal or pharyngeal swab RT-PCR clearance, number of patients with improvement of pulmonary disease (CT imaging), number of patients experiencing disease progression, number of patients discharged from the hospital, and length of hospital stay.
Notes: Country, funding source.

The same reviewers discussed any uncertainty regarding study eligibility and data extraction until consensus was reached; conflicts of opinion were resolved with other members of the review team (FDC, LA, RS). Two authors (FC, RS) independently assessed the risk of bias of the included studies with the Cochrane tool (Higgins and Green, 2011). Three authors (FC, FDC, GLD) used the Grading of Recommendations Assessment, Development and Evaluation (GRADE) approach (Salanti et al., 2014), through the Confidence in Network Meta-Analysis Software (University of Bern Institute of Social and Preventive Medicine, 2017), to evaluate the strength of evidence for results at the end of treatment from the network meta-analysis. We did rate the double blinded studies using placebo as having lower risk of bias, which is reflected on the GRADE evaluation (see Supplementary Appendix S1). We considered an OR of 0.80 for mortality and an OR of 1.25 for adverse events and serious adverse events as clinically meaningful, following Cipriani et al. (2018). Using the GRADE approach, we assessed each network estimate according to the following criteria: study limitation, indirectness, inconsistency, imprecision, publication bias. We derived the overall judgment of the certainty of evidence considering the domains altogether and downgraded the evidence by one if a domain was rated as “some concerns” and by two if a domain was rated as “major concerns”. Finally, we assigned to each comparison an overall qualitative judgment based on four levels of certainty of evidence: high, moderate, low, very low.

### Outcomes

We considered as primary outcomes all-cause mortality at the longest follow up and safety (number of patients experiencing any adverse event and serious adverse event) at the end of treatment. Secondary outcomes were measured at study endpoint and included number of patients with SARS-CoV-2 nasal or pharyngeal swab RT-PCR clearance, time to nasal or pharyngeal swab RT-PCR clearance, number of patients with improvement of pulmonary disease (CT imaging), number of patients experiencing disease progression, number of patients discharged from the hospital, and length of hospital stay.

### Dealing With Missing Data

When dichotomous outcome data were missing, they were managed according to the intention-to-treat (ITT) principle, and we assumed that patients who dropped out after randomisation had a negative outcome. Missing continuous outcome data were analysed using the last observation carried forward to the final assessment (LOCF). Where LOCF data were not reported by the trial authors, continuous outcomes data were analysed on an endpoint basis, including only participants with a final assessment. When *p* values, t-values, CIs or standard errors were reported in articles, we calculated SDs from their values as in Higgins et al. (2011).

### Data Analysis

First, we performed pairwise meta-analyses using a random-effects model to estimate pooled risk ratios (RRs) for dichotomous outcomes. We narratively reported hazard ratios (HRs) when RRs were not available. We reported standardised mean differences (SMDs) for continuous outcomes with their 95% confidence intervals (CIs) using The Cochrane Collaboration, 2014. We assessed statistical heterogeneity in each pairwise comparison with τ^2^, I^2^ statistic, and *p* value (Higgins and Green, 2011).

We incorporated indirect comparisons with direct comparisons for primary outcomes using random-effects network meta-analyses within a frequentist framework using STATA 16 (network package), and results are presented with the network graphs package (Chaimani et al., 2013). We report the results of network meta-analyses in league tables with effect sizes (RR) and their 95% CIs. While in the pairwise meta-analyses we included all the treatments, we included in our network meta-analysis only those treatments with >100 individuals randomised as some treatment nodes with few total participants resulted in implausible and imprecise effect estimates, as described in Siemieniuk et al. (2020).

We assessed inconsistency between direct and indirect sources of evidence using local and global approaches. Consistency is an important assumption to check in network meta-analyses because it is the manifestation of transitivity in the data from a network of interventions: consistency exists when treatment effects from direct and indirect evidence are in agreement (subject to the usual variation due to heterogeneity in the direct evidence) (Cipriani et al., 2013). A network-meta-analysis can be misleading if the network is substantially inconsistent. Inconsistency can be present if the trials in the network have very different protocols and their inclusion/exclusion criteria are not comparable or may result as an uneven distribution of the effect modifiers across groups of trials that compare different treatments. We first checked for any erroneous data abstraction. Then, to evaluate the presence of inconsistency locally, we used the loop-specific approach (which identified inconsistent loops of evidence) (Chaimani et al., 2014). This method evaluates the consistency assumption in each closed loop of the network separately as the difference between direct and indirect estimates for a specific comparison in the loop (inconsistency factor). The magnitude of the inconsistency factors and their 95% CIs were used to infer about the presence of inconsistency in each loop. We assumed a common heterogeneity estimate within each loop. Global inconsistency was measured with the between-studies standard deviation (SD) (heterogeneity parameter) by using both a consistency and inconsistency model and by measuring the chi-squared inconsistency, with its *p* value.

We estimated the presence of publication bias and small effect studies by plotting comparison-adjusted funnel plots for the network meta-analyses with a linear regression line (Salanti et al., 2011).

We also estimated the ranking probabilities for all treatments, i.e., their probability of being at each possible rank for each intervention. We report the treatment hierarchy as the surface under the cumulative ranking curve (SUCRA), the probability of being the best and as the mean rank (Salanti et al., 2011).

To determine whether the results were affected by study characteristics, we performed subgroup network meta-analyses for all-cause mortality according to the severity of disease as defined in Jin et al., 2020.

## Results

### Study Characteristics

**FIGURE 1:**
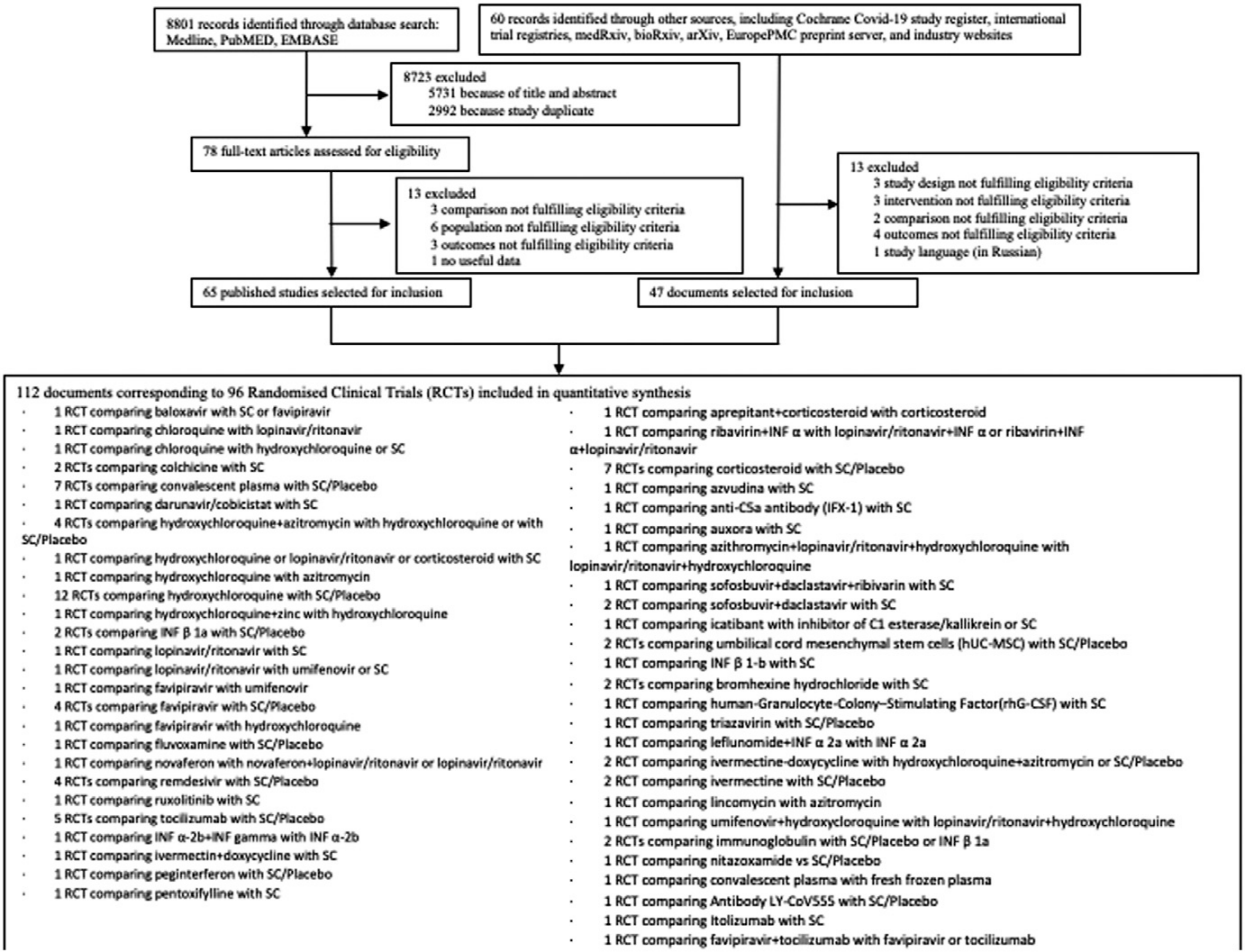
Flow chart.

We identified 8,861 citations from the search and included 112 articles, comprising 96 trials, which randomised 34,501 patients to 59 pharmacological treatments or combination of treatments or SC or placebo (Figure 1). A total of 47 articles were included in the form of preprints or unpublished reports. Table 1 summarizes the characteristics of included studies, and a full list of references for the included studies is available in Supplementary Appendix S3. Further characteristics of the included studies are included in Supplementary Appendix S4.

**TABLE 1:** Characteristics of included randomized controlled trials.

Study, year	Country	Study desig**n**	Setting	Study duration (days)	Longest follow-up (days)	Intervention	N randomised	Mean (SD)/Median (IQR) age (in years)*	% male*	Disease severity (N)
**Abbaspour kasgari, 2020**	Iran	OL	Hospital (single-centre)	NR	14	Sofosbuvir plus daclastavir plus ribivarin	24	Median: 45	46	Moderate (all)
						Standard care	24	Median: 60	29	
**Abd-elsalam, 2020a**	Egypt	NR	Tertiary care units (multicentre)	15	28	Hydroxychloroquine	97	40.4 (18.7)	57.7	Severe (all)
						Standad care	97	41.1 (20.1)	59.8	
**Abd-elsalam, 2020b**	Egypt	NR	Hospital (NR)	6	28	Hydroxychloroquine plus zinc	96	43.48 (14.62)	54.2	Mild (9), moderate (58), severe (18), critical (11)
						Hydroxychloroquine	95	43.64 (13.17)	67.4	Mild (12), moderate (55), severe (20), critical (8)
**Agawal, 2020**	India	OL	Hospitals (multicentre)	2	28	Convalescent plasma	235	Median: 52 (42–60)	75	Moderate (all)
						Standard care	229	Median 52 (41–60)	77	
**AlQathani, 2020**	Bahrain	OL	Hospitals (multicemtre)	2	NR	Convalescent plasma	20	52.6 (14.9)	85	Moderate (all)
						Standard care	20	50.7 (12.5)	75	
**Ansarin, 2020**	Iran	OL	Univerity hospital (single-centre)	14	28	Bromhexine hydrochloride	39	58.4 (13.7)	48.7	NR
						Standard care	39	61.1 (6.1)	61.5	
**Avendaño-solà, 2020**	Spagna	OL	Hospitals (multicentre)	1	29	Convalescent plasma	38	61.3 (16.3)	52.6	Moderate (all)
						Standard care	43	60.3 (15)	55.8	
**Bajpal 2020**	India	OL	Hospital (single-centre)	2	28	Convalescent plasma	15	48.1 (9.1)	78.6	Severe (all)
						Frozen fresh plasma	16	48.3 (10.8)	73.3	
**Beigel, 2020**	United States, Denmark, United Kingdom, Greece, Germany, korea, Mexico, Spain, Japan, Singapore	DB	Hospitals (multicentre)	10	29	Remdesivir	541	58.6 (14.6)	65.1	Severe (476); mild/moderate (62)
						Placebo	521	59.2 (15.4)	63.7	Severe (464); mild/moderate (57)
**Brown, 2020**	United States	OL	Hospitals (multicentre)	5	28	Hydroxychloroquine	42	Median: 51 (42–60)	56	NR
						Azithromycin	43	Median: 58 (43–68)	67	
**Cao B, 2020**	China	OL	Hospital (single-centre)	14	28	Lopinavir/ritonavir	99	58 (50–68)	61.6	Severe (all)
						Standard care	100	58 (48–68)	59.0	
**Cao Y, 2020**	China	SB	Hospital (multicentre)		28	Ruxolitinib	20	63 (51–65	60	Severe (all)
						Standard care	21	64 (59–71)	57.1	
**Cavalcanti AB, 2020**	Brazil	OL	Hospital (multicentre)	7	15	Hydroxychloroquine plus azithromycin^a^	217	49.6 (14.2)	56.7	Mild (NR), moderate (NR)
						Hydroxychloroquine^a^	221	51.3 (14.5)	64.3	
						Standard care^a^	229	49.9 (15.1)	54.2	
**Chen C, 2020**	China	OL	Hospital (multicentre)	7–10^b^	10	Favipiravir	116	NR	50.9	Severe (18); moderate (98)
						Umifenovir	120		42.5	Severe (9); moderate (111)
**Chen CP, 2020**	Taiwan	OL	Hospital (single-centre)	7	14	Hydroxychloroquine	21	33 (12)	52.4	Mild (29), moderate (4)
						Standard care	12	32.8 (8.3)	66.7	
**Chen J, 2020a**	China	OL	Hospital (single-centre)	5	7	Hydroxychloroquine	15	50.5 (3.8)	60.0	Moderate (all)
						Standard care	15	46.7 (3.6)	80.0	
**Chen J, 2020b**	China	OL	Hospital (single-centre)	5	14	Darunavir/Cobicistat	15	51.5 (12.2)	60.0	Moderate (all)
						Standard care	15	42.9 (17.7)	60.0	
**Chen L, 2020**	China	OL		10	28	Chloroquine	25	45.22 (13.66)	38.89	Moderate (all)
						Hydroxychloroquine	28	45.67 (14.37)	44.4	
						Standard care	14	51.33 (15.36)	58.30	
**Chen P, 2020**	United States	DB	Outpatients (single-centre)	11	1 hour	Neutralized antibody LY-CoV555	317	Median: 45 (18–86)	44.7	Mild (all)
						Placebo	150	Median: 46 (18–77)	45.5	
**Chen Z, 2020**	China	OL	Hospital (single-centre)	5	6	Hydroxychloroquine	31	44.10 (16.1)	45.2	Moderate (all)
						Standard care	31	45.20 (14.7)	48.3	
**Cheng L, 2020**	China	OL	Hospitals (multicentre)	2	21, 28, 60	Human-granulocyte-colony–Stimulating Factor (rhG-CSF)	100	Median: 45 (40–55)	58	Moderate to severe (NR)
						Standard care	100	Median 46 (38–54)	54	
**Chowdhury, 2020**	Bangladesh	NR	Outpatients (single-centre)	10	35	Ivermectin plus doxycycline	63	35.72 (15.1)	71.7	Mild (all)
						Hydroxychloroquine plus azithromycin	62	31.9 (12.72)	83.9	
**Corral-gudino, 2020**	Spain	OL^f^	Hospitals (multicentre)	6	28	Corticosteroid (metylprednisolone)	34	73 (11)	68	Severe (all)
						Standard care	29	66 (12)	55	
**Dabbous, 2020**	Egypt	OL	Hospitals (multicentre)	10	30	Favipiravir	50	36.3 (12.5)	50	Mild (NR), moderate (NR)
						Standard care*	50	36.4 (11.5)	50	
**Davoudi-monfared, 2020**	Iran	OL	Hospital (single-centre)	14	28	Interferon β-1a	46	56.50 (16)	52.4	Moderate (NR) to critical (NR)
				7–10 days		Standard care	46	59.53 (14)	56.4	
**Deftereos, 2020**	Greece	OL	Hospital (multicentre)	21	21	Colchicine	56	63 (55–70)	56	Severe (NR), moderate (NR)
						Standard care	50	65 (54–80)	60	
**Dequin, 2020**	France	DB	Hospitals (multicentre)	14	28	Corticosteroid (hydrocortison)	76	63.1	71.1	Severe (28), critical (121)
						Placebo	73	66.3	68.5	
**Duarte, 2020**	Argentina	OL	Hospitals (multicentre)	14	30	Telmisartan	41	60 (17.8)	67.5	NR
						Standard care	41	63.8 (18.7)	55.3	
**Dubèe, 2020**	France	DB	Hospitals (multicentre)	9	28	Hydroxychloroquine	125	Median: 76 (60–85)	52	Mild (99), moderate (151)
						Standard care	125	Median: 78 (57–87)	44.8	
**Edalatifard, 2020**	Iran	SB	Hospitals (multicentre)	3	60	Corticostroid (methylprednisolone)	34	55.8 (16.3)	70.6	Severe (all)
						Standard care	34	61.7 (16.6)	53.6	
**Entrenas castillo, 2020**	Spain	OL	University hospital (single-centre)	Until discharge	28	Calcifediol	50	53.1 (10.8)	54	Moderate to severe (NR)
						Standard care	26	53.8 (9.3)	69	
**Esquivel-moynelo, 2020**	Cuba	OL	Hospital (single-center)	14	14	Interferon α 2b plus interferon γ	41	Median 42 (19–82)	46.7	Mild (NR) moderate (NR)
						Interferon α 2b	38	Median 31 (19–57)	60.6	
**Furtado, 2020**	Brazil	OL	Hospitals (multicentre)	10	29	Hydroxychloroquine plus azithromycin	237	Median: 59.4 (49.3–70)	65	Moderate to critical (NR)
						Hydroxychloroquine	210	Median 46 (38–54)	67	
**Gharbharan A, 2020**	Netherlands	OL	Hospitals (multicentre)	NR	60	Convalescent plasma	43	63 (55–77)	77	Moderate (NR), critical (NR)
						Standard care	43	61 (56–70)	67	
**Gharebaghi, 2020**	Iran	DB	Hospital (single-centre)	3	NR	Immunoglobulin	30	55.5 (45.6)	70	Severe (all)
						Placebo	29	56 (47.7)	68.9	
**Guvenmez, 2020**	Turkey	OL	Hospital (single-centre)	5	6	Lincomycin	12	58.4 (15.4)	66.7	Moderate (all)
						Azithromycin	12	59.1 (16.6)	58.3	
**Hashim, 2020**	Iraq	NR	Hospital (critical and severe ill)/Outpatients (mild/moderate)	10	NR	Ivermectin + Doxycycline	70	50.1 (9.3)	53	Mild/moderate (48), severe (11), critical (11)
						Standard care	70	47.2 (7.8)	51	Mild/moderate (48), severe (11)
**Hermine 2020**	France	OL	Hospitals (multicentre)	1	90	Tocilizumab	64	Median: 64 (57.1–74.3)	70	Moderate (NR), severe (NR)
						Standard care	67	Median: 63.3 (57.1–72.3)	66	
**Huang, 2020**	China	OL	Hospital (single-centre)	10	14	Chloroquine	10	41.5 (33.8–50)	30.0	Severe (3); moderate (7)
						Lopinavir/ritonavir	12	53.0 (41.8–63.5)	50.0	Severe (5); moderate (7)
**Huang Y-Q, 2020**	China	OL	Hospital (single-centre)	14	28	Ribavirin	33	40.3 (12.5)	55	Moderate (all)
						Lopinavir/ritonavir plus interferon α	36	43.3 (10.4)	53	
						Ribavirin plus lopinavir/ritonavir plus interferon α	32	43.8 (11.7)	28	
**Hung, 2020**	China	OL	Hospitals (multicentre)	14	14	Lopinavir/ritonavir + ribavirine + interferon β-1b	86	51 (31–61.3)	52.0	Mild (NR); moderate (NR)
						Lopinavir/ritonavir	41	52 (33.5–62.5)	56.0	
**Ivashchenko, 2020**	Russia	OL	Hospitals (multicentre)	14	29	Favipiravir (1,600/600 mg)	20	51 (15.6)	40	Moderate (all)
						Favipiravir (1800/800 mg)	20	52.6 (15)	65	
						Standard care	20	48.6 (16.1)	45	
**Jagannathan, 2020**	United States	SB	Outpatients	1	28	Peginterferon Lambda-1a	60	Median: 37 (18–66)	60	Mild/moderate (all)
						Placebo	60	Median: 34 (20–71)	54	
**Jeronimo, 2020**	Brazil	DB	Hospital (single-centre)	5	28	Corticosteroid	209	54 (14.9)	65.9	Moderate to critical (NR)
						Placebo	207	56 (15.5)	64.7	
**Kamran, 2020**	Pakistan	OL	Hospital (single-centre)	5	14	Hydroxychloroquine	349	34 (11.8)	93.2	Mild (all)
						Standard care	151	34 (9.8)		
**Khamis, 2020**	Oman	OL	Hospital (single-centre)	10 + 5	14	Favipiravir plus interferon β 1b	44	54 (15)		Moderate to severe (NR)
				8		Hydroxychloroquine	45	56 (16)		
**Krolewiecki, 2020**	Argentina	OL	Hospitals (multicentre)	5	30	Ivermectin	30	42.3 (12.8)	50	Mild/moderate (all)
						Standard care	15	38.1 (11.7)	67	
**Kumar, 2020**	India	OL	Hospitals (multicentre)	NR	30	Itolizumab	22	49.55 (12.49)	95	Severe (all)
						Standard care	10	48.3 (14.62)	70	
**Lenze, 2020**	United States	DB	Outpatients	15	15	Fluvoxamine	80	Median: 46 (35–58)	30	NR
						Placebo	72	Median: 45 (36–54)	26	
**Li L, 2020**	China	OL	Hospital (multicentre)	2–3 (hours)	28	Convalescent plasma	52	70 (62–80)	59.9	Severe (45), critical (58)
						Standard care	51	69 (63–76)	64.7	
**Li T, 2020**	China	OL	Hospital (single-centre)	14	28	Bromhexine hydrochloride	12	Median: 53	83.3	Mild/moderate (NR)
						Standard care	6	Median: 47	66.7	
**Lopes, 2020**	Brazil	DB	Hospital (NR)	10	28	Colchicine	19	Median: 48 (41.5–64)	52.9	Moderate to severe (NR)
						Placebo	19	Median: 53 (35.5–65.5)	27.8	
**Lou Y, 2020**	China	OL	Hospital (single-centre)	7	14	Baloxavir	10	53.5 (12.5)	70.0	Moderate (NR); severe (NR); critical (NR)
						Favipiravir	10	58 (8.1)	77.0	
						Existing antiviral treatment	10	46.6 (14.1)	70.0	
**Maldonado, 2020**	Mexico	NR	Hospital (single-centre)	Until discharged	Until discharged	Pentoxyfilline	36	55.3 (9.2)	53.8	NR
						Standard care	18	62.3 (15.3)	58.3	
**Mansour, 2020**	Brazil	OL	Hospital (single-centre)	4	28	Icatibant	10	51.6 (9.1)	70	Severe (all)
						Inhibitor of C1 esterase/kallikrein	10	54.4 (14.8)	40	
						Standard care	10	48.9 (10.5)	50	
**Mehboob, 2020**	Pakistan	OL	Hospital (single-centre)	3–5	5	Aprepitant plus corticosteroid	8	47.63 (12.1)	37.5	Moderate (5), severe (6), critical (7)
						Corticosteroid	10	60.9 (9.8)	80	
**Miller, 2020**	United States	OL	Hospitals (multi-centre)	3	28	Auxora^e^	20	59 (12); 64 (14)e	41, 33	Severe (all)
						Standad care^e^	10	61 (13), 36e	56, 100	
**Mitijà O, 2020**	Spain	OL	Outpatients	7	14	Hydroxychloroquine	136	41.6 (12.4)	72.1	Mild (all)
						Standard care	157	41.7 (12.6)	65.6	
**Monk, 2020**	United Kingdom	DB	Hospitals (multi-centre)	14	28	Interferon β 1a	50	57.8 (14.6)	56	Mild/moderate (11), severe (37)
						Placebo	51	56.5 (11)	62	Mild/moderate (21), severe (29)
**Morteza, 2020**	Iran	OL/DB	Hospital (NR)	5	NR	Ivermectin (200 mg/kg)	30	Median:61 (42–69)	40	Mild/moderate (29), severe (1)
						Ivermectin (200,200,200 mg/kg)	30	Median: 53 (47–60)	63.3	Mild/moderate (22), severe (26)
						Ivermectin (400 mg/kg)	30	Median: 54 (46–65)	53.3	Mild/moderate (25), severe (5)
						Ivermectin (400,200,200 mg/kg)	30	Median: 54 (46–65)	43.3	Mild/moderate (25), severe (5)
						Standard care	30	Median: 55 (45–70)	53.3	Mild/moderate (27), severe (3)
						Placebo	30	Median: 58 (45–68)	46.7	Mild/moderate (28), severe (2)
**Nojomi, 2020**	Iran	OL	Hospitals (multicentre)	7–14	30	Umifenovir plus hydroxychloroquine	50	56.6 (17.8)	66	Mild (9), moderate (29), severe (12)
						Lopinavir/ritonavir plus hydroxychloroquine	50	52.6 (14.8)	54	Mild (10), moderate (29), severe (11)
**Omrani, 2020**	Qatar	DB	Outpatients	7	21	Hydroxychloroquine + Azitromycin	152	Median: 42 (38–48)	98.7	Mild (all)
						Hydroxychloroquine	152	Median: 40 (31–47)	98	
						Placebo	152	Median: 41 (31–47)	98.7	
**Pan (SOLIDARITY trial), 2020**	Albania, Argentina, Austria, Belgium, Brazil, Canada, Colombia ecypt, Honduras, India, Indonesia, Iran, Ireand, Italy, kwait, Lebanon, Luxembourg, Lithuania, Malaysia, north Macedonia, Pakistan, Norway, Peru, Philippines, Saudi Arabia, soputh africa, Spain, Switzerland	OL	Hospitals (multicentre)	10, 14, 6	28	Remdesivir	2750	NR	62.2	Mild/moderate (4964), severe (487)
						Standard care	2725		63.7	
						Hydroxychloroquine	954		60.6	Mild/moderate (1,686), severe (167)
						Standard care	909		59	
						Lopinavir-ritonavir	1,411		60.8	Mild/moderate (2545), severe (226)
						Standard care	1,380		58.5	
						Interferon beta 1a	2050		63.6	Mild/moderate (3831), severe (269)
						Standard care	2064		62.3	
**Rahamani, 2020**	Iran	OL	Hospitals (multicentre)	14	28	Interferon β 1b	40	Median: 60	60.6	NR
						Standard care	40	Median: 61	57.6	
**Ray, 2020**	India	OL	Hospital (NR)	1	30	Convalescent plasma	40	Total: 61.43 (11.33)	75	Severe (all)
						Standard care	40		67.5	
**Recovery trial, 2020**	United Kingdom	OL	Hospital (multicentre)	10	28	Hydroxychloroquine	1,561	65.2	62	Moderate (NR) to critical (NR)
						Standard care	3155	65.4	63	
						Dexamethasone	2104	66.9	64	
						Standard care	4321	65.8	64	
						Lopinavir-ritonavir	1,596	NR	NR	
						Standard care	3376	NR	NR	
**REMAP-CAP trial, 2020**	United Kingdom, Europe, Australia	OL	ICU (multicentre)	7	21	Corticosteroid (Hydrocortisone)_fixed dose	143	60.1 (15.8)	59.6	Severe (all)
						Corticosteroid (Hydrocortisone)_shock-dependent	152	62.7 (13.1)	65.6	
						Standard care	108	60.1 (15.8)	59.6	
**Ren, 2020**	China	OL	Hospital (single-centre)	5	NR	Azvudina	10	Median: 52 (17–61)	60	Mild (3), moderate (17)
						Standard care	10	Median: 50.5 (29–76)	60	
**Rocco, 2020**	Brazil	DB	Outpatient	5	6	Nitazoxanide	238	18–77	52	Mild/moderate (all)
						Placebo	237	18–77	42	
**Rosas, 2020**	Canada, Denmark, France, Germany, Netherlands, Spain, United States	DB	Hospitals (multicentre)	7	28, 60	Tocilizumab	301	60.9 (14.6)	69.7	Severe (all)
						Placebo	151	60.6 (13.7)	70.1	
**Ruzhentsova, 2020**	Russia	OL	Outpatients/hospitals (multicentre)	10	28	Favipiravir	112	41.7 (10.6)	43.8	Mild/moderate (all)
						Standard care	56	42 (10.4)	53.6	
**Sadeghi, 2020**	Iran	OL	Hospitals (multicentre)	14	30	Sofosbuvir plus daclastavir	35	Median: 58	61	Moderate (NR), severe (NR)
						Standard care	35	Median: 62	42	
**Sakoulas, 2020**	United States	OL	Hospitals (multicentre)	3	30	Intravenous immunoglobulin	17	56.6 (17.8)	66	Moderate (NR), severe (NR)
						Standard care	17	52.6 (14.8)	54	
**Salama, 2020**	United States, Mexico, Kenya, South Africa, peri, Brazil	DB	Hospitals (multicentre)	1	28, 60	Tocilizumab	259	56 (14.03)	60.2	Severe (all)
						Placebo	129	55.6 (14.9)	57	
**Salvarani C, 2020**	Italy	OL	Hospitals (multicentre)	8–12 (hours)	30	Tocilizumab	60	Median: 61.5 (51.5–73.5)	66.7	Severe (all)
						Standard care	66	Median: 60 (54–69)	56.1	
**Sekhavati, 2020**	Iran	OL	Hospital (single-centre)	5	30	Azytromicin plus lopinavir/ritonavir plus hydroxychloroquine	56	54.4 (15.9)	50	NR
						Lopinavir/ritonavir plus hydroxychloroquine	55	59.9 (15.5)	41.8	
**Self, 2020**	United States	DB	Hospitals (multicentre)	5	28	Hydroxychloroquine	242	Median: 58 (45–69)	55.8	Severe (all)
						Placebo	237	Median: 57 (43–68)	55.7	
**Shi, 2020**	China	DB	Hospital (single-centre)	6	28	Umbilical cord_ mesenchymal stem cells (hUC-MSC)	66	60.7 (9.1)	56.9	Severe (all)
						Placebo	35	59.9 (7.8)	54.3	
**Shu, 2020**	China	OL	Hospital (single-centre)	5	30	Umbilical cord_ mesenchymal stem cells (hUC-MSC)	12	61 (17.9)	66.7	Mild (3), moderate (28), severe (10)
						Standard care	29	57.9 (15.8)	51.2	
**Simonovic, 2020**	Argentina	DB	Hospitals (multicentre)	1	30	Convalescent plasma	228	Median: 62.5 (53–72.5)	70.6	Severe (all)
						Placebo	106	Median: 62 (49–71)	61	
**Spinner, 2020**	United States, Italy, Spain, Germany, Hong Kong, Singapore, South Korea, and Taiwan	OL	Hospitals (multicentre)	5–10	11	Remdesivir 5 days	197	Median: 56	61	Moderate (all)
						Remdesivir 10 days	199	Median: 58	60	
						Standard care	200	Median: 57	63	
**Stone, 2020**	United States	DB	Hospitals (multicentre)	1	28	Tocilizumab	161	Median: 61.6 (46.4–69.7)	60	Moderate (NR), severe (NR)
						Placebo	82	Median: 56.5 (44.7–67.8)	55	
**Tabarsi, 2020**	Iran	OL	Hospital (single-centre)	14	NR	Immunoglobulin	52	54.29 (12.85)	76.9	Severe (all)
						Standard care	32	52.47 (14.49)	78.1	
**Tang, 2020**	China	OL	Hospitals (multicenter)	14–21^b^	28	Hydroxychloroquine	75	48 (14.1)	56.0	Severe (1); moderate (59); mild (15
						Standard care	75	44.1 (15)	53.0	Severe (1); moderate (67); mild (7)
**Tomazini, 2020**	Brazil	OL	ICU (multicentre)	10	28	Corticosteroid (dexamethasone)	151	60.1 (15.8)	59.6	Critical (all)
						Standard care	148	62.7 (13.1)	65.6	
**Udwadia, 2020**	India	OL	Hospitals (multicentre)	14	28	Favipiravir	75	43.6 (12.2)	70.8	Mild (47), moderate (28)
						Standard care	75	43 (11.7)	76	Mild (45), moderate (30)
**Ulrich, 2020**	United States	DB	Hospitals (multicenter)	6	14, 30	Hydroxychloroquine	67	65.5 (16.4)	67.2	Mild (NR), moderate (NR), severe (NR)
						Standard care	61	65.8 (16)	50.8	
**Vlaar, 2020**	Netherlands	OL	Hospital (single-centre)	22	28	Anti-c5a antibody (IFX-1)	15	58 (9)	73	Moderate (4), severe (8), critical (18)
						Standard care	15	63 (8)	73	
**Wang, 2020**	China	DB	Hospital (multicentre)	10	28	Remdesivir	158	Median 66	56.0	Severe (all)
						Placebo	79	Median 64	65.0	
**Wang D, 2020**	China	OL	Hospitals (multicentre)	1	14	Tocilizumab	33	Median: 65.3 (58–71)	69.7	Moderate (37), severe (28)
						Standard care	32	Median: 63 (54–69	70.1	
**Wang M, 2020**	China	OL	University hospital (single-centre)	10	60	Leflunomide + Interferon α 2a	26	Median: 56 (43–67.3)	54.2	NRg
						Interferon α 2a	24	55.5 (47.8–66.5)	37.5	
**Wu, 2020**	China	DB	Emerrgency dept., isolation wards, ICU (multicentre)	7	28	Triazavirin	26	Median: 53 (46–62)	53.9	Mild to severe
						Placebo	26	Median 59 (51–69)	46.1	
**Yakoot, 2020**	Egypt	OL	Hospital (NR)	10	21	Sofosbuvir + daclastavir	44	Median: 48 (34–59)	41	Mild (6), moderate (30), severe (8)
						Standard care	45	Median: 50 (31–60)	45	Mild (6), moderate (31), severe (8)
**Yueping, 2020**	China	OL	Hospital (single-centre)	14	21	Lopinavir/ritonavir	34	50.7 (15.4)	50	Mild (11); moderate (NR)
						Umifenovir	35	50.5 (14.6)	45.7	
						Standard care	17	44.3 (13.1)	41.2	
**Zhao, 2020**	China	OL	Hospitals (multicentre)	7	60	Favipiravir + tocilizumab	14	Median: 75 (34–81)	42.9	Moderate to critical (NR)
						Favipiravir	7	Median: 70 (45–89)	71.4	
						Tocilizumab	5	Median: 71 (48–77)	60	
**Zheng, 2020**	China	OL	Hospitals (multicentre)	7–10^a^	9	Novaferon	30	50.1	56.7	Severe (2); moderate (28)
						Novaferon plus lopinavir/ritonavir	30	48.8	43.3	Severe (2); moderate (28)
						Lopinavir/ritonavir	29	41.1	41.4	Severe (1); moderate (28)

Note: DB= double blind, NR=not reported, OL= open label, SB=single blind *: in some studies the information was reported only for the analysed participants (e.g. ITT population), a:172 in Hydroxychloroquine plus Azithromycin arm, 159 Hydroxychloroquine arm and 173 Standard care confirmed with COVID-19 by RT-PCR test b: the course of treatment in both groups was 7-10 days. c: the course of treatment in moderate patients was 14 day and in severe patients was 21 days; d: in Standard care arm the course of treatment was 7-10 days; e: 26 patients received low flow supplemental oxygen (17 assigned to Auxora, 9 assigned to SC) and 4 patients received high flow supplemental oxygen (3 assigned to Auxora, 1 assigned to SC); f:partially randomized controlled trial. g: prolonged PCR positivity. *: quote: “50 patients who received oseltamivir 75 mg 12 hourly for 10 days and hydroxychloroquine 400 mg 12 hourly on day-one followed by 200 mg 12 hourly daily on day-2 to10 days conforming to the national.

The mean study sample size was 343 participants (SD 1312). In total, 21,846 participants were randomly assigned to an active drug (see Supplementary Appendix S7 in the supplementary material) and 12,655 were randomly assigned to placebo or SC. The mean age was 51.7 years (SD 8.4), while two third (40.8%) of the sample population were women. The average duration of the treatment in the studies was 7.9 days (SD 4.8), while the average duration of follow up was 26.1 days (SD 12.9). The evaluation of transitivity assessment was evaluated in all trials included in the network irrespectively of the outcome being reported for the following effect modifiers: age, gender, disease severity (mild to moderate, severe, critical) and is reported in Supplementary Appendix S5.

Seventy-two studies compared active drugs only with SC or placebo, eighteen studies compared active drugs only with other active drugs and six three-arm studies compared active drugs with other active drugs and with SC or placebo. Most of the studies were conducted in China (25 of 96), thirteen studies were conducted in Europe (i.e. France, Greece, Italy, Netherlands, Spain, United Kingdom). Eleven studies were conducted in United States, eleven in Iran, seven studies in Brazil, five in India, four in Egypt and three in Argentina. Two studies were conducted in Russia and two in Pakistan, while six studies were intercontinental. Other nine countries contributed to the pool of the evidence with one study each (see Table 1 for more details). In terms of risk of bias, 35% of the RCTs were at high risk of bias (34 of 96), 44% were at unclear risk of bias (42 of 96) and 21% at low risk of bias (20 of 96) (See Supplementary Appendix S6 for the full risk of bias assessment).

**FIGURE 2:**
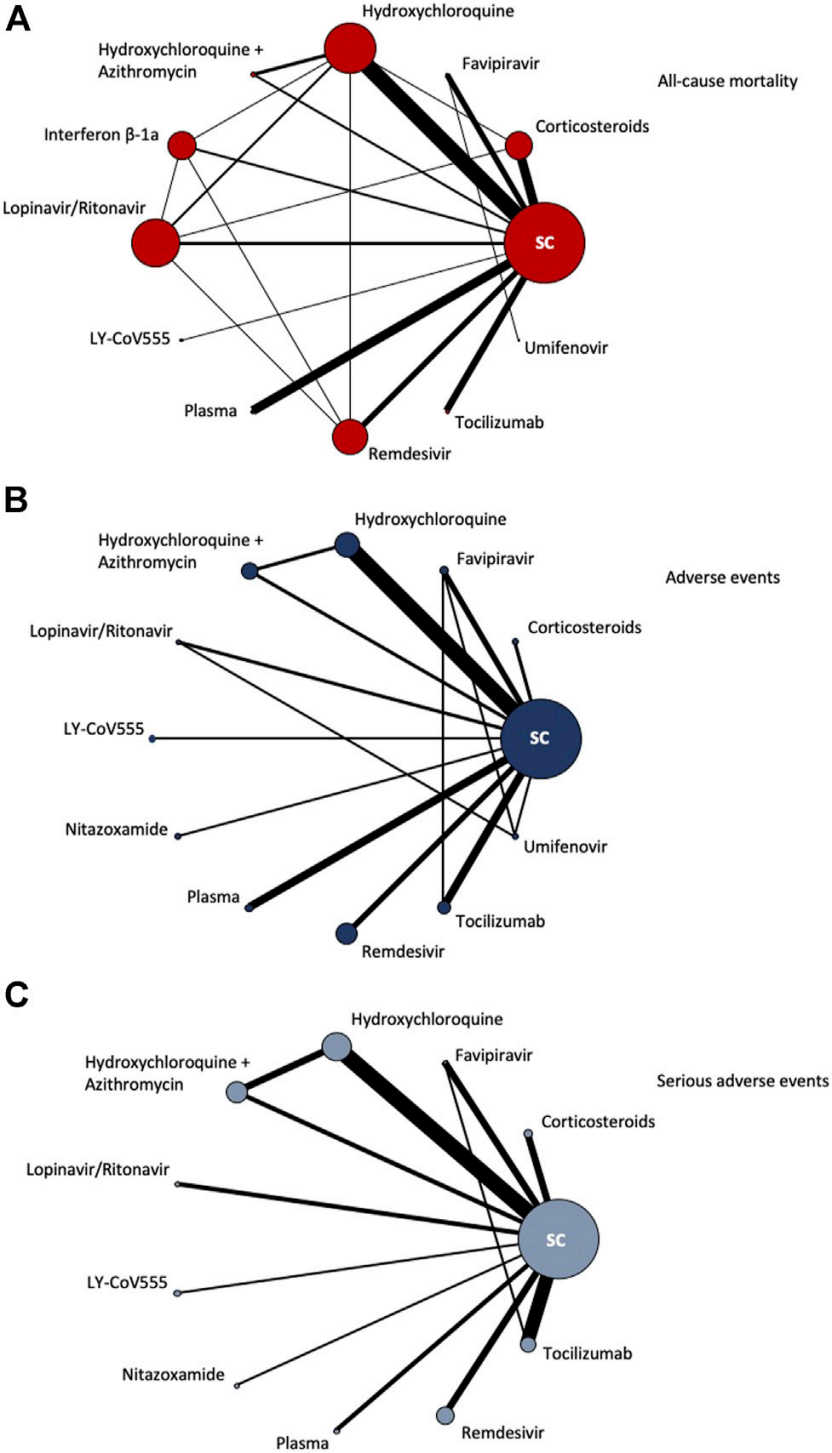
Network of eligible comparisons for all-cause mortality, adverse events and serious adverse events. The figure plots the network of eligible direct comparisons for all-cause mortality (42 studies) **(A)**, adverse events (30 studies) **(B)** and serious adverse events (30 studies) **(C)**. The width of the lines is proportional to the number of trials comparing every pair of treatments, and the size of every node is proportional to the number of randomized participants.

Figure 2 shows the network of eligible comparisons for all-cause mortality, adverse events and serious adverse events. An analysis of the geometry of the network showed a well-connected polygon for all-cause mortality, with some single-connected nodes which included LY-CoV555, plasma, tocilizumab and umifenovir. The single-connected nodes are poorly connected to the rest of the network and will provide more imprecise estimates. For the safety outcomes (i.e. AEs and SAEs), we can see from Figure 2 more single-connected nodes and overall poorer connected networks which therefore depended extensively on indirect comparisons.

### Pairwise Meta-Analysis

The pairwise meta-analysis and data on heterogeneity are presented in the Supplementary Material (Supplementary Appendix S7). The pairwise meta-analysis for the primary outcomes showed a reduction of all-cause mortality for Human-Granulocyte-Colony–Stimulating Factor (rhG-CSF) (RR 0.25, 95%CI 0.07 to 0.86, 1 RCT, *n* = 200) compared to SC. Regarding safety, a number of pharmacological interventions were worse than SC in terms of adverse events, including colchicine (RR 2.17, 95%CI 1.29–3.65), hydroxychloroquine (RR 1.99, 95%CI 1.13–3.51), the combination of hydroxychloroquine and azithromycin (RR 1.39, 95%CI 1.06–1.82), rhG-CSF (RR 2.02, 95%CI 1.62–2.50). In terms of serious adverse events remdesivir was safer than SC (RR 0.75, 95%CI 0.63–0.89).

Regarding secondary outcomes, the pairwise meta-analysis showed that azvudine, nitazoxamide and convalescent plasma were better than SC in terms of SARS-CoV-2 clearance rate (RR ranging from 1.6 to 2.33). Telmisartan and tocilizumab compared to SC reduced length of hospital stay (HR 2.02 and 1.24, respectively). Hydroxychloroquine and ruxolitinib compared to SC showed in one small trial each to improve pulmonary disease in CT imaging (RR 3.80 and 1.45, respectively). In one RCT, rhG-CSF had a reduction in the progression of COVID-19 disease when compared to SC (RR 0.13, 95%CI 0.03–0.57). Remdesivir and telmisartan were superior compared to SC for number of patients discharged from hospital (RR 1.13 and 1.61, respectively).

### Network Meta-Analysis

**FIGURE 3:**
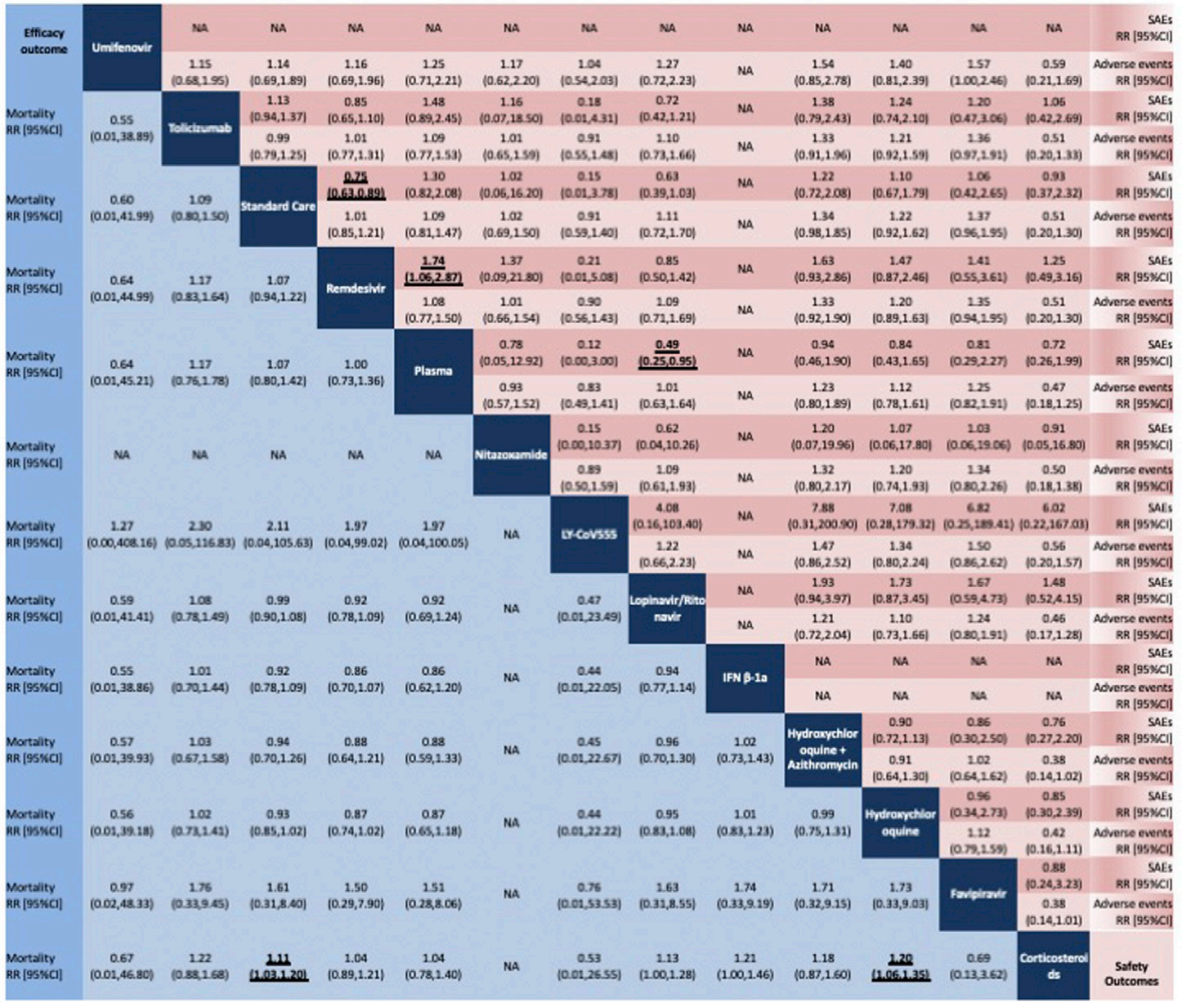
Network meta-analysis of all-cause mortality (blue), adverse events (light red) serious adverse events (red). Pharmacological treatments are reported in alphabetical order. Comparisons should be read from left to right. All-cause mortality and safety estimates are located at the intersection between the column-defining and the row-defining treatment. For all-cause mortality, RRs above 1 favor the column-defining treatment. For safety, RRs above 1 favor the row-defining treatment. We incorporated the GRADE judgments in the figure. Estimates in gray have a very low or low certainty of evidence.

The results of the network meta-analysis are presented in Figure 3 for the primary outcomes. In terms of all-cause mortality, we evaluated 42 studies. When compared to SC or placebo, only corticosteroids significantly reduced the mortality rate (RR 0.90, 95%CI 0.83 to 0.97, moderate certainty of evidence). Corticosteroids significantly reduced the mortality rate also when compared to hydroxychloroquine (RR 0.83, 95%CI 0.74 to 0.94, moderate certainty of evidence).

In terms of AEs, we evaluated 30 studies. No significant differences were found between the included compounds. Remdesivir proved to be better in terms of SAEs (30 studies included in the whole network) when compared to SC or placebo (RR 0.75, 95%CI 0.63 to 0.89, high certainty of evidence) and plasma (RR 0.57, 95%CI 0.34 to 0.94, high certainty of evidence). The combination of lopinavir and ritonavir proved to reduce SAEs when compared to plasma (RR 0.49, 95%CI 0.25 to 0.95, low certainty of evidence). The global inconsistency was not significant for all the outcomes considered (See Supplementary Appendix S9). Tests of local inconsistency did not show any inconsistent loops (See Supplementary Appendix S9). The comparison-adjusted funnel plots of the network meta-analysis were suggestive for some publication bias for all-cause mortality (42 studies evaluated) (see Supplementary Appendix S10). Few studies reported similar comparisons for AEs and SAEs (30 studies evaluated for both AEs and SAEs), which makes difficult the interpretation of the funnel plots for safety outcomes. Supplementary Appendix S11 in Supplementary Material presents the ranking of treatments based on cumulative probability plots and SUCRAs.

The certainty of evidence for the relative treatment effects of all-cause mortality and safety outcomes varied from high to very low (See Supplementary Appendix S12).

### Subgroup Analysis

**FIGURE 4:**
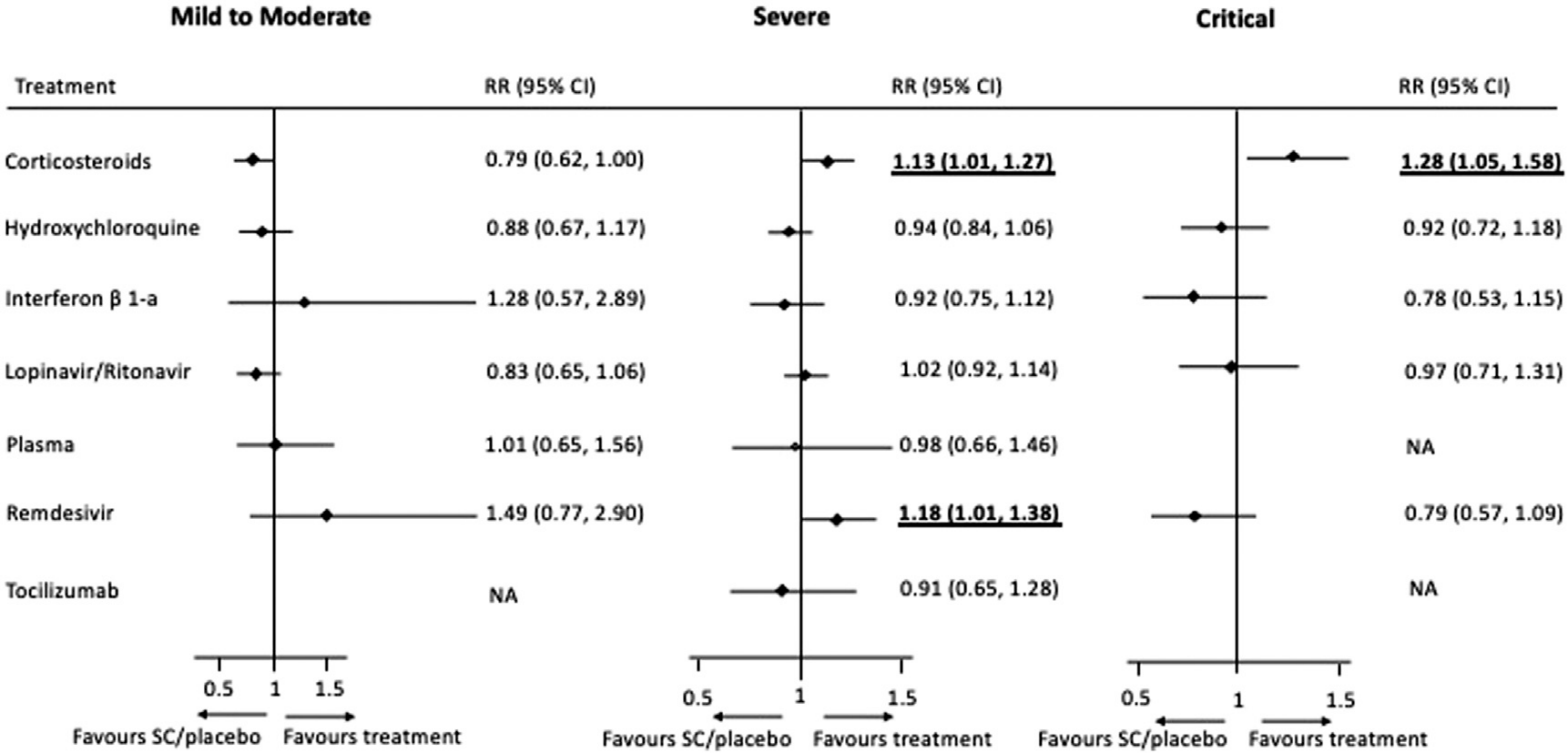
Forest plots of network meta-analysis by subgroup for disease severity: Mild to Moderate (17 trials), Severe (21 trials), and Critical (9 trials). Treatments are compared for each treatment to standard care or placebo, and performed for treatments with >100 individuals randomised.

The subgroup network meta-analysis for all-cause mortality according to the severity of disease showed a positive effect for corticosteroids compared to SC or placebo for individuals with a severe (RR 1.13, 95% CI 1.01–1.27) or critical condition (RR 1.28, 95%CI 1.05–1.58). Remdesivir showed to be effective compared to SC or placebo only for individuals with a severe condition (RR 1.18, 95%CI 1.01–1.38). No pharmacological treatments proved to be useful for individuals with a mild to moderate disease (see Figure 4).

## Discussion

This study includes 96 trials randomising a total of 34,501 patients to receive one of 59 therapeutic options and comparing these to either SC or placebo. This is part of a living systematic review and network meta-analysis previously registered on Prospero (number CRD42020176914) investigating pharmacological interventions against Covid-19 and encompasses all of the comparative RCTs until this point (December 10, 2020). The 59 options comprise both single agents and combination therapies. Our work is registered as a prospective network meta-analysis, which confers several advantages over the more common practice of meta-analyses done on retrospective collection of RCTs. It is a living study and so will extend synchronously with the evidence as new data is published. Our results come at an opportune time because clinical practice can be informed based on the evidence already available.

We found that corticosteroids reduced all-cause mortality in patients with Covid-19. Remdesivir was safer than SC in terms of SAEs, while no treatment proved superiority over others in terms of AEs. High value and clinically important objective outcomes were chosen in the form of mortality, adverse events and serious adverse events in order to give this study credence and help us to make clearer recommendations.

In general, according to our analysis, we can recommend corticosteroids as they reduced mortality significantly with a moderate certainty of evidence. However, based on our subgroup analysis we would recommend corticosteroids only for individuals with a severe or critical disease as they did not prove to be superior to SC or placebo for individuals with a mild to moderate disease.

There are a plethora of secondary outcomes of lesser importance than mortality. Other agents have appeared superior in these outcomes however paint an unconvincing picture with a low certainty of evidence.

Recently, several systematic reviews on the effectiveness of pharmacological compounds for Covid-19 have been published. This report has several originalities: it focused on all pharmacological treatments now under investigation, compared versus placebo, standard care or active control; it was the result of one of the first protocols on this subject registered on the Prospero database (CRD42020176914); it produced continuous analyses which were integrated into a platform ready to be used by decision-makers in the context of this pandemic (https://eunethta.eu/covid-19-treatment/). Our data are consistent with a recent systematic review that summarised evidence about the benefits and harms of hydroxychloroquine or chloroquine for the treatment or prophylaxis of Covid-19 either from observational and randomised clinical trials (Hernandex et al., 2020). The authors concluded that evidence on the benefits and harms of using hydroxychloroquine or chloroquine to treat COVID-19 was very weak and conflicting. Our study differs with another network meta-analysis that was recently published (Siemieniuk et al., 2020). One of the differences it that our network analysis was performed under a frequentist framework and the other was a Bayesian. A second difference is that our search strategy is more recent, extending to include studies for one month later. Moreover we included important unpublished data that were not included by Siemieniuk et al. (Siemieniuk et al., 2020), such as the SIMPLE trial (Spinner et al., 2020) and the hydroxychloroquine and lopinavir/ritonavir arms of the RECOVERY trial (Horby et al., 2020; RECOVERY Collaborative Group, 2020). We are aware of an initiative that has been taken by some Cochrane groups which performs comprehensive and living systematic reviews and network meta-analyses of preventative treatment, rehabilitation, pharmacological and non-pharmacological treatments for Covid-19 (Boutron et al., 2020 - available at: https://covid-nma.com/). The aim of this systematic review is more targeted to pharmacological treatments.

Our study has some limitations.

Firstly, outcomes are not being consistently reported by different trials and although we included a total of 96 RCTs, only 42 studies were used in the network meta-analysis for all-cause mortality, 30 in the network meta-analysis for adverse events and 30 in the network meta-analysis for serious adverse events.

A second limitation of our work is the small number of compounds currently included in our network meta-analysis, due to the low number of patients randomised to many treatments. Despite this several significant results were able to be achieved. Although we currently face a limitation in that many eligible studies are not numerous, this living study will become more substantial and comprehensive as time progresses. This will allow the evidence base to be drawn from an ever-greater number of studies. Studies are being released at a rapid rate reducing bias from differing times of data collection. In this context, we will be able to produce comparative evidence earlier and more efficiently as new evidence is published.

A third limitation to consider is that a number of the trials we have used are unpublished. On a positive note it is helpful to extract unpublished data because it gives us more information, however this might potentially produce less reliable analysis as results have not been through the process of peer review (Zhao et al., 2021). We plan to conduct in future a meta-regression to evaluate the impact of unpublished data on the effect estimates.

Fourth, ‘standard care’ is heterogeneously defined and can consist of supportive care with intravenous resuscitation fluid, antibiotics, analgesics and anti-pyretics but also antiviral agents and glucocorticoids. This means that some drugs that are used as experimental in some trials are used as SC. One reason for this is that many clinicians have resorted to using off-label medications with a lack of other viable options. This could create a confounder for the trial analysis and can dampen the internal validity of the trial.

Fifth, only few of the trials were double-blinded, while most were open-label. This resulted in a high rating for risk of bias and low certainty of evidence according to GRADE. However given our objective outcome measures this will be less relevant than if our outcome measures had been subjective.

We believe that the results of our research can be informative for patients, clinicians and policy-makers. Corticosteroids reduced all-cause mortality with a moderate certainty of evidence compared to SC in individuals with a severe or critical disease. The safety profile of remdesivir was better than SC and we have also a moderate certainty of evidence that hydroxychloroquine and lopinavir/ritonavir do not affect all-cause mortality compared to SC. Corticosteroids were better than hydroxychloroquine for all-cause mortality with moderate certainty of evidence. Data emerging from observational studies culminated in regulatory decisions by World health Organization and national authorities that limited the use of hydroxychloroquine outside clinical trials (Ledford, 2020). Our analysis supports this decision overcoming several potential biases associated with analysis based on observational studies. However, debate as to which patients should receive hydroxychloroquine is continuing. Results from rhG-CSF are encouraging but we must wait for further research before commenting on whether they affect mortality as it was studied in one small RCT. Clearly, drugs repurposed for the treatment of Covid-19 showed limited effectiveness (Kotecha et al., 2020).

We registered this as a prospective study in order to capitalise on the benefits that this provides. Consistently agreed outcome measures between researchers is one of these, and as this living study proceeds, we hope to attain that. The differentiation of patients by mild, moderate and severe disease would also be helpful. Future research should be prospectively planned in this way, and refocused in a coordinated effort to improve critical patient outcomes. This was a network meta-analysis of aggregate data which comprises the highest certainty evidence available at the present time. However we would like to stress the importance to researchers of sharing their data which increases transparency. Meta-analysis of individual patient data from RCTs would be the next logical step allowing tailored treatments dependent on patient characteristics.

## Data Availability

Publicly available data were analyzed in this study. Our living review can be found at https://www.deplazio.net/farmacicovid/index.html.
