# Supplementary material for: Comparative Effectiveness of Pharmacological Interventions for Covid-19: A Systematic Review and Network Meta-Analysis
Source: Front Pharmacol. 2021 May 3;12:649472. doi: 10.3389/fphar.2021.649472 (PMC8126885; doi:10.3389/fphar.2021.649472)
Supplement: Supplementary file 1 [file DataSheet1.docx]

**SUPPLEMENTARY MATERIAL**

This supplementary material has been provided by the authors to give readers additional information about their work.

**Supplemental material content**

| Appendix 1 | Protocol and full search strategy | Page 3 |
| --- | --- | --- |
| Appendix 2 | PRISMA NMA Checklist of Items to Include When Reporting A Systematic Review Involving a Network Meta-analysis | Page 7 |
| Appendix 3 | References for included trials | Page 11 |
| Appendix 4 | Additional characteristics of studies included | Page 24 |
| Appendix 5 | Evaluation of the transitivity assumption | Page 43 |
| Appendix 6 | Risk of bias assessment | Page 46 |
| Appendix 7 | Results from pairwise meta-analysis for each outcome: numbers, estimates and heterogeneity | Page 48 |
| Appendix 8 | Network of eligible comparisons for all the studies | Page 59 |
| Appendix 9 | Assessment of inconsistency results for each outcome from the network meta-analysis: global, local | Page 60 |
| Appendix 10 | Comparison-adjusted funnel plot for each outcome from the network meta-analysis | Page 62 |
| Appendix 11 | Treatment ranking and SUCRA plot for each outcome from the network meta-analysis | Page 64 |
| Appendix 12 | Evaluation of the quality of evidence using GRADE framework for primary outcomes | Page 67 |

### **Appendix 1. Protocol and full search strategy**

The protocol was registered on PROSPERO (CRD42020176914).

We report the final protocol here together with amendments included. The original protocol was published on 22nd April 2020 and is available at <https://www.crd.york.ac.uk/PROSPERO/display_record.asp?ID=CRD42020176914>.

**Review question**

Our aim is to suggest clinical recommendations by assessing the comparative effectiveness of pharmacological interventions for the treatment of Covid-19.

**Searches**

We will search the following electronic databases:

• Cochrane Central Register of Controlled Trials (CENTRAL), in the Cochrane Library.

• MEDLINE, accessed via OVID.

• Embase, accessed via OVID.

The searches will cover from the inception of each database and will be updated on a daily basis using auto‐alerts when possible. We will develop search strategies including a combination of controlled vocabulary and free text terms. We will revise the strategy appropriately for each database to take account of differences in controlled vocabulary and syntax rules. We will apply no restriction on language of publication.

We will also search medRxiv Health Sciences and bioRxiv Biology, which provide open access to preprints of preliminary reports of work that have not been peer-reviewed.

In addition to the source and strategies described above, we will screen registers of ongoing studies such as ClinicalTrials.gov and ISRCTN. A similar process will be undertaken twice monthly for the European Clinical Trials Registry.

In the context of living systematic review, we will follow key conferences are to be held and will search conference proceedings when published.

Full search strategy:

**1. PubMed**

(("Coronavirus"[MeSH Terms] OR ("coronavirus*"[Title/Abstract] OR "coronovirus*"[Title/Abstract] OR "coronavirinae*"[Title/Abstract] OR "coronavirus*"[Title/Abstract] OR "coronovirus*"[Title/Abstract] OR "wuhan*"[Title/Abstract] OR "hubei*"[Title/Abstract] OR "Huanan"[Title/Abstract] OR "2019-nCoV"[Title/Abstract] OR "2019nCoV"[Title/Abstract] OR "nCoV2019"[Title/Abstract] OR "nCoV-2019"[Title/Abstract] OR "COVID-19"[Title/Abstract] OR "COVID19"[Title/Abstract] OR "HCoV-19"[Title/Abstract] OR "HCoV19"[Title/Abstract] OR "CoV"[Title/Abstract] OR "2019 novel*"[Title/Abstract] OR "Ncov"[Title/Abstract] OR "n-cov"[Title/Abstract] OR "SARS-CoV-2"[Title/Abstract] OR "SARSCoV-2"[Title/Abstract] OR "SARSCoV2"[Title/Abstract] OR "SARS-CoV2"[Title/Abstract] OR "SARSCov19"[Title/Abstract] OR "SARS-Cov19"[Title/Abstract] OR "SARS-Cov-19"[Title/Abstract] OR "ncorona*"[Title/Abstract]) OR ((("respiratory*"[Title/Abstract] AND ("symptom*"[Title/Abstract] OR "disease*"[Title/Abstract] OR "illness*"[Title/Abstract] OR "condition*"[All Fields])) OR "seafood market*"[Title/Abstract] OR "food market*"[All Fields]) AND ("wuhan*"[Title/Abstract] OR "hubei*"[Title/Abstract] OR "china*"[Title/Abstract] OR "chinese*"[Title/Abstract] OR "huanan*"[All Fields])) OR "severe acute respiratory syndrome*"[All Fields] OR (("corona*"[Title/Abstract] OR "corono*"[All Fields]) AND ("virus*"[Title/Abstract] OR "viral*"[Title/Abstract] OR "virinae*"[All Fields]))) AND ("randomized controlled trial"[Publication Type] OR "controlled clinical trial"[Publication Type] OR "randomized"[Title/Abstract] OR "placebo"[Title/Abstract] OR "clinical trials as topic"[MeSH Terms] OR "randomly"[Title/Abstract] OR "trial"[Title])) NOT ("animals"[MeSH Terms] NOT "humans"[MeSH Terms]) Filters: from 2020/1/1 - 2020/12/10

**2. Ovid MEDLINE(R) ALL)**

1 exp coronavirus/

2 ((corona* or corono*) adj1 (virus* or viral* or virinae*)).ti,ab,kw.

3 (coronavirus* or coronovirus* or coronavirinae* or Coronavirus* or Coronovirus* or Wuhan* or Hubei* or Huanan or "2019-nCoV" or 2019nCoV or nCoV2019 or "nCoV-2019" or "COVID-19" or COVID19 or "CORVID-19" or CORVID19 or "WN-CoV" or WNCoV or "HCoV-19" or HCoV19 or CoV or "2019 novel*" or Ncov or "n-cov" or "SARS-CoV-2" or "SARSCoV-2" or "SARSCoV2" or "SARS-CoV2" or SARSCov19 or "SARS-Cov19" or "SARSCov-19" or "SARS-Cov-19" or Ncovor or Ncorona* or Ncorono* or NcovWuhan* or NcovHubei* or NcovChina* or NcovChinese*).ti,ab,kw.

4 (((respiratory* adj2 (symptom* or disease* or illness* or condition*)) or "seafood market*" or "food market*") adj10 (Wuhan* or Hubei* or China* or Chinese* or Huanan*)).ti,ab,kw.

5 ((outbreak* or wildlife* or pandemic* or epidemic*) adj1 (China* or Chinese* or Huanan*)).ti,ab,kw.

6 "severe acute respiratory syndrome*".ti,ab,kw.

7 or/1-6

8 randomized controlled trial.pt.

9 controlled clinical trial.pt.

10 random*.ab.

11 placebo.ab.

12 clinical trials as topic.sh.

13 random allocation.sh.

14 trial.ti.

15 8 or 9 or 10 or 11 or 12 or 13 or 14

16 exp animals/ not humans.sh.

17 15 not 16

18 7 and 17

19 limit 18 to yr="2020 -Current"

**3. Ovid Embase**

1 exp Coronavirinae/ or exp Coronavirus/

2 exp Coronavirus infection/

3 ((("Corona virinae" or "corona virus" or Coronavirinae or coronavirus or COVID or nCoV) adj4 ("19" or "2019" or novel or new)) or (("Corona virinae" or "corona virus" or Coronavirinae or coronavirus or COVID or nCoV) and (wuhan or china or chinese)) or "Corona virinae19" or "Corona virinae2019" or "corona virus19" or "corona virus2019" or Coronavirinae19 or Coronavirinae2019 or coronavirus19 or coronavirus2019 or COVID19 or COVID2019 or nCOV19 or nCOV2019 or "SARS Corona virus 2" or "SARS Coronavirus 2" or "SARS-COV-2" or "Severe Acute Respiratory Syndrome Corona virus 2" or "Severe Acute Respiratory Syndrome Coronavirus 2").ti,ab,kw. (53293)

4 1 or 2 or 3

5 Clinical-Trial/ or Randomized-Controlled-Trial/ or Randomization/ or Single-Blind-Procedure/ or Double-Blind-Procedure/ or Crossover-Procedure/ or Prospective-Study/ or Placebo/ (2088038)

6 (((clinical or control or controlled) adj (study or trial)) or ((single or double or triple) adj (blind$3 or mask$3)) or (random$ adj (assign$ or allocat$ or group or grouped or patients or study or trial or distribut$)) or (crossover adj (design or study or trial)) or placebo or placebos).ti,ab.

7 5 or 6

8 4 and 7

9 limit 8 to yr="2020 -Current"

**Types of study to be included**

We will include parallel randomised controlled trials. We will not include quasi-randomized controlled trials, cross-over trials, or pilot studies with a single arm.

**Condition or domain being studied**

SARS-CoV-2 is a novel coronavirus causing a respiratory illness termed Covid-19. It started spreading in December 2019, and was declared a pandemic by the World Health Organisation on 11th March 2020. The full spectrum of Covid-19 ranges from mild, self-limiting respiratory tract illness to severe progressive pneumonia, multiorgan failure, and death. There are no registered treatments for coronavirus infections, but some studies, including randomised trials and cohort studies, have already been completed and many more are rapidly developing in an unprecedented effort made by the scientific community.

**Participants/population**

We will include people > 18 years of age affected by COVID-19, as defined by the authors of the studies. There will be no limits in terms of gender or ethnicity or severity of disease.

**Intervention(s), exposure(s)**

We will include studies evaluating interventions for the treatment of people affected by COVID-19, including pharmacological interventions (e.g. antibiotics, antibodies, antimalarial, antiviral, antiretroviral, immunosuppressors/modulators, kinase inhibitors) and their combinations.

**Comparator(s)/control**

Any active treatment, placebo, or standard of care, as defined by study authors.

**Main outcome(s)**

Number of patients experiencing all-cause mortality, adverse events and severe adverse events.

***Measures of effect***

Risk ratio with 95% CIs. We will measure all-cause mortality at the longest follow up and safety (number of patients experiencing any adverse event and serious adverse event) at the end of treatment.

**Additional outcome(s)**

We will give priority according to Core Outcome Set for Clinical Trials on Coronavirus Disease 2019 (Jin et al., 2020), including number of patients with SARS-CoV-2 nasal or pharyngeal swab RT-PCR clearance, time to nasal or pharyngeal swab RT-PCR clearance, number of patients with improvement of pulmonary disease (CT imaging), number of patients experiencing disease progression, number of patients discharged from the hospital, and length of hospital stay.

***Measures of effect***

Risk ratio with 95% CIs for dichotomous outcomes and Standardised Mean Difference with 95% CI for continuous outcomes, measured at study endpoint. We will consider reporting hazard ratios (HR) when risk ratio not available.

**Data extraction (selection and coding)**

At least two review authors will independently screen titles and abstracts retrieved by the search strategy. Full-texts of potentially relevant studies will then be assessed independently by at least two authors and disagreements will be resolved through discussion with a third member of the review team.

We will use a data collection form to extract study characteristics and outcome data, which has been piloted on at least one study in the review. Two review authors will independently extract study characteristics and outcome data from included studies, as follows:

Methods: first author or acronym, year of publication, study design.

Participants: diagnosis, sample size, mean age, gender distribution, severity of illness, setting.

Interventions: number of patients allocated to each arm, drug name, dose, duration of the interventions and follow-up.

Outcomes: all-cause mortality, adverse events and serious adverse events.

Additional outcomes: Patients with SARS-CoV-2 nasal or pharyngeal swab RT-PCR clearance, time to nasal or pharyngeal swab RT-PCR clearance, number of patients with improvement of pulmonary disease (CT imaging), number of patients experiencing disease progression, number of patients discharged from the hospital, and length of hospital stay.

Notes: Country, funding source.

**Risk of bias (quality) assessment**

At least two review authors will independently assess the risk of bias of each study, using the criteria outlined in the Cochrane Handbook for Systematic Reviews of Interventions (Higgins et al., 2019). The following domains will be assessed: random sequence generation, allocation concealment, blinding of personnel and participants, blinding of outcome assessment, incomplete outcome data, and selective outcome reporting. We will judge each potential source of bias as high, low or unclear. We will report the ’Risk of bias’ judgements across different studies for each of the domains listed. Where information on risk of bias relates to unpublished data or correspondence with a trialist, we will note this in the ’Risk of bias’ table. A judgment of high risk of bias in one or more domain will be considered as a ‘high risk’ study, a judgment of low risk of bias in most of the domains will be considered as a ‘low risk’ study, and a judgment of unclear risk of bias in most of the domains as an ‘unclear risk’ study. When considering treatment effects, we will take into account the risk of bias for the studies that contribute to that outcome.

**Strategy for data synthesis**

Dichotomous outcomes will be analysed by calculating the relative risk (RR) for each trial with the uncertainty in each result being expressed by its 95% confidence interval (CI).

Continuous outcomes will be analysed by calculating the mean difference (MD) with the relative 95% CI when the study used the same instruments for assessing the outcome. We will use the standardised mean difference (SMD) when studies used different instruments.

We will consider reporting narratively hazard ratios (HR) when other measures are not available.

If dichotomous outcome data are missing, they will be managed according to the intention-to-treat (ITT) principle, and we will assume that patients who dropped out after randomisation had a negative outcome. Missing continuous outcome data will either be analysed using the last observation carried forward to the final assessment (LOCF) or, if LOCF data are reported by the trial authors, will be analysed on an endpoint basis, including only participants with a final assessment. When P values, t-values, CIs or standard errors are reported in articles, we will calculate SDs from their values as in Higgins et al (2011).

We will perform pairwise meta-analyses for primary and secondary outcomes using a random-effects model in RevMan for every treatment comparison (DerSimonian 1986).

We will perform network meta-analysis (NMA) for the primary outcome. NMA is a method of synthesising information from a network of trials addressing the same question but involving different interventions (Cipriani 2013). NMA combines direct evidence and indirect evidence across a network of randomised trials into a single effect size, and it can increase the precision in the estimates while randomisation is respected. We will perform NMA using a random-effects model within a frequentist setting assuming equal heterogeneity across all comparisons, and we will account for correlations induced by multi-arm studies. The models will enable us to estimate the probability of each intervention being the best, given the relative effect sizes as estimated in NMA. We will perform NMA in Stata 16 using the ’mvmeta’ command and Stata routines available at http://www.mtm.uoi.gr (Chaimani 2014; White 2011; White 2012).

Results of meta-analysis and NMA will be applied when reasonable and presented as summary relative effect sizes OR or SMD) for each possible pair of treatments.

We will use the GRADE approach to rating the certainty of the evidence.

The systematic review will be updated every month. As soon as new studies are included, their basic study characteristics are extracted and provided online. We will keep the living systematic review up to date for 2020.

**Analysis of subgroups or subsets**

We will run a subgroup analysis on disease severity for all-cause mortality.

**GRADE quality assessment of the comparisons in the network**

We applied the GRADE for the primary outcomes (All-cause mortality, adverse events and serious adverse events) by using the Confidence in Network Meta-Analysis Software (CINeMA). CINeMA is a software which uses the netmeta R-package for performing Network meta-analysis of the data. We assessed each network estimate according to the following criteria.

(1) Study limitation: In each direct comparison, we judged the risk of bias as low, moderate, or high considering the risk of bias assessment for the majority of the studies. We assigned numerical scores to these risk of bias judgments: 1 for low, 2 for moderate, and 3 for high risk of bias. Then, we derived the judgment for study limitations for each pairwise estimate as a weighted average of the risk of bias judgments from all direct estimates and the contribution of each direct estimate to the network estimates from the contributions matrix.

(2) Indirectness: We trust that the included studies in our review answered to the targeted research question in terms of populations, treatments, and outcomes. We have assured transitivity in our network by limiting the included studies to studies on pharmacological compounds. We further ran subgroup network meta-analyses for severity of disease and assured that they did not violate transitivity of the network.

(3) Inconsistency: We rated two concepts, heterogeneity and incoherence (as disagreement between direct and indirect estimates), in this domain. For heterogeneity in each pairwise estimates, we looked at the CIs for each pairwise network estimates and we checked whether the conclusions did not change between CIs and predictive intervals. If conclusions changed, we downgraded the comparison. We also compared the tau^2^ for pairwise comparisons with at least two studies and with the expected tau^2^ for pharma interventions and subjective outcome (following Turner et al. 2013). For incoherence in direct/mixed evidence, we compared the p-value from side-splitting test and design-by-treatment test.

(4) Imprecision: We considered a clinically meaningful threshold for OR to be 0.80 or 1.25 and downgraded the estimate if the OR point estimate was 1 or more and the lower limit of its CI was below 0.80; or if the OR point estimate was less than 1 and the upper limit of its CI was above 1.25.

(5) Publication bias: We looked at the comparison-adjusted funnel plot to check the presence of overall publication bias.

We derived the overall judgment of the certainty of evidence considering the domains altogether and downgraded the evidence by one if a domain was rated as “some concerns” and by two if a domain was rated as “major concerns”.

Finally, we assigned to each comparison an overall qualitative judgment based on four levels of certainty of evidence: high, moderate, low, very low.

### **Appendix 2. PRISMA NMA Checklist of Items to Include When Reporting A Systematic Review Involving a Network Meta-analysis.**

| **Section/Topic** | **Item #** | **Checklist Item** | **Reported on Section, Sub-section, Paragraph #** |
| --- | --- | --- | --- |
| **TITLE** |  |  |  |
| Title | 1 | Identify the report as a systematic review *incorporating a network meta-analysis (or related form of meta-analysis).* | **Title** |
|  |  |  |  |
| **ABSTRACT** |  |  |  |
| Structured summary | 2 | Provide a structured summary including, as applicable:  **Background:** main objectives  **Methods:** data sources; study eligibility criteria, participants, and interventions; study appraisal; and *synthesis methods, such as network meta-analysis.*  **Results:** number of studies and participants identified; summary estimates with corresponding confidence/credible intervals; *treatment rankings may also be discussed. Authors may choose to summarize pairwise comparisons against a chosen treatment included in their analyses for brevity.*  **Discussion/Conclusions:** limitations; conclusions and implications of findings.  **Other:** primary source of funding; systematic review registration number with registry name. | **Abstract, Background, paragraph 1**  **Abstract, Methods and Findings, paragraphs 1**  **Abstract, Methods and Findings, paragraphs 2, 3**  **Abstract, Conclusions, paragraph 1**  **Abstract, Methods and Findings, paragraph 1.**  **Fundings are in line 427-430** |
|  |  |  |  |
| **INTRODUCTION** |  |  |  |
| Rationale | 3 | Describe the rationale for the review in the context of what is already known*, including mention of why a network meta-analysis has been conducted.* | **Introduction, paragraphs 1, 2, 3, 4** |
| Objectives | 4 | Provide an explicit statement of questions being addressed, with reference to participants, interventions, comparisons, outcomes, and study design (PICOS). | **Introduction, paragraph 4** |
|  |  |  |  |
| **METHODS** |  |  |  |
| Protocol and registration | 5 | Indicate whether a review protocol exists and if and where it can be accessed (e.g., Web address); and, if available, provide registration information, including registration number. | **Methods, paragraphs 1, 2;**  **Appendix 1** |
| Eligibility criteria | 6 | Specify study characteristics (e.g., PICOS, length of follow-up) and report characteristics (e.g., years considered, language, publication status) used as criteria for eligibility, giving rationale. *Clearly describe eligible treatments included in the treatment network, and note whether any have been clustered or merged into the same node (with justification).* | **Methods, Search strategy and selection criteria, paragraphs 1, 2, 3** |
| Information sources | 7 | Describe all information sources (e.g., databases with dates of coverage, contact with study authors to identify additional studies) in the search and date last searched. | **Methods, Search strategy and selection criteria, paragraph 1; Appendix 1** |
| Search | 8 | Present full electronic search strategy for at least one database, including any limits used, such that it could be repeated. | **Appendix 1** |
| Study selection | 9 | State the process for selecting studies (i.e., screening, eligibility, included in systematic review, and, if applicable, included in the meta-analysis). | **Methods, Data extraction, paragraphs 1, 2** |
| Data collection process | 10 | Describe method of data extraction from reports (e.g., piloted forms, independently, in duplicate) and any processes for obtaining and confirming data from investigators. | **Methods, Data extraction, paragraph 1** |
| Data items | 11 | List and define all variables for which data were sought (e.g., PICOS, funding sources) and any assumptions and simplifications made. | **Methods, Data extraction, paragraphs 1, 2; Methods, Outcomes, paragraph 1; Methods, Dealing with missing data, paragraph 1** |
| **Geometry of the network** | **S1** | Describe methods used to explore the geometry of the treatment network under study and potential biases related to it. This should include how the evidence base has been graphically summarized for presentation, and what characteristics were compiled and used to describe the evidence base to readers. | **Methods, Data analysis, paragraph 2** |
| Risk of bias within individual studies | 12 | Describe methods used for assessing risk of bias of individual studies (including specification of whether this was done at the study or outcome level), and how this information is to be used in any data synthesis. | **Methods, Data extraction, paragraph 2** |
| Summary measures | 13 | State the principal summary measures (e.g., risk ratio, difference in means). *Also describe the use of additional summary measures assessed, such as treatment rankings and surface under the cumulative ranking curve (SUCRA) values, as well as modified approaches used to present summary findings from meta-analyses.* | **Methods, Data analysis, paragraphs 1, 2, 5** |
| Planned methods of analysis | 14 | Describe the methods of handling data and combining results of studies for each network meta-analysis. This should include, but not be limited to:   - *Handling of multi-arm trials;* - *Selection of variance structure;* - *Selection of prior distributions in Bayesian analyses; and* - *Assessment of model fit.* | **Methods, Data analysis, paragraphs 1, 2, 3; PROSPERO (CRD42017042900)** |
| **Assessment of Inconsistency** | **S2** | Describe the statistical methods used to evaluate the agreement of direct and indirect evidence in the treatment network(s) studied. Describe efforts taken to address its presence when found. | **Methods, Data analysis, paragraph 3** |
| Risk of bias across studies | 15 | Specify any assessment of risk of bias that may affect the cumulative evidence (e.g., publication bias, selective reporting within studies). | **Methods, Data extraction, paragraph 2; Methods, Data analysis, paragraph 4** |
| Additional analyses | 16 | Describe methods of additional analyses if done, indicating which were pre-specified. This may include, but not be limited to, the following:   - Sensitivity or subgroup analyses; - Meta-regression analyses; - *Alternative formulations of the treatment network; and* - *Use of alternative prior distributions for Bayesian analyses (if applicable).* | **Methods, Data analysis, paragraph 6** |
|  |  |  |  |
| **RESULTS†** |  |  |  |
| Study selection | 17 | Give numbers of studies screened, assessed for eligibility, and included in the review, with reasons for exclusions at each stage, ideally with a flow diagram. | **Results, paragraph 1; Figure 1** |
| **Presentation of network structure** | **S3** | Provide a network graph of the included studies to enable visualization of the geometry of the treatment network. | **Figure 2; Appendix 7** |
| **Summary of network geometry** | **S4** | Provide a brief overview of characteristics of the treatment network. This may include commentary on the abundance of trials and randomized patients for the different interventions and pairwise comparisons in the network, gaps of evidence in the treatment network, and potential biases reflected by the network structure. | **Results, paragraphs 1, 2** |
| Study characteristics | 18 | For each study, present characteristics for which data were extracted (e.g., study size, PICOS, follow-up period) and provide the citations. | **Table 1; Appendix 4** |
| Risk of bias within studies | 19 | Present data on risk of bias of each study and, if available, any outcome level assessment. | **Results, Study characteristics, paragraph 1; Appendix 5** |
| Results of individual studies | 20 | For all outcomes considered (benefits or harms), present, for each study: 1) simple summary data for each intervention group, and 2) effect estimates and confidence intervals. *Modified approaches may be needed to deal with information from larger networks.* | **Appendix 4; Full data extraction file (available upon request to the corresponding author)** |
| Synthesis of results | 21 | Present results of each meta-analysis done, including confidence/credible intervals. *In larger networks, authors may focus on comparisons versus a particular comparator (e.g. placebo or standard care), with full findings presented in an appendix. League tables and forest plots may be considered to summarize pairwise comparisons.* If additional summary measures were explored (such as treatment rankings), these should also be presented. | **Results, Pairwise meta-analysis, paragraphs 1, 2; Results, Network meta-analysis, paragraph 1; Figures 3, 4; Appendices 6, 9, 10, 11** |
| **Exploration for inconsistency** | **S5** | Describe results from investigations of inconsistency. This may include such information as measures of model fit to compare consistency and inconsistency models, *P* values from statistical tests, or summary of inconsistency estimates from different parts of the treatment network. | **Results, Network meta-analysis, paragraph 1; Appendices 8, 11** |
| Risk of bias across studies | 22 | Present results of any assessment of risk of bias across studies for the evidence base being studied. | **Results, Network meta-analysis, paragraph 1; Appendices 9, 11** |
| Results of additional analyses | 23 | Give results of additional analyses, if done (e.g., sensitivity or subgroup analyses, meta-regression analyses*, alternative network geometries studied, alternative choice of prior distributions for Bayesian analyses,* and so forth). | **Results, Subgroup analysis, paragraph 1; Figure 4** |
|  |  |  |  |
| **DISCUSSION** |  |  |  |
| Summary of evidence | 24 | Summarize the main findings, including the strength of evidence for each main outcome; consider their relevance to key groups (e.g., healthcare providers, users, and policy-makers). | **Discussion, paragraphs 1, 2, 3, 4** |
| Limitations | 25 | Discuss limitations at study and outcome level (e.g., risk of bias), and at review level (e.g., incomplete retrieval of identified research, reporting bias). *Comment on the validity of the assumptions, such as transitivity and consistency. Comment on any concerns regarding network geometry (e.g., avoidance of certain comparisons).* | **Discussion, paragraphs 6, 7, 8, 9, 10** |
| Conclusions | 26 | Provide a general interpretation of the results in the context of other evidence, and implications for future research. | **Discussion, paragraphs 5, 11, 12** |
|  |  |  |  |
| **FUNDING** |  |  |  |
| Funding | 27 | Describe sources of funding for the systematic review and other support (e.g., supply of data); role of funders for the systematic review. This should also include information regarding whether funding has been received from manufacturers of treatments in the network and/or whether some of the authors are content experts with professional conflicts of interest that could affect use of treatments in the network. | **Funding; Conflict of interest; Acknowledgments** |

### **Appendix 3. References for included trials**

1. **Abbaspour Kasgari 2020**

- Abbaspour Kasgari, H., Moradi, S., Shabani, A.M., Babamahmoodi, F., Davoudi Badabi, A.R., Davoudi, L., et al. (2020). Evaluation of the efficacy of sofosbuvir plus daclatasvir in combination with ribavirin for hospitalized COVID-19 patients with moderate disease compared with standard care: a single-centre, randomized controlled trial. J Antimicrob Chemother. 1;75(11):3373-3378. doi: 10.1093/jac/dkaa332

1. **Abd-Elsalam 2020a**

- Abd-Elsalam, S., Esmail, E.S., Khalaf, M., Abdo, E.F., Medhat, M.A., Abd El Ghafar, M.S., et al. (2020). Hydroxychloroquine in the Treatment of COVID-19: A Multicenter Randomized Controlled Study. Am J Trop Med Hyg. 103(4):1635-9.

1. **Abd-Elsalam 2020b**

- Abd-Elsalam, S., Soliman, S., Esmail, E.S., Khalaf, M., Mostafa, E.F., Medhat, M.A., et al. (2020). Do Zinc Supplements Enhance the Clinical Efficacy of Hydroxychloroquine? a Randomized, Multicenter Trial. Biol Trace Elem Res. Epub ahead of print. doi: 10.1007/s12011-020-02512-1.

1. **Agarwal 2020**

- *Agarwal, A., Mukherjee, A., Kumar, G., Chatterjee, P., Bhatnagar, T., Malhotra, P.; PLACID Trial Collaborators. (2020). Convalescent plasma in the management of moderate covid-19 in adults in India: open label phase II multicentre randomised controlled trial (PLACID Trial). BMJ. 371:m3939. doi: 10.1136/bmj.m3939. Erratum in: BMJ. 2020 Nov 3;371:m4232.
- Agarwal, A., Mukherjee, A., Kumar, G., Chatterjee, P., Bhatnagar, T., Malhotra, P., et al. Convalescent plasma in the management of moderate COVID-19 in India: An open-label parallel-arm phase II multicentre randomized controlled trial (PLACID Trial). [Preprint]

Available at: <https://www.medrxiv.org/content/10.1101/2020.09.03.20187252v2>.

1. **AlQahtani 2020**

- AlQahtani, M, Abdulrahman, A, AlMadani, A, AlAli, SY, Al Zamrooni, AM, Hejab, A, et al. Randomized controlled trial of convalescent plasma therapy against standard therapy in patients with severe COVID-19 disease. [Preprint]

Available at: https://www.medrxiv.org/content/10.1101/2020.11.02.20224303v1.

1. **Ansarin 2020**

- Ansarin, K., Tolouian, R., Ardalan, M., Taghizadieh, A., Varshochi, M., Teimouri, S., et al. (2020). Effect of bromhexine on clinical outcomes and mortality in COVID-19 patients: A randomized clinical trial. Bioimpacts. 10(4):209-215. doi: 10.34172/bi.2020.27. Epub 2020 Jul 19. PMID: 32983936

1. **Avendaño-Solà 2020**

- Avendano-Sola, C., Ramos-Martinez, A., Munez-Rubio, E., Ruiz-Antoran, B., Malo de Molina, R., Torres, F., et al. (2020). Convalescent Plasma for COVID-19: A multicenter, randomized clinical trial. [Preprint] Available at: https://www.medrxiv.org/content/10.1101/2020.08.26.20182444v3

1. **Bajpal 2020**

- Bajpai, M., Kumar, S., Maheshwari, A., Chhabra, K., Kale, P., Gupta, A., et al. (2020). Efficacy of Convalescent Plasma Therapy compared to Fresh Frozen Plasma in Severely ill COVID-19 Patients: A Pilot Randomized Controlled Trial. [Preprint]

Available at: https://www.medrxiv.org/content/10.1101/2020.10.25.20219337v1

1. **Beigel 2020**

- *Beigel, J.H., Tomashek, K.M., Dodd, L.E., Mehta, A.K., Zingman, B.S., Kalil, A.C., et al.; ACTT-1 Study Group Members. (2020). Remdesivir for the Treatment of Covid-19 - Final Report. N Engl J Med. 383(19):1813-1826. doi: 10.1056/NEJMoa2007764. Epub 2020 Oct 8.
- Beigel, J.H., Tomashek, K.M., Dodd, L.E., Mehta, A.K., Zingman, B.S., Kalil, A.C., et al. (2020). Remdesivir for the Treatment of Covid-19. — Preliminary Report. N Engl J Med. doi:10.1056/NEJMoa2007764.

1. **Brown 2020**

- Brown, S.M., Peltan, I.D., Webb, B., Kumar, N., Starr, N., Grissom, C., et al. (2020).Hydroxychloroquine versus Azithromycin for Hospitalized Patients with Suspected or Confirmed COVID-19 (HAHPS). Protocol for a Pragmatic, Open-Label, Active Comparator Trial. Ann Am Thorac Soc. 17(8):1008-1015. doi: 10.1513/AnnalsATS.202004-309SD.

1. **Cao B 2020**

- Cao, B., Wang, Y., Wen, D., Liu, W., Wang, J., Fan, G., et al. (2020). A Trial of Lopinavir-Ritonavir in Adults Hospitalized with Severe Covid-19. N Engl J Med. 382(19):1787-1799. doi: 10.1056/NEJMoa2001282. Epub 2020 Mar 18.

1. **Cao Y 2020**

- Cao, Y., Wei, J., Zou, L., Jiang, T., Wang, G., Chen, L., et al. (2020). Ruxolitinib in treatment of severe coronavirus disease 2019 (COVID-19): A multicenter, single-blind, randomized controlled trial. J Allergy Clin Immunol. 146(1):137-146.e3. doi: 10.1016/j.jaci.2020.05.019. Epub 2020 May 26.

1. **Cavalcanti 2020**

- Cavalcanti, A.B., Zampieri, F.G., Rosa, R.G., Azevedo, L.C.P., Veiga, V.C., Avezum, A., et al; Coalition Covid-19 Brazil I Investigators. (2020). Hydroxychloroquine with or without Azithromycin in Mild-to-Moderate Covid-19. N Engl J Med. Epub ahead of print. doi: 10.1056/NEJMoa2019014.

1. **Chen C 2020**

- Chen, C., Zhang, Y., Huang, J., Yin, P., Cheng, Z., Wu, J., et al. (2020). Favipiravir versus Arbidol for COVID-19: A Randomized Clinical Trial. [Preprint]

Available at: https://www.medrxiv.org/content/10.1101/2020.03.17.20037432v4

1. **Chen C-P 2020**

- Chen, C.P., Lin, Y.C., Chen, T-C., Tseng, T-Y., Wong, H-L., Kuo, C-Y., et al. (2020). A Multicenter, randomized, open-label, controlled trial to evaluate the efficacy and tolerability of hydroxychloroquine and a retrospective study in adult patients with mild to moderate Coronavirus disease 2019 (COVID-19). [Preprint]

Available at: https://www.medrxiv.org/content/10.1101/2020.07.08.20148841v1.

1. **Chen J 2020a**

- Chen, J., Liu, D., Liu, L., Liu, P., Xu, Q., Xia, L., et al. (2020). [A pilot study of hydroxychloroquine in treatment of patients with moderate COVID-19]. Zhejiang Da Xue Xue Bao Yi Xue Ban. 49(2):215-219. Chinese. doi: 10.3785/j.issn.1008-9292.2020.03.03.

1. **Chen J 2020b**

- Chen, J., Xia, L., Liu, L., Xu, Q., Ling, Y., Huang, D., et al. (2020). Antiviral Activity and Safety of Darunavir/Cobicistat for the Treatment of COVID-19. Open Forum Infect Dis. 7(7):ofaa241. doi: 10.1093/ofid/ofaa241.

1. **Chen L 2020**

- Chen, L., Zhang, Z-Y., Fu, J-G., Feng, Z-P., Zhang, S-Z., Han, Q-Y., et al. (2020). Efficacy and safety of chloroquine or hydroxychloroquine in moderate type of COVID-19: a prospective open-label randomized controlled study. [Preprint]

Available at: https://www.medrxiv.org/content/10.1101/2020.06.19.20136093v1

1. **Chen P 2020**

- Chen, P., Nirula, A., Heller, B., Gottlieb, R.L., Boscia, J., Morris J., et al; BLAZE-1 Investigators. (2020). SARS-CoV-2 Neutralizing Antibody LY-CoV555 in Outpatients with Covid-19. N Engl J Med. Epub ahead of print. doi: 10.1056/NEJMoa2029849.

1. **Chen Z 2020**

- Chen, Z., Hu, J., Zhang, Z., Jiang, S., Han, S., Yan, D., et al. (2020). Efficacy of hydroxychloroquine in patients with COVID-19: results of a randomized clinical trial. [Preprint]

Available at: https://www.medrxiv.org/content/10.1101/2020.03.22.20040758v3

1. **Cheng 2020**

- Cheng, L.L., Guan, W.J., Duan, C. Y., Zhang, N. F., Lei, C. L., Hu, Y., et al. (2020). Effect of Recombinant Human Granulocyte Colony-Stimulating Factor for Patients With Coronavirus Disease 2019 (COVID-19) and Lymphopenia: A Randomized Clinical Trial. JAMA internal medicine, e205503. Advance online publication. https://doi.org/10.1001/jamainternmed.2020.5503

1. **Chowdurry 2020**

- Chowdhury, A., Shahbaz, M., Karim, M., Islam, J., Guo, D., He, S. (2020). A Randomized Trial of Ivermectin-Doxycycline and Hydroxychloroquine-Azithromycin therapy on COVID19 patients. [Preprint] Available at: https://assets.researchsquare.com/files/rs-38896/v1/3ee350c3-9d3f-4253-85f9-1f17f3af9551.pdf

1. **Corral L 2020**

- Corral, L., Bahamonde, A., Arnaiz delas Revillas, F., Gomez-Barquero, J., Abadia-Otero, J., Garcia-Ibarbia, C., et al. (2020). GLUCOCOVID: A controlled trial of methylprednisolone in adults hospitalized with COVID-19 pneumonia. [Preprint]

Available at: https://www.medrxiv.org/content/10.1101/2020.06.17.20133579v1

1. **Dabbous 2020**

- Dabbous, H.M., El-Sayed, M.H., Assal, G.E., et al. (2020). A Randomized Controlled Study Of Favipiravir Vs Hydroxychloroquine In COVID-19 Management: What Have We Learned So Far?. Research Square; 2020. DOI: 10.21203/rs.3.rs-83677/v1.

1. **Davoudi-Monfared E 2020**

- *Davoudi-Monfared, E., Rahmani, H., Khalili, H., Hajiabdolbaghi, M., Salehi, M., Abbasian, L., et al. (2020). Efficacy and safety of interferon β-1a in treatment of severe COVID-19: A randomized clinical trial. Antimicrob Agents Chemother. [published online ahead of print, 2020 Jul 13] doi:10.1128/AAC.01061-20.
- Davoudi-Monfared, E., Rahmani, H., Khalili, H., Hajiabdolbaghi, M., Salehi, M., Abbasian, L., et al. (2020). Efficacy and safety of interferon β-1a in treatment of severe COVID-19: A randomized clinical trial AAC Accepted Manuscript Posted Online 13 July 2020 Antimicrob. Agents Chemother. doi:10.1128/AAC.01061-20
- Davoudi-Monfared, E., Rahmani, H., Khalili, H., Hajiabdolbaghi, M., Salehi, M., Abbasian, L., et al. (2020). Efficacy and safety of interferon β-1a in treatment of severe COVID-19: A randomized clinical trial. [Preprint]

Available at: https://www.medrxiv.org/content/10.1101/2020.05.28.20116467v1

1. **Deftereos 2020**

- Deftereos, S.G., Giannopoulos, G., Vrachatis, D.A., Siasos, G.D., Giotaki, S.G., Gargalianos, P., et al; GRECCO-19 investigators. (2020). Effect of Colchicine vs Standard Care on Cardiac and Inflammatory Biomarkers and Clinical Outcomes in Patients Hospitalized with Coronavirus Disease 2019: The GRECCO-19 Randomized Clinical Trial. JAMA Netw Open. 3(6):e2013136. doi: 10.1001/jamanetworkopen.2020.13136

1. **Dequin 2020**

- Dequin, P.F., Heming, N., Meziani, F., Plantefève, G., Voiriot, G., Badié, J., et al. (2020). Effect of Hydrocortisone on 21-Day Mortality or Respiratory Support Among Critically Ill Patients With COVID-19: A Randomized Clinical Trial. [published online ahead of print, 2020 Sep 2]. JAMA. 2020;10.1001/jama.2020.16761.

1. **Duarte 2020**

- Duarte, M., Pelorosso, F.G., Nicolosi, L., Salgado, M.V., Vetulli, H., Aquieri, A., et al. (2020). Telmisartan for treatment of Covid-19 patients: an open randomized clinical trial. Preliminary report. [Preprint] Available at: https://www.medrxiv.org/content/10.1101/2020.08.04.20167205v2

1. **Dubèe 2020**

- Dubée, V., Roy, P-M., Vielle, B., Parot-Schinkel, E., Blanchet, O., Darsonval, A., et al. (2020). A placebo-controlled double blind trial of hydroxychloroquine in mild-to-moderate COVID-19. [Preprint] Available at: https://www.medrxiv.org/content/10.1101/2020.10.19.20214940v1

1. **Edalatifard 2020**

- Edalatifard, M., Akhtari, M., Salehi, M., Naderi, Z., Jamshidi, A., Mostafaei, S., et al. (2020). Intravenous methylprednisolone pulse as a treatment for hospitalised severe COVID-19 patients: results from a randomised controlled clinical trial. Eur Respir J. Epub ahead of print. doi:10.1183/13993003.02808-2020.

1. **Entrenas Castillo 2020**

- Entrenas Castillo, M., Entrenas Costa, L.M., Vaquero Barrios, J.M., Alcalá Díaz, J.F., López Miranda, J., Bouillon, R., et al. (2020). Effect of calcifediol treatment and best available therapy versus best available therapy on intensive care unit admission and mortality among patients hospitalized for COVID-19: A pilot randomized clinical study. J Steroid Biochem Mol Biol. 203, 105751. <https://doi.org/10.1016/j.jsbmb.2020.105751>

1. **Esquivel-Moynelo 2020**

- Esquivel-Moynelo, I., Perez-Escribano, J., Duncan-Robert, Y., Vazquez-Blonquist, D., Bequet-Romero, M., Baez-Rodríguez, L., et al. (2020). Effect and safety of combination of interferon alpha-2b and gamma or interferon alpha-2b for negativization of SARS-CoV-2 viral RNA. Preliminary results of a randomized controlled clinical trial. [Preprint]

Available at: https://www.medrxiv.org/content/10.1101/2020.07.29.20164251v2

1. **Furtado 2020**

- Furtado, R.H.M., Berwanger, O., Fonseca, H.A., Corrêa, T.D., Ferraz, L.R., Lapa, M.G., et al. Azithromycin in addition to standard of care versus standard of care alone in the treatment of patients admitted to the hospital with severe COVID-19 in Brazil (COALITION II): a randomised clinical trial. The Lancet. 396, 959 - 967

1. **Gharbharan A 2020**

- Gharbharan, A., Jordans, C.E., Geurtsvan, K., den Hollander, J., Karim, F., Mollema, F.P., et al. (2020). Convalescent Plasma for COVID-19. A randomized clinical trial. [Preprint].

Available at: https://www.medrxiv.org/content/10.1101/2020.07.01.20139857v1

1. **Gharebaghi 2020**

- Gharebaghi, N., Nejadrahim, R., Mousavi, S.J,, Sadat-Ebrahimi, S.R., Hajizadeh, R., (2020). The use of intravenous immunoglobulin gamma for the treatment of severe coronavirus disease 2019: a randomized placebo-controlled double-blind clinical trial. BMC Infect Dis. 20(1):786. doi: 10.1186/s12879-020-05507-4. Erratum in: BMC Infect Dis. 2020 Nov 26;20(1):895.

1. **Guvenmez 2020**

- Guvenmez, O., Keskin, H., Ay, B., Birinci, S., Kanca, M.F. (2020). The comparison of the effectiveness of lincocin® and azitro® in the treatment of covid-19-associated pneumonia: A prospective study. J Popul Ther Clin Pharmacol. 27(S Pt 1):e5-e10. doi: 10.15586/jptcp.v27iSP1.684.

1. **Hashim 2020**

- Hashim, H.A., Maulood, M.F., Rasheed, A.M., Fatak, D.F., Kabah, K.K., Abdulamir, A.S. (2020). Controlled randomized clinical trial on using Ivermectin with Doxycycline for treating COVID-19 patients in Baghdad, Iraq. [Preprint].

Available at: https://www.medrxiv.org/content/10.1101/2020.10.26.20219345v1

1. **Hermine 2020**

- Hermine, O., Mariette, X., Tharaux, P.L., Resche-Rigon, M., Porcher, R., Ravaud, P. et al. (2020). CORIMUNO-19 Collaborative Group. Effect of Tocilizumab vs Usual Care in Adults Hospitalized With COVID-19 and Moderate or Severe Pneumonia: A Randomized Clinical Trial. JAMA Intern Med. Epub ahead of print. doi: 10.1001/jamainternmed.2020.6820.

1. **Huang M 2020**

- Huang, M., Tang, T., Pang, P., Li, M., Ma, R., Lu, J., et al. (2020). Treating COVID-19 with Chloroquine. J Mol Cell Biol. 12(4):322-25 doi: https://doi.org/10.1093/jmcb/mjaa014..

1. **Huang Y-Q 2020**

- Huang, Y.Q., Tang, S.Q., Xu, X.L., Zeng, Y.M., H,e X.Q., Li, Y., et al. (2020). No Statistically Apparent Difference in Antiviral Effectiveness Observed Among Ribavirin Plus Interferon-Alpha, Lopinavir/Ritonavir Plus Interferon-Alpha, and Ribavirin Plus Lopinavir/Ritonavir Plus Interferon-Alpha in Patients With Mild to Moderate Coronavirus Disease 2019: Results of a Randomized, Open-Labeled Prospective Study. Front Pharmacol. 11:1071. doi: 10.3389/fphar.2020.01071.

1. **Hung Ivan Fang-Ngai 2020,**

- Hung, I.F., Lung, K.C., Tso, E.Y., Liu, R., Chung, T.W., Chu, M.Y., et al. (2020). Triple combination of interferon beta-1b, lopinavir-ritonavir, and ribavirin in the treatment of patients admitted to hospital with COVID-19: an open-label, randomised, phase 2 trial. Lancet. 395(10238):1695-1704. doi: 10.1016/S0140-6736(20)31042-4. Epub 2020 May 10.

1. **Ivashchenko 2020**

- Ivashchenko, A.A., Dmitriev, K.A., Vostokova, N.V., Azarova, V.N., Blinow, A.A., Egorova, A.N., et al. (2020). AVIFAVIR for Treatment of Patients with Moderate COVID-19: Interim Results of a Phase II/III Multicenter Randomized Clinical Trial. Clin Infect Dis. Advance online publication. https://doi.org/10.1093/cid/ciaa1176

1. **Jagannathan 2020**

- Jagannathan, P., Andrews, J.R., Bonilla, H., Hedlin, H., Jacobson, K.B., Balasubramanian, V., et al. (2020). Peginterferon Lambda-1a for treatment of outpatients with uncomplicated COVID-19: a randomized placebo-controlled trial. [Preprint].

Available at: https://www.medrxiv.org/content/10.1101/2020.11.18.20234161v1

1. **Jeronimo 2020**

- Jeronimo, C.M.P., Farias, M.E.L., Val, F.F.A., Sampaio, V.S., Alexandre, M.A.A., Melo, G.C., et al, for the Metcovid Team. (2020). Methylprednisolone as Adjunctive Therapy for Patients Hospitalized With COVID-19 (Metcovid): A Randomised, Double-Blind, Phase IIb, Placebo-Controlled Trial. Clin Infect Dis. Epub ahead of print. doi: 10.1093/cid/ciaa1177.

1. **Kamran 2020**

- Kamran, S.M., Mirza, Z-e-H., Naseem, A., Azam, R., Ullah, N., Saeed, F., et al. Clearing the fog: Is Hydroxychloroquine effective in reducing Corona virus disease-2019 progression: A randomized controlled trial. [Preprint].

Available at: https://www.medrxiv.org/content/10.1101/2020.07.30.20165365v2

1. **Khamis 2020**

- Khamis, F., Al Naabi, H., Al Lawati, A., Ambusaidi, Z., Al Sharji, M., Al Barwani, U., et al. (2020). Randomized Controlled Open Label Trial on the Use of Favipiravir Combined with Inhaled Interferon beta-1b in Hospitalized Patients with Moderate to Severe COVID-19 Pneumonia. Int J Infect Dis. S1201-9712(20)32319-5. Epub ahead of print. doi: 10.1016/j.ijid.2020.11.008.

1. **Krolewiecki 2020**

- Krolewiecki, A.J., Lifschitz, A., Moragas, M., Travacio, M., Valentini, R., Alonso, D.F., et al. (2020). Antiviral Effect of High-Dose Ivermectin in Adults with COVID-19: A Pilot Randomised, Controlled, Open Label, Multicentre Trial. Available at SSRN: https://ssrn.com/abstract=3714649 or <http://dx.doi.org/10.2139/ssrn.3714649>

1. **Kumar 2020**

- Kumar, S., de Souza, R., Nadkar, M., Guleria, R., Trikha, A., Joshi, S.R., et al. (2020). A two-arm, randomized, controlled, multi-centric, open-label Phase-2 study to evaluate the efficacy and safety of Itolizumab in moderate to severe ARDS patients due to COVID-19. [Preprint].

Available at: https://www.medrxiv.org/content/10.1101/2020.12.01.20239574v1

1. **Lenze 2020**

- Lenze, E.J., Mattar, C., Zorumski, C.F., Stevens, A., Schweiger, J., Nicol, G.E., et al. (2020). Fluvoxamine vs Placebo and Clinical Deterioration in Outpatients with Symptomatic COVID-19: A Randomized Clinical Trial. JAMA. Epub ahead of print. doi: 10.1001/jama.2020.22760.

1. **Li L 2020**

- Li, L., Zhang, W., Hu, Y., Tong, X., Zheng, S., Yang, J., et al. (2020). Effect of Convalescent Plasma Therapy on Time to Clinical Improvement in Patients With Severe and Life-threatening COVID-19: A Randomized Clinical Trial. JAMA. doi: 10.1001/jama.2020.10044. Erratum in: JAMA. 2020 Aug 4;324(5):519.

1. **Li T 2020**

- Li, T., Sun, L., Zhang, W., Zheng, C., Jiang, C., Chen, M., et al. Bromhexine Hydrochloride Tablets for the Treatment of Moderate COVID-19: An Open-Label Randomized Controlled Pilot Study. Clin Transl Sci. Epub ahead of print. doi: 10.1111/cts.12881.

1. **Li Y 2020**

- *Li, Y., Xie, Z., Lin, W., Cai, W., Wen, C., Guan, Y., et al. (2020). Efficacy and Safety of Lopinavir/Ritonavir or Arbidol in Adult Patients with Mild/Moderate COVID-19: An Exploratory Randomized Controlled Trial. Med (N Y). Epub ahead of print. doi: 10.1016/j.medj.2020.04.001.
- Li, Y., Xie, Z., Lin, W., Cai, W., Wen, C., Guan, Y., et al. (2020). An exploratory randomized, controlled study on the efficacy and safety of lopinavir/ritonavir or arbidol treating adult patients hospitalized with mild/moderate COVID-19 (ELACOI). [Preprint].

Available at: https://www.medrxiv.org/content/10.1101/2020.03.19.20038984v2

1. **Lopes 2020**

- Lopes, M.I.F., Bonjorno, L.P., Giannini, M.C., Amaral, N.B., Benatti, M.N., Rezek, U.C., et al. (2020). Beneficial effects of colchicine for moderate to severe COVID-19: an interim analysis of a randomized, double-blinded, placebo controlled clinical trial. [Preprint].

Available at: https://www.medrxiv.org/content/10.1101/2020.08.06.20169573v2.

1. **Lou 2020**

- Lou, Y., Liu, L., Yao, H., Hu, X., Su J., Xu, K., et al. (2020). Clinical Outcomes and Plasma Concentrations of Baloxavir Marboxil and Favipiravir in COVID-19 Patients: An Exploratory Randomized, Controlled Trial. [Preprint]

Available at: <https://www.medrxiv.org/content/10.1101/2020.04.29.20085761v1>

1. **Maldonado 2020**

- Maldonado, V., Hernandez-Ramírez, C., Oliva-Pérez, E.A., Sánchez-Martínez, C.O., Pimentel-González, J.F., Molina-Sánchez, J.R., et al. (2020). Pentoxifylline decreases serum LDH levels and increases lymphocyte count in COVID-19 patients: Results from an external pilot study. Int Immunopharmacol. 2021;90:107209.

1. **Mansour 2020**

- Mansour, E., Palma, A.C., Ulaf, R.G., Ribeiro, L.C., Bernardes, A.F., Nunes, T.A., et al. (2020). Pharmacological inhibition of the kinin-kallikrein system in severe COVID-19 A proof-of-concept study. [Preprint].

Available at: https://www.medrxiv.org/content/10.1101/2020.08.11.20167353v1

1. **Mehboob 2020**

- Mehboob, R., Ahmad, F., Qayyum, A., Rana, M.A., Gilani, S.A., Tariq, M.A., et al. (2020). Aprepitant as a combinant with Dexamethasone reduces the inflammation via Neurokinin 1 Receptor Antagonism in severe to critical Covid-19 patients and potentiates respiratory recovery: A novel therapeutic approach. [Preprint].
- Available at: https://www.medrxiv.org/content/10.1101/2020.08.01.20166678v3.

1. **Miller 2020**

- Miller, J., Bruen, C., Schnaus M., Zhang, J., Ali, S., Lind, A., et al. (2020). Auxora versus standard of care for the treatment of severe or critical COVID-19 pneumonia: results from a randomized controlled trial. Crit Care. 24(1), 502. doi: 10.1186/s13054-020-03220-x.

1. **Mitijà 2020**

- Mitjà, O., Corbacho-Monné, M., Ubals, M., Tebe, C., Peñafiel, J., Tobias, A., et al; (2020). BCN PEP-CoV-2 RESEARCH GROUP. Hydroxychloroquine for Early Treatment of Adults with Mild Covid-19: A Randomized-Controlled Trial. Clin Infect Dis. Epub ahead of print. doi: 10.1093/cid/ciaa1009.

1. **Monk 2020**

- Monk, P.D., Marsden, R.J., Tear, V.J., Brookes, J., Batten, T.N., Mankowski M, et al; Inhaled Interferon Beta COVID-19 Study Group. Safety and efficacy of inhaled nebulised interferon beta-1a (SNG001) for treatment of SARS-CoV-2 infection: a randomised, double-blind, placebo-controlled, phase 2 trial. Lancet Respir Med. 2020 Nov 12:S2213-2600(20)30511-7. doi: 10.1016/S2213-2600(20)30511-7. Epub ahead of print.

1. **Morteza 2020**

- Morteza, S.N., Nematollah, G., Peyman, N., Abbas, A., Leila, Z., Amir, J., et al. (2020). Ivermectin as an adjunct treatment for hospitalized adult COVID-19 patients: A randomized multi-center clinical trial.. [Preprint]. Available at: Research Square https://doi.org/10.21203/rs.3.rs-109670/v1

1. **Nojomi 2020**

- Nojomi, M., Yasin, Z., Keyvani, H., Makiani, M.J., Roham, M., Laali, A., et al. (2020). Effect of Arbidol on COVID-19: A Randomized Controlled Trial. [Preprint]. Available at: Research Square https://doi.org/10.21203/rs.3.rs-78316/v1

1. **Omrani 2020**

- Omrani, A.S., Pathan, S.A., Thomas, S.A., Harris, T.R.E., Coyle, P.V., Thomas, C.E., et al. (2020). Randomized double-blinded placebo-controlled trial of hydroxychloroquine with or without azithromycin for virologic cure of non-severe Covid-19. E Clinical Medicine. 100645. doi: 10.1016/j.eclinm.2020.100645. Epub 2020 Nov 20.

1. **Pan (SOLIDARITY trial) 2020**

- *WHO Solidarity Trial Consortium, Pan, H., Peto, R., Henao-Restrepo, A.M., Preziosi, M.P., Sathiyamoorthy, V., Abdool Karim, Q., et al. (2020). Repurposed Antiviral Drugs for Covid-19 - Interim WHO Solidarity Trial Results. N Engl J Med. 2020 Dec 2. doi: 10.1056/NEJMoa2023184. Epub ahead of print.
- WHO Solidarity Trial Consortium, Pan H, Peto R, Karim QA, Alejandria M, Henao-Restrepo AM, García CH, et al. Repurposed antiviral drugs for COVID-19 –interim WHO SOLIDARITY trial results. [Preprint].

Available at: https://www.medrxiv.org/content/10.1101/2020.10.15.20209817v1.

1. **Rahmani 2020**

- Rahmani, H., Davoudi-Monfared, E., Nourian, A., Khalili, H., Hajizadeh, N., Jalalabadi, N.Z., et al. (2020). Interferon β-1b in treatment of severe COVID-19: A randomized clinical trial. Int Immunopharmacol. 88:106903. doi: 10.1016/j.intimp.2020.106903. Epub 2020 Aug 24.

1. **Ray 2020**

- Ray, Y., Paul, S.R., Bandopadhyay, P., D’Rozario, R., Sarif, J., Lahiri, A., et al. (2020). Clinical and immunological benefits of convalescent plasma therapy in severe COVID-19: insights from a single center open label randomised control trial. [Preprint].

Available at: https://www.medrxiv.org/content/10.1101/2020.11.25.20237883v1

1. **Recovery Trial**

- *Horby, P., Mafham, M., Linsell, L., Bell, J.L., Staplin, N., Emberson, J.R., et al. (2020). Effect of Hydroxychloroquine in Hospitalized Patients with COVID-19: Preliminary results from a multi-centre, randomized, controlled trial. [Preprint].

Available at: https://www.medrxiv.org/content/10.1101/2020.07.15.20151852v1

- RECOVERY Collaborative Group, Horby, P., Lim, W.S., et al Dexamethasone in Hospitalized Patients with Covid-19 — Preliminary Report. (2020). N Engl J Med. Available from: <https://www.nejm.org/doi/full/10.1056/NEJMoa2021436>
- *Horby, P., Lim, WS., Emberson, J., Mafham, M., Bell, J., Linsell, L., et al. (2020). Effect of Dexamethasone in Hospitalized Patients with COVID-19: Preliminary Report. [Preprint].

Available at: <https://www.medrxiv.org/content/10.1101/2020.06.22.20137273v1>

- *RECOVERY Collaborative Group. (2020). Lopinavir-ritonavir in patients admitted to hospital with COVID-19 (RECOVERY): a randomised, controlled, open-label, platform trial. Lancet. 396(10259):1345–52. Epub ahead of print. doi: 10.1016/S0140-6736(20)32013-4.

1. **REMAP-CAP trial**

- Angus, D.C., Derde, L., Al-Beidh, F., Annane, D., Arabi, Y., Beane, A., et al. (2020). Effect of Hydrocortisone on Mortality and Organ Support in Patients With Severe COVID-19: The REMAP-CAP COVID-19 Corticosteroid Domain Randomized Clinical Trial. JAMA. 324(13):1317-1329. doi: 10.1001/jama.2020.17022.

1. **Ren 2020**

- Ren, Z., Luo, H., Yu, Z., Song, J., Liang, L., Wang, L., et al. (2020). A Randomized, Open-label, Controlled Clinical Trial of Azvudine Tablets in the Treatment of Mild and Common COVID-19, A Pilot Study. Adv Sci (Weinh). 7(19):2001435. doi: 10.1002/advs.202001435. Epub ahead of print.

1. **Rocco 2020**

- Rocco, P.R.M., Silva, P.L., Cruz, F.F., Junior, M.A.C.M., Tierno, P.F.G.M.M., Moura, M.A., et al. (2020). Early use of nitazoxanide in mild Covid-19 disease: randomized, placebo-controlled trial. [Preprint]. Available at: https://www.medrxiv.org/content/10.1101/2020.10.21.20217208v1.article-info

1. **Rosas 2020**

- Rosas, I., Bräu, N., Waters, M., Go, R.C., Hunter, B.D., Bhagani, S., et al. (2020). Tocilizumab in Hospitalized Patients With COVID-19 Pneumonia. [Preprint].

Available at: https://www.medrxiv.org/content/10.1101/2020.08.27.20183442v2

1. **Ruzhentsova 2020**

- Ruzhentsova, T., Chukhliaev, P., Khavkina, D., Garbuzov, A., Oseshnyuk, R., Soluyanova, T., et al. (2020). Phase 3 Trial of Coronavir (Favipiravir) in Patients with Mild to Moderate COVID-19. Available at SSRN: https://ssrn.com/abstract=3696907 or <http://dx.doi.org/10.2139/ssrn.3696907>

1. **Sadeghi** **2020**

- Sadeghi, A., Ali Asgari, A., Norouzi, A., Kheiri, Z., Anushirvani, A., Montazeri, M., et al. (2020). Sofosbuvir and daclatasvir compared with standard of care in the treatment of patients admitted to hospital with moderate or severe coronavirus infection (COVID-19): a randomized controlled trial. J Antimicrob Chemother. 75(11):3379-3385. doi: 10.1093/jac/dkaa334.

1. **Sakoulas 2020**

- *Sakoulas, G., Geriak, M., Kullar, R., Greenwood, K.L., Habib, M., Vyas, A., et al. (2020). Intravenous Immunoglobulin Plus Methylprednisolone Mitigate Respiratory Morbidity in Coronavirus Disease 2019. Crit Care Explor. 2(11):e0280. doi: 10.1097/CCE.0000000000000280.
- Sakoulas, G., Geriak, M., Kullar, R., Greenwood, K.L., Habib, M., Vyas, A., et al. (2020). Intravenous Immunoglobulin (IVIG) Significantly Reduces Respiratory Morbidity in COVID-19 Pneumonia: A Prospective Randomized Trial. [Preprint].

Available at: https://www.medrxiv.org/content/10.1101/2020.07.20.20157891v1

1. **Salama 2020**

- Salama, C., Han, J., Yau, L., Reiss, W.G., Kramer, B., Neidhart, J.D., et al. (2020). Tocilizumab in nonventilated patients hospitalized with Covid-19 pneumonia. [Preprint].

Available at: https://www.medrxiv.org/content/10.1101/2020.10.21.20210203v1.

1. **Salvarani 2020**

- *Salvarani, C., Dolci, G., Massari, M., Merlo, D.F., Cavuto, S, Savoldi, L., et al; RCT-TCZ-COVID-19 Study Group. (2020). Effect of Tocilizumab vs Standard Care on Clinical Worsening in Patients Hospitalized With COVID-19 Pneumonia: A Randomized Clinical Trial. JAMA Intern Med. e206615. doi: 10.1001/jamainternmed.2020.6615. Epub ahead of print.
- Agenzia Italiana del Farmaco (AIFA). (2020). RCT-TCZ-COVID-19 Study Group. Efficacy of Early Administration of Tocilizumab in COVID-19 Patients. Available at: <https://www.aifa.gov.it/web/guest/-/covid-19-studio-randomizzato-italiano-nessun-beneficio-dal-tocilizumab>. [Accessed July 02, 2020].

1. **Sekhavati 2020**

- Sekhavati, E., Jafari, F., SeyedAlinaghi, S., Jamalimoghadamsiahkali, S., Sadr, S., Tabarestani, M., et al. (2020). Safety and effectiveness of azithromycin in patients with COVID-19: An open-label randomised trial. Int J Antimicrob Agents. 56(4):106143. doi: 10.1016/j.ijantimicag.2020.106143. Epub 2020 Aug 25.

1. **Self 2020**

- Self, W.H., Semler, M.W., Leither, L.M., Casey, J.D., Angus, D.C., Brower, R.G., et al, and Blood Institute PETAL Clinical Trials Network. (2020). Effect of Hydroxychloroquine on Clinical Status at 14 Days in Hospitalized Patients With COVID-19: A Randomized Clinical Trial. JAMA. 324(21):2165-2176. doi: 10.1001/jama.2020.22240.

1. **Shi 2020**

- Shi, L., Huang, H., Lu, X., Yan, X., Jiang, X., Xu, R., et al. (2020). Treatment with human umbilical cord-derived mesenchymal stem cells for COVID-19 patients with lung damage: a randomised, double-blind, placebo-controlled phase 2 trial. [Preprint].

Available at: https://www.medrxiv.org/content/10.1101/2020.10.15.20213553v2

1. **Shu 2020**

- Shu, L., Niu, C., Li, R., Huang, T., Wang, Y., Huang, M., et al. (2020). Treatment of severe COVID-19 with human umbilical cord mesenchymal stem cells. Stem Cell Res Ther. 11(1):361. doi: 10.1186/s13287-020-01875-5.

1. **Simonovic 2020**

- Simonovich, V.A., Burgos Pratx, L.D., Scibona, P., Beruto, M.V., Vallone, M.G., Vázquez, C., et al. and PlasmAr Study Group. (2020). A Randomized Trial of Convalescent Plasma in Covid-19 Severe Pneumonia. N Engl J Med. doi: 10.1056/NEJMoa2031304. Epub ahead of print.

1. **Spinner 2020**

- *Spinner, C.D., Gottlieb, R.L., Criner, G.J., Arribas López J.R., Cattelan, A.M., Soriano Viladomiu, A., et al and GS-US-540-5774 Investigators. (2020). Effect of Remdesivir vs Standard Care on Clinical Status at 11 Days in Patients With Moderate COVID-19: A Randomized Clinical Trial. JAMA. 2020 Sep 15;324(11):1048-1057. doi: 10.1001/jama.2020.16349.
- Gilead Announces Results From Phase 3 Trial of Remdesivir in Patients With Moderate COVID-19 available from https://www.gilead.com/news-and-press/press-room/press-releases/2020/6/gilead-announces-results-from-phase-3-trial-of-remdesivir-in-patients-with-moderate-covid-19 [Accessed on June 1, 2020.

1. **Stone 2020**

- Stone, J.H., Frigault, M.J., Serling-Boyd, N.J., Fernandes, A.D., Harvey, L., Foulkes, A.S., et al and BACC Bay Tocilizumab Trial Investigators. Efficacy of Tocilizumab in Patients Hospitalized with Covid-19. N Engl J Med. doi: 10.1056/NEJMoa2028836. Epub ahead of print.

1. **Tabarsi 2020**

- Tabarsi, P., Barati, S., Jamaati, H., Haseli, S., Marjani, M., Moniri, A., et al. (2020). Evaluating the effects of Intravenous Immunoglobulin (IVIg) on the management of severe COVID-19 cases: A randomized controlled trial. Int Immunopharmacol. doi: 10.1016/j.intimp.2020.107205. Epub ahead of print.

1. **Tang 2020**

- *Tang, W., Cao, Z., Han, M., Wang, Z., Chen, J., Sun, W., et al. (2020). Hydroxychloroquine in patients with COVID-19: an open-label, randomized, controlled trial. BMJ 369:m1849. doi: https://doi.org/10.1136/bmj.m1849.
- Tang, W., Cao, Z., Han, M., Wang, Z., Chen, J., Sun, W., et al. (2020). Hydroxychloroquine in patients with COVID-19: an open-label, randomized, controlled trial. medRxiv doi: https://doi.org/10.1101/2020.04.10.20060558.

1. **Tomazini 2020**

- Tomazini, B.M., Maia, I.S., Cavalcanti, A.B., Berwanger, O., Rosa, R.G., Veiga, V.C., et al and COALITION COVID-19 Brazil III Investigators. Effect of Dexamethasone on Days Alive and Ventilator-Free in Patients With Moderate or Severe Acute Respiratory Distress Syndrome and COVID-19: The CoDEX Randomized Clinical Trial. JAMA. 324(13):1307-1316. doi: 10.1001/jama.2020.17021.

1. **Udwadia 2020**

- Udwadia, Z.F., Singh, P., Barkate, H., Patil, S., Rangwala, S., Pendse, A., et al. (2020). Efficacy and Safety of Favipiravir, an Oral RNA-Dependent RNA Polymerase Inhibitor, in Mild-to-Moderate COVID-19: A Randomized, Comparative, Open-Label, Multicenter, Phase 3 Clinical Trial. Int J Infect Dis. S1201-9712(20)32453-X. doi: 10.1016/j.ijid.2020.11.142. Epub ahead of print.

1. **Ulrich 2020**

- Ulrich, R.J., Troxel, A.B., Carmody, E., Eapen, J., Bäcker, M., DeHovitz, J.A., et al. (2020). Treating Covid-19 With Hydroxychloroquine (TEACH): A Multicenter, Double-Blind, Randomized Controlled Trial in Hospitalized Patients. Open Forum Infect Dis. <https://doi.org/10.1093/ofid/ofaa446>

1. **Vlaar 2020**

- Vlaar, A.P.J., de Bruin, S., Busch, M., Timmermans, S., van Zeggeren, I.E., Koning, R., et al. (2020). Anti-C5a Antibody (IFX-1) Treatment of Severe COVID-19: An Exploratory Phase 2 Randomized Controlled Trial. TLRHEU-D-20-00358, Available at SSRN: https://ssrn.com/abstract=3658226 or <http://dx.doi.org/10.2139/ssrn.3658226>

1. **Wang D 2020**

- Wang, D., Fu, B., Peng, Z., Yang, D., Han, M., Li, M., et al. (2020). Tocilizumab Ameliorates the Hypoxia in COVID-19 Moderate Patients with Bilateral Pulmonary Lesions: A Randomized, Controlled, Open-Label, Multicenter Trial. Available at SSRN: https://ssrn.com/abstract=3667681; 2020.

1. **Wang M 2020**

- Wang, M., Zhao, Y., Hu, W., Zhao, D., Zhang, Y., Wang, T., et al. (2020). Treatment of COVID-19 Patients with Prolonged Post-Symptomatic Viral Shedding with Leflunomide -- a Single-Center, Randomized, Controlled Clinical Trial. Clin Infect Dis. doi: 10.1093/cid/ciaa1417. Epub ahead of print.

1. **Wang Y 2020**

- *Wang, Y., Zhang, D., Du, G., Du, R., Zhao, J., Jin, Y., et al. Remdesivir in adults with severe COVID-19: a randomised, double-blind, placebo-controlled, multicentre trial. Lancet. 395(10236):1569-1578. doi: 10.1016/S0140-6736(20)31022-9. Epub 2020 Apr 29.
- Erratum in: Lancet. 2020 May 30;395(10238):1694.Wang Y, Zhou F, Zhang D, et al. Evaluation of the efficacy and safety of intravenous remdesivir in adult patients with severe COVID-19: study protocol for a phase 3 randomized, double-blind, placebo-controlled, multicentre trial. Trials. 2020;21(1):422.

1. **Wu 2020**

- Wu, X., Yu, K., Wang, Y., Xu, W., Ma, H., Hou, Y., et al. Efficacy and safety of triazavirin therapy for coronavirus disease 2019: A pilot randomized controlled trial. Engineering (Beijing). 6(10):1185-1191. doi: 10.1016/j.eng.2020.08.011. Epub 2020 Sep 8.

1. **Yakoot 2020**

- Yakoot, M., Eysa, B., Gouda, E., Hill, A., Helmy, S.A., Elsayed, M.R., et al. (2020). Efficacy and Safety of Sofosbuvir/Daclatasvir in the Treatment of COVID-19: A Randomized, Controlled Study. SSRN Electronic Journal. Available at SSRN: https://ssrn.com/abstract=3705289 or <http://dx.doi.org/10.2139/ssrn.3705289>

1. **Zhao 2020**

- Zhao, H., Zhu, Q., Zhang, C., Li, J., Wei, M., Qin, Y., et al. (2020). Tocilizumab combined with favipiravir in the treatment of COVID-19: A multicenter trial in a small sample size. Biomed Pharmacother. 2021;133:110825. doi.org/10.1016/j.biopha.2020.110825.

1. **Zheng 2020**

- *Zheng, F., Zhou, Y., Zhou, Z., Ye, F., Huang, B., Huang, Y., et al. (2020). SARS-CoV-2 clearance in COVID-19 patients with Novaferon treatment: A randomized, open-label, parallel-group trial [published online ahead of print, 2020 Aug 3]. Int J Infect Dis. 2020;99:84-91. doi:10.1016/j.ijid.2020.07.053
- Zheng, F., Zhou, Y., Zhou, Z., Ye, F., Huang, B., Huang, Y., et al. (2020). A Novel Protein Drug, Novaferon, as the Potential Antiviral Drug for COVID-19. medRxiv 2020.04.24.20077735 [Preprint]

Available at: <https://www.medrxiv.org/content/10.1101/2020.04.24.20077735v1>

### **Appendix 4. Additional characteristics of studies included.**

**Table S1.** Additional characteristics of studies included.

| Study | Sponsorship | Diagnostic criteria | Drug/Intervention | Dose/Definition | Definition Standard care and other treatments |
| --- | --- | --- | --- | --- | --- |
| Abbaspour Kasgari 2020 | Mazandaran University of Medical Sciences. | Positive qualitative RT–PCR for SARS-CoV-2 and/or features consistent with COVID-19 on a chest CT scan | Sofosbuvir plus Daclastavir plus Ribivarin | sofosbuvir/daclatasvir dose of 400/60mg and ribavirin 600mg twice daily | Standard care: hydroxychloroquine  (400mg single dose) and lopinavir/ritonavir (400/100mg twice daily), with  or without ribavirin (600mg twice daily) |
|  |  |  | Standard care |  |  |
| Abd-Elsalam 2020a | NR | Mild: uncomplicated viral upper respiratory tract infection. Moderate: pneumonia but without the need for supplemental oxygen. Severe: fever or suspected respiratory infection, plus one of the following symptoms: respiratory rate> 30 breaths / min, severe respiratory distress, or 93% SpO2 on ambient air | Hydroxychloroquine | 400 mg twice daily (in day 1) followed by 200 mg tablets twice daily for 15 days. | Paracetamol, oxygen, fluids (according to assessment), empiric antibiotic (cephalosporins), oseltamivir if needed (75 mg/12 hours for 5 days), and invasive mechanical ventilation with hydrocortisone for severe cases if PaO2 < 60 mmHg, O2 saturation < 90% despite oxygen or non-invasive ventilation, progressive hypercapnia, respiratory acidosis (pH < 7.3), and progressive or refractory septic shock. |
|  |  |  | Standard care |  |  |
| Abd-Elsalam 2020b | Tanta University | Positive RT-PCR result for SARSCoV-2. Mild: symptoms of COVID-19 infection but not complicated with pneumonia or hypoxia. Moderate: mild viral pneumonia and SpO2 > 90% on room air. Severe: signs of severe pneumonia such as respiratory rate > 30 breaths/min, severe respiratory distress, or SpO2 < 90% on room air. Critical: acute respiratory distress syndrome, sepsis, and septic shock | Hydroxychloroquine plus Zinc | Zinc: zinc sulfate 220 mg (50 mg of elemental zinc) twice daily Hydroxychloroquine: 400 mg twice daily on the first day, then 200 mg twice daily for 5 days | Both groups received the Standard Care treatment for COVID-19 infection, according to the Egyptian Ministry of Health guidelines for 15 days, but details are not reported. |
|  |  |  | Hydroxychloroquine |  |  |
| Agarwal 2020 (PLACID trial) | Indian Council of Medical Research (ICMR), an autonomous government funded medical research council. | Positive RT-PCR result for SARSCoV-2.  moderate illness with either a PaO2/FiO2 ratio between 200 mm Hg and 300 mm Hg or a respiratory rate of more than 24/min with oxygen saturation (SpO2) 93% or less on room air | c-Plasma* | two doses of 200 mL transfused 24 hours apart | Antivirals (hydroxychloroquine, remdesivir, lopinavir/ritonavir, oseltamivir), broad spectrum antibiotics, immunomodulators (steroids, tocilizumab), and supportive management (oxygen through a nasal cannula, face mask, non-rebreathing face mask; non invasive or invasive mechanical ventilation; awake proning). |
|  |  |  | Standard care |  |  |
| AlQahtani 2020 | Ministry of Health Bahrain and the College of Surgeons in Ireland-Bahrain | COVID-19 confirmed by PCR, hypoxia (Oxygen saturation of 88 less than or equal 92% on air, or PO2 < 60mmHg in arterial blood gas, or PaO2/FiO2 of 300 or less) and patient requiring oxygen 90 therapy, pneumonia confirmed by chest imaging. | c-Plasma | 400ml: 200ml in 2 hours for 2 days | Paracetamol, antivirals, tocilizumab, antibacterials |
|  |  |  | Standard care |  |  |
| Ansarin 2020 | Tabriz University of Medical Sciences. Iran | Pneumonia made by a board-certified pulmonologist based on clinical symptoms and signs, as well as chest CT findings compatible with the COVID-19 pneumonia pattern | Bromhexine hydrochloride | 8 mg three times a day, oral | All patients received national COVID-19 treatment protocol and best practice guidelines and also the “Hydroxychloroquine 200 mg/d for two weeks” in addition to supportive and symptomatic therapy. |
|  |  |  | Standard care |  |  |
| Avendaño-Solà 2020 | Government of Spain, Spanish Instituto de Salud Carlos III | Confirmed SARS-CoV-2 infection (RT-PCR) with either radiographic evidence of pulmonary infiltrates or clinical evidence plus SpO2 ≤94% on room air, and within 12 days from the onset of symptoms (fever or cough) | c-Plasma | 250-300 mL, single unit | All supportive and specific treatments with off-label marketed medicines used according to local or  national recommendations |
|  |  |  | Standard care |  |  |
| Bajpal 2020 | NR | SARS-CoV-2 infection confirmed byRT-PCR, severe COVID-19: respiratory rate (RR) ≥30/min, oxygen saturation level less than 93% in resting state, the partial pressure of oxygen (PaO2)/oxygen concentration (FiO2) ≤300 mmHg, lung infiltrates >50% within 24 to 48 hours | c-Plasma | 500ml in two doses | All the patients in the study were initiated on supplemental oxygen at five litter/min with target SpO2 being ≥94%. If saturation remained below 94%, either of  high flow Oxygen or NIV (via BiPAP) was given. Medically, all patients received a course of  Hydroxychloroquine 400 mg BD on Day1, followed by 200 mg BD for five days along with  Oral Azithromycin 500 mg OD for five days. |
|  |  |  | fresh frozen Plasma | 500ml in two doses |  |
| Beigel 2020 | Public: National Institute of Allergy and Infectious Diseases (NIAID), National Institutes of Health (NIH) | Confirmed SARS-CoV-2 infection by a positive RT-PCR assay result from any respiratory specimen collected <72 hours prior to randomization. and ≥72 hours prior to randomization   Severe: Invasive or non-invasive mechanical ventilation required, requiring supplemental oxygen, SpO2 ≤ 94% on ambient air, or tachypnea (respiratory rate ≥ 24 breaths per minute)  Mild/moderate: SpO2 > 94% and respiratory rate < 24 breaths per minute without additional oxygen. | Remdesivir | 200 mg loading dose on day 1, followed by 100 mg daily for up to 9 additional days | Standard Care: All patients received supportive care according to the standard care for the trial site hospital other experimental treatment or off-label use of marketed medications intended as specific treatment for Covid-19 were prohibited from day 1 through day 29 |
|  |  |  | Placebo | Same volume of Remdesivir |  |
| Brown 2020 | Heart and Lung Research Foundation, Intermountain Research and Medical Foundation, Office of the Associate Vice President for Research, University of Utah Health Sciences | symptomatic laboratory-confirmed COVID-19, within 10 days of a positive test for COVID-19 | Hydroxychloroquine | Administered orally as a loading dose of 400mg twice on the first day followed by 200mg twice daily for the following 4 days (total dose 2.4gm) or until discharge or death | NR |
|  |  |  | Azithromycin | Administered orally as a loading dose of 500mg on the first day, followed by 250mg daily for the next 4 days (total dose 1.5gm) or until discharge or death. |  |
| Cao B 2020 | Major Projects of National Science and Technology on New Drug Creation and Development; the Chinese Academy of Medical Sciences Emergency Project of Covid-19; National Science Grant for Distinguished Young Scholars | Positive RT-PCR assay for SARS-CoV-2 in a respiratory tract sample, pneumonia confirmed by chest imaging, oxygen saturation (SaO2) of 94% or less while they were breathing ambient air or a ratio of the partial pressure of oxygen (PaO2) to the fraction of inspired oxygen (FiO2) (PaO2:FiO2) at or below 300 mg Hg | Lopinavir–ritonavir | 400 mg and 100 mg, orally | Supplemental oxygen, noninvasive and invasive ventilation, antibiotic agents, vasopressor support, renal-replacement therapy, and extracorporeal membrane oxygenation |
|  |  |  | Standard Care |  |  |
| Cao Y 2020 | NR | Severe COVID-19 according to the "Chinese management guideline for 150 COVID-19 (version 5.0)" | Ruxolitinib | 5mg twice a day | Antiviral therapy, supplemental oxygen, non-invasive and invasive ventilation, corticosteroids, antibiotic agents, vasopressor support, renal replacement therapy and extracorporeal membrane oxygenation (ECMO) |
|  |  |  | Standard Care | 100 mg vitamin C |  |
| Cavalcanti 2020 | Institutions participating in the Coalition Covid-19 Brazil and by EMS Pharma | Confirmed COVID-19 by RT-PCR.  Suspected cases based on the Brazilian Ministry of Health criteria: patients with fever and at least 1 respiratory sign or symptom (cough, shortness of breath, nasal congestion, sore throat, peripheral oxygen saturation <95%, cyanosis, dyspnea); those from an endemic region or traveling from an endemic region in the last 14 days | Hydroxychloroquine plus Azithromycin | hydroxychloroquine 400 mg twice daily + azithromycin 500 mg once daily | Respiratory therapy and motor physiotherapy, surveillance of vital parameters, addition of ventilatory support measures, such as increased oxygen flow, use of non-invasive positive pressure ventilation or oxygen supplementation via high-flow nasal cannula, prophylaxis of stress ulcers and venous thromboembolism according to the protocol of each institution and addition of other therapies such as antibiotics, corticosteroids, other immunomodulators (e.g., tocilizumab), other antivirals (e.g., oseltamivir for suspected influenza coinfection), as recommended by the attending physician. |
|  |  |  | Hydroxychloroquine | 400 mg twice daily |  |
|  |  |  | Standard Care |  |  |
| Chen C 2020 | National Key Research and Development Program of China | Confirmed COVID-19 to test positive by qRT-PCR | Favipiravir | 1600mg/time on the first day, twice a day, 600mg/time from day 2 to the end of the experiment, twice a day. | Routine therapy but the details are not reported. Other drugs were used for conventional therapy and symptomatic treatment to improve adverse reactions: Antibiotic, Antiviral drugs Glucocorticoid, Chinese herbal medicine, Psychotropic substances, Immunomodulator, Nutritional support |
|  |  |  | Umifenovir | 200 mg each time, 3 times a day, from the first day to the end of the trial |  |
| Chen CP 2020 | Taiwan Hospital and Social Welfare Organizations Administration Commission, Ministry of Health and Welfare; Taiwan Biotech  Co. Ltd. | Real-time reverse transcription polymerase chain reaction (rRT-PCR).  Mild: without evidence of infiltration according to chest roentgenography;  Moderate: evidence of infiltration according to chest roentgenography but neither respiratory distress nor supplemental oxygen requirement;  Severe: with respiratory distress, oxygen supplementation, and evidence of infiltration according to chest roentgenography. Participants presenting with severe illness were excluded from this study | Hydroxychloroquine | 400 mg b.i.d. on  day 1 and 200 mg b.i.d. for 6 days on days 2–7 | ceftriaxone 2 g daily for 7 days ± azithromycin 500 mg on day 1 and 250 mg on days 2–5;  or levofloxacin 750 mg daily for 5 d;  or levofloxacin 500 mg daily;  or moxifloxacin 400 mg daily for 7–14 days for  subjects allergic to ceftriaxone or azithromycin or according to physician discretion.  Oseltamivir 75 mg b.i.d. will be administered for 5 days to subjects presenting with concomitant influenza A or B infection. |
|  |  |  | Standard care |  |  |
| Chen J 2020a | NR | COVID-19 confirmed according to the "diagnosis and treatment plan". | Hydroxychloroquine | 400 mg per 5 days | Oxygen therapy, and symptomatic supportive treatment. Viral drugs such as alpha interferon nebulization, oral lopinavir / ritonavir (clepivir), etc., and antibacterial drugs are given if necessary. (66,7% umifenovir, 13,3% lopinavir/ritonavir).  In Hydroxychloquine arm 80% received umifenovir |
|  |  |  | Standard Care |  |  |
| Chen J 2020b | Ministry of Science and Technology of China; the Shanghai Science and Technology Committee; Shanghai Major Projects on Infectious Diseases; and the Shanghai “Rising Stars of Medical Talent” Youth Development Program, Specialist Program | SARS-CoV-2 infection confirmed in the laboratory | Darunavir/Cobicistat | A single tablet containing 800 mg of darunavir and 150 mg of cobicistat | Interferon alpha 2b and the standards of care according to the recommendations of the guidelines in China |
|  |  |  | Standard Care |  |  |
| Chen L, 2020 | Medical and Health Key project of Xiamen, which is a project of the Xiamen Science and Technology Bureau. | COVID-19 mild or moderate according to the Chinese diagnosis and treatment protocol for new Coronavirus pneumonia (5th -7th edition). RT-PCR positive patients for SARS-CoV-2 or COVID-19 characteristic lung changes on chest CT, SaO2 > 93%. | Chloroquine | 1000 mg QD per day 1, then 500 mg QD for 9 days | Chinese diagnosis and treatment protocol for new coronavirus pneumonia (5th edition) |
|  |  |  | Hydroxychloroquine | 200 mg BID for 10 days |  |
|  |  |  | Standard care |  |  |
| Chen P 2020 | Eli Lilly and Company | One or more mild or moderate COVID-19 symptoms (Fever, cough, sore throat, malaise, headache, muscle pain, gastrointestinal symptoms, or shortness of breath with exertion); sample taken for test confirming viral infection no more than 3 days prior to starting the drug infusion | Neutralized antibody LY-CoV555 | Single intravenous infusion monotherapy  over approximately 1 hour.700 mg, 2800 mg, and 7000 mg | Initiate/expand enrollment to an additional/existing LY3819253 treatment arm (or arms). |
|  |  |  | Placebo | 0.9% sodium chloride solution |  |
| Chen Z 2020 | NR | Positive for SARS-CoV-2 to RT-PCR test, pneumonia; SaO2/SPO2 ratio > 93% or PaO2/FIO2 ratio > 300 | Hydroxychloroquine | 400 mg/d (200 mg/2 times daily) | Oxygen therapy, antiviral agents, antibacterial agents, and immunoglobulin, with or without corticosteroids |
|  |  |  | Standard Care |  |  |
| Cheng 2020 | Grants related to the prevention and management of coronavirus disease 2019 and Guangzhou Institute for Respiratory Health. | Positive test results RT-PCR assay for severe acute respiratory syndromecoronavirus 2 (SARSCoV-2) in oropharyngeal samples; pneumonia as confirmed by chest imaging and aPBLcell count of 800 per μL(toconvert to×109/L, multiply by 0.001) or lower | Human-Granulocyte-Colony–Stimulating Factor(rhG-CSF) | 500 mg once daily (by oral, nasogastric, or intravenous route) | Without macrolides, at the discretion of treating physicians and according to local guidelines. Use of corticosteroids, other immunomodulators, antibiotics, and antivirals was allowed. |
|  |  |  | Standard care |  |  |
| Chowdhury 2020 | Authors state: Funding: Not applicable. | Positive for SARS-CoV-2 infection by RT PCR | Ivermectin plus Doxycycline | Ivermectin 200μgm/kg single dose, Doxycycline 100 mg BID for 10days | NR |
|  |  |  | Hydroxychloroquine plus Azithromycin | Hydroxychloroquine 400 mg 1st day then200mg BID for 9days + Azithromycin 500 mg for 5Days. |  |
| Corral-Gudino 2020 | No specific funding | Laboratory confirmed diagnosis of SARS-CoV2 infection.  1) Symptom duration of at least 7 days, 2) Radiological evidence of lung disease in chest X-ray or CT-scan, 3) Moderate-to-severe disease with abnormal gas exchange: PaFi (PaO2/FiO2) < 300, or SAFI (SAO2/FiO2) < 400, or at least 2 criteria of the BRESCIA-COVID Respiratory Severity Scale (BCRSS), 4) Laboratory parameters suggesting a hyper-inflammatory state: serum C-Reactive Protein (CRP) >15 mg/dl, D-dimer > 800 mg/dl, ferritin > 1000 mg/dl or IL-6 levels > 20 pg/ml. | Corticosteroid (Metylprednisolone) | 40 mg intravenously every 12 hours for 3 days and then 20 mg every 12 hours for 3 days. | Symptomatic treatment with acetaminophen, oxygen therapy, thrombosis prophylaxis with low molecular weight heparin, and antibiotics for co-infections. Azythromycin, hydroxychloroquine and lopinavir plus ritonavir were frequently prescribed. |
|  |  |  | Standard care |  |  |
| Dabbous 2020 | Rameda Pharmaceutical Company | Confirmed COVID-19 by a diagnostic laboratory test (e.g. nasopharyngeal swab) and having mild to moderate symptoms according to the national protocol classifcation | Favipiravir | 3200mg at day1 followed by 600mg twice (day2-day10). | Hydroxychloroquine 800mg at day1 followed  by 200mg twice (day2- 10) and oral oseltamivir 75mg/12hour/day for 10 days.  All patients received enoxaparin 40mg subcutaneous for 14 days or 1mg/kg every 12 hours in case Ddimers>1000ng/ml for one month. |
|  |  |  | Standard care |  |  |
| Davoudi-Monfared E, 2020 | No funding. ReciGen was a generous gift from CinnaGen Co. | COVID-19 confirmed with RT-PCR of respiratory tract or clinical signs/symptoms and highly suspect imaging results for COVID-19. | Interferon beta 1a | 44 micrograms/ml (12 million IU/ml) subcutaneous | Hospital protocol: hydroxychloroquine (400 mg BD on the first day and then 200 mg BD) plus lopinavir/ritonavir (400/100 mg BD) or atazanavir/ritonavir (300/100 mg per day) for 7-10 days. Primary care, respiratory support, fluids, electrolytes, analgesics, antipyretics, corticosteroids and antibiotics have also been recommended in the hospital protocol if indicated. |
|  |  |  | Standard care |  |  |
| Deftereos SG, 2020 | ELPEN Pharmaceuticals, Acarpia Pharmaceuticals, Karian  Pharmaceuticals | SARS-COV-2 confirmed by RT PCR, clinical symptoms (e.g. body temperature >37.5). At least two of the following criteria: persistent cough, persistent throat pain, anosmia, ageusia, asthenia; partial blood pressure of oxygen (PaO2)<95 mmHg | Colchicine | 1.5 mg loading dose followed by 0.5 mg after 60 minutes and maintenance doses of 0.5 mg twice daily | Medical treatment for COVID-19 per local protocols |
|  |  |  | Standard care |  |  |
| Dequin 2020 | French Ministry of Health, Programme Hospitalier de Recherche Clinique (PHRC) (2014 [CAPE COD parent trial], 2020 [CAPE COVID subtrial]). | Biologically confirmed COVID-19 (reverse transcriptase–polymerase chain reaction) or suspected (suggestive chest computed tomography scan result in the absence of any other cause of pneumonia). To receive hydrocortisone one of 4 severity criteria had to be present: need for mechanical ventilation with a positive end-expiratory pressure (PEEP) of 5 cmH20 or more; a ratio of PaO2/FIo2 less than 300 on high-flow oxygen therapy with an FIO2 value of at least 50%; for patients receiving oxygen through a reservoir mask, a PaO2:FIO2 ratio less than 300, estimated using prespecified charts; or a Pulmonary Severity Index18 greater than 130. | Corticosteroid (Hydrocortison) | Initial dose of 200mg/d. Treatment was continued at 200mg/d until day 7 and then decreased to 100 mg/d for 4 days and 50 mg/d for 3 days, for a total of 14 days. If the patient’s respiratory and general status had sufficiently improved by day 4, a short treatment regimen was used (200mg/d for 4 days, followed by 100mg/d for 2 days and then 50 mg/d for the next 2 days, for a total of 8 days). | NR |
|  |  |  | Placebo |  |  |
| Duarte 2020 | The trial was funded by the participating hospitals. Laboratorio Elea Phoenix S.A. | PCR-confirmed Covid-19 infection with 4 or fewer days elapsed since symptom onset | Telmisartan | 80 mg twice daily for 14 days | NR |
|  |  |  | Standard Care |  |  |
| Dubèe 2020 | French Ministry of Health, Pays de la Loire region, Angers Loire Metropole conurbation | Diagnosis of COVID-19 confirmed by positive RT-PCR SARS-CoV-2 or by thorax CT-scan showing typical features of COVID-19. At least one of the following risk factors for worsening: age ≥75 years old; Age between 60 and 74 years old and presence of at least one comorbidity among the following: obesity (body mass index ≥ 30 kg/m²), arterial hypertension requiring treatment, diabetes mellitus requiring treatment; Need for supplemental oxygen to reach a peripheral capillary oxygen saturation of more than 94% (SpO2>94%), or PaO2/FiO2 ≤ 300 mmHg. | Hydroxychloroquine | 800mg on Day 0 followed by 400mg per day for 8 days (200mg tablets, orally) | Concomitant treatment with azithromycin, lopinavir/ritonavir, treatment with corticosteroids, and centre. |
|  |  |  | Standard care |  |  |
| Edalatifard 2020 | Deputy of Research, Tehran University of Medical Sciences | Identification of SARS-CoV-2 via RT-PCR in nasopharyngeal swab or sputum samples and abnormal CT scan finding (bilateral, subpleural, peripheral ground-glass opacities) with oxygen saturation <90% at rest. | Coricosteroids (methylprednisolone) | intravenous injection, 250mg/day | Hydroxychloroquine sulfate, Lopinavir, and Naproxen |
|  |  |  | Standard care |  |  |
| Entrenas Castillo 2020 | NR | Acute respiratory infection, confirmed by a radiographic pattern of viral pneumonia and by a positive SARS-CoV-2 PCR with CURB65 severity scale (recommending hospital admission in case of total score > 1) | Calcifediol plus standard care | soft capsules (0.532 mg). Patients continued with oral calcifediol (0.266 mg) on day 3 and 7, and then weekly until discharge or ICU admission | Hydroxychloroquine (400 mg every 12 hours on the first day, and 200 mg every 12 hours for the following 5 days), azithromycin (500 mg orally for 5 days) and for patients with pneumonia and NEWS score≥5, a broad spectrum antibiotic (ceftriaxone2 g intravenously every 24 hours for 5 days) |
|  |  |  | Standard care |  |  |
| Esquivel-Moynelo, 2020 | Center for Genetic Engineering and Biotechnology and Ministry of Health of Cuba.) | COVID-19 confirmed by RT-PCR on the E gene and RdRP gene with throat swabs. | Interferon α 2b plus Interferon γ | 3 milion IUs interferon alpha 2b, 0.5 milion IUs interferon gamma twice a week for two weeks; | All patients received 200/500mg lopinavir/ritonavir 12 hrs daily; 250 mg chloroquine 12 hrs daily. |
|  |  |  | Interferon α 2b | 3 milion IUs interferon alpha 2b tree times to week; |  |
| Furtado 2020 | COALITION COVID-19 Brazil. EMS | Suspected or confirmed COVID-19 with less than 14 days from the onset of the symptom. At least one of the following severity criteria: use of oxygen supplementation greater than 4 L / min of flow; use of high-flow nasal cannula; use of non-invasive positive pressure ventilation; or use of mechanical ventilation. | Hydroxychloroquine plus Azithromycin | Azithromycin: 500 mg via oral, nasogastric, or intravenous administration once daily for 10 days  Hydroxychloroquine: 400 mg twice daily for 10 days | Without macrolides, at the discretion of treating physicians and according to local guidelines. Use of corticosteroids, other immunomodulators, antibiotics, and antivirals was allowed. |
|  |  |  | Hydroxychloroquine |  |  |
| Gharbham 2020 | NR | Positive for COVID-19 by RT-PCR test in the previous 96 hours | c-Plasma | 300 ml intravenously | Depending on the hospital, off-label use of EMA-approved drugs (e.g. chloroquine, azithromycin, lopinavir/ritonavir, tocilizumab, anakinra) as treatment for COVID-19 was allowed in hospitals where this was part of the SC |
|  |  |  | Standard care |  |  |
| Gharebaghi 2020 | Urmia University of Medical Sciences. Iran | Positive for COVID-19 by RT-PCR and chest CT scan findings, involvement of > than 30% of both lungs (ground-glass opacity) in high-resolution computed tomography (HRCT) (confirmed by two radiologists), O2 saturation of < 90%, and a lack of adequate response to initial treatment. | Immunoglobulin | 5 flebogamma 5% four vials of 5 gm5 daily for three consecutive days | All patients continued to receive initial treatmens including methods including at least both one antiviral and one chloroquine-class drug |
|  |  |  | Placebo |  |  |
| Guvenmez 2020 | No financial support | Positive for SARS-CoV-2 by real-time RT-PCR assay; evidence of pneumonia in chest computerized tomography compatible with COVID-19 | Lincomycin | 600 mg twice a day for 5 days, intravenously | NR |
|  |  |  | Azithromycin | 500 mg for the first day and 250 mg  for days 2–5, oral |  |
| Hashim 2020 | Baghdad-Alkarkh General Directorate of Health in Bghadad, Iraq | Mild-moderate patients were outpatients while severe and critical patients were all inpatients. All of the recruited COVID-19 patients were diagnosed by clinical, radiological and laboratory PCR testing. | Ivermectin plus Doxycycline | Ivermectin 200ug/kg PO per day for two days, and in some patients who needed more time to recover, a third dose 200ug/kg PO per day was given 7 days after the first dose.  Doxycycline 100mg capsule PO every 12h per day was given for 5-10days, based on the clinical improvement of patients | All or some of the following, according to the clinical condition of each patient: Acetaminophen 500mg on need; Vitamin C 1000mg twice/ day; Zinc 75-125 mg/day; Vitamin D3 5000IU/day; Azithromycin 250mg/day for 5 days; Oxygen therapy/ C-Pap if needed; Dexamethazone 6 mg/day or methylprednisolone 40mg twice per day, if needed; Mechanical ventilation, if needed |
|  |  |  | Standard care |  |  |
| Hermine 2020 | Ministry of Health, Programme Hospitalier de  Recherche Clinique, France | Moderate or severe: confirmed SARS-CoV-2 infection (positive on rRT-PCR and/or typical chest computed tomographic [CT] scan) with moderate, severe, or critical pneumonia (O2 >3 L/min,WHO Clinical Progression Scale [WHO-CPS] score ≥5 | Tocilizumab | 8 mg/kg on day 1, intravenously. Administration of an additional fixed dose of Tocilizumab, 400 mg IV, on day 3 was recommended if oxygen requirement was not decreased by more than 50%, but decision was left to the treating physician. | All patients received antibiotic agents, antiviral agents, corticosteroids, vasopressor support, anticoagulants |
|  |  |  | Standard care |  |  |
| Huang 2020 | NR | Positive for SARS-CoV-2 by RT-PCR assay | Chloroquine | 500mg orally twice-daily | NR |
|  |  |  | Lopinavir/Ritonavir | 400 mg and 100 mg, orally twice-daily |  |
| Huang Y-Q 2020 | National Science and Technology Major Project of China During the 13th Five-year Plan Period; Chongqing Special Research Project for Prevention and Control of Novel Coronavirus Pneumonia | Moderate: nasopharyngeal or oropharyngeal swab or in the lower respiratory tract positive for SARS-CoV-2; fever, cough, or dyspnea; radiography or computed tomography evidence of interstitial pneumonia; respiratory requency (RR)<30 times/min; oxygen saturation (resting state) >93%; PaO2/FiO2>39.9 kPa.  Severe: SARS-CoV-2 by RTPCR in nasopharyngeal swab or blood samples; at least one of the following clinical conditions: respiratory distress (≥30 times per minute); oxygen saturation ≤93% at rest; PaO2)/ FiO2 ≤39.9 kPa; respiratory failure requiring mechanical ventilation; septic shock; and critical organ failure requiring intensive care | Ribavirin plus Interferon α | Intravenous injection of ribavirin at a loading dose of 2 g, followed by oral doses of 400-600 mg every 8 hours depending on the patient's body weight.  5 million Units or 50 mg per dose of interferon α  400 mg/100 mg lopinavir/ritonavir per dose twice | NR |
|  |  |  | Ribavirin plus Interferon α plus lopinavir/ritonavir |  |  |
|  |  |  | Lopinavir/Ritonavir plus interferon α |  |  |
| Hung 2020 | Department of Health of Hong Kong | Positive for COVID-19, a national early warning score 2 (NEWS2) of at least 1, and symptom duration of 14 days or less upon recruitment | Lopinavir+ritonavir+ribavirina+interferone beta-1b | lopinavir 400 mg and ritonavir 100 mg every 12 h, ribavirin 400 mg every 12 h, and three doses of 8 million international units of interferon beta-1b on alternate days | Concomitant treatment: oxygen therapy n=12; non-invasive ventilation n=3; supportive ventilation n=0; antibiotics n=44; corticosteroids n=6. |
|  |  |  | Lopinavir/ritonavir | lopinavir 400 mg/ritonavir 100 mg every 12 hours | Concomitant treatment: oxygen therapy n=5; non-invasive ventilation n=2; supportive ventilation n=1; antibiotics n=25; corticosteroids n=2. |
| Ivashchenko 2020 | Russian Direct Investment Fund, the Ministry of Industry and Trade of the Russian Federation, The Skolkovo Innovation Center | Moderate COVID-19 confirmed by PCR | Favipiravir | 1600mg BID day 1 following 600 mg days 2-14 | According to Russian guidelines for the treatment of COVID-19 |
|  |  |  | Favipiravir | 1800mg BID day 1 following 800 mg days 2-14 |  |
|  |  |  | Standard care |  |  |
| Jeronimo 2020 | Superintendência da Zona Franca de Manaus (SUFRAMA), Coordenação de Aperfeiçoamento de Pessoal de Nível Superior (CAPES), Departamento de Ciência e Tecnologia/Ministério da Saúde (DECIT), Ministério da Ciência, Tecnologia e Inovações (MCTI), Conselho Nacional de Desenvolvimento Científico e Tecnológico, Fundação de Amparo à Pesquisa do Estado do Amazonas (FAPEAM) | Radiological suspicion of COVID-19 (history of fever AND any respiratory symptom, e.g., cough or dyspnea AND/OR ground glass opacity OR pulmonary consolidation on CT scan), SpO2 < 94% at room air OR in use of supplementary oxygen OR under Invasive Mechanical Ventilation. | Corticosteroid (methylprednisolone) | 0.5 mg/kg twice in a day | All patients received a combination of ceftriaxone plus a macrolide.  All patients meeting ARDS criteria used pre-emptively intravenous ceftriaxone (1g 2x for 7 days) plus azithromycin (500 mg 1x for 5 days) or clarithromycin (500 mg 2x for 7 days), starting on day 1. |
|  |  |  | Placebo | saline solution |  |
| Kamran 2020 | NR | Mild: Confirmed SARS-CoV-2 infection by  positivity of RT-PCR of combined oropharyngeal and nasopharyngeal swabs | Hydroxychloroquine | 400 mg, oral, twice a day for day one followed by 200 mg 12 hourly for next 4 days | Vitamin C, vitamin D,zinc and tablet Paracetamol |
|  |  |  | Standard care |  |  |
| Khamis 2020 | No funding. | Confirmed SARS-CoV-2 infection by RT-PCR test on respiratory tract specimens, moderate to severe COVID-19 pneumonia according to the WHO interim guidelines case definitions (WHO/2019 nCoV/ Surveillance Case Definition /2020.1), the interval between symptoms onset and randomization is no >10 days | Favipiravir plus Interferon β 1b | Favipiravir: 1600mg giorno 1 poi 600mg 2 volte/giorno per massimo 10giorni,  Interferon β 1b: 8 milion IU (0.25 g) | Standard care based on the national guidelines that had hydroxychloroquine |
|  |  |  | Hydroxychloroquine | 400mg 2 times/day day 1 and then 200mg 2 times/day for 7 days |  |
| Krolewiecki 2020 | Agencia Nacional de Promoción de la Investigación, el Desarrollo Tecnológico y la Innovación, Argentina and Laboratorio ELEA/Phoenix, Argentina (grant IP-COVID-19-625) | 18 to 69 years-old with RT-PCR confirmed  infection, hospitalized with disease stages 3 to 5 from the WHO 8-Category ordinal scale of  clinical status and no requiring intensive care unit admission; symptoms onset ≤ 5 days at recruitment | Ivermectin | Ivermectine 6 mg ranurated tablets, at a  dose of 0.6mg/kg/day based on baseline weight rounding to the lower full (6mg) and half (3mg) dose. | NR |
|  |  |  | Standard Care |  |  |
| Kumar 2020 | Biocon Biologics India Limited | Moderate to severe confirmed SARS-CoV-2 infection by RT-PCR test, >18 years patients, hospitalized due to clinical worsening with oxygen saturation of ≤94% at rest in ambient air. PaO2/Fio2 ratio of < 200 or more than 25% deterioration from the immediate previous value. Alternatively, the proinflammatory markers included were baseline serum ferritin level ≥ 400 ng/mL or IL-6 levels greater than 4 times of upper limits of normal value. | Itolizumab | Patients were initiated on 1.6 mg/kg dose IV infusion of Itolizumab and continued with 0.8 mg/kg dose weekly regimen | Most commonly used therapies as part of best supportive care in both treatment arms included oxygen, antibiotics, hydroxychloroquine, antivirals, steroids, low-molecular-weight heparin, and vitamin supplements |
|  |  |  | Standard Care |  |  |
| Lenze 2020 | Taylor Family Institute for Innovative Psychiatric Treatment at Washington University; COVID-19 Early Treat Fund; Center for Brain Research in Mood Disorders at Washington University; Bantly Foundation; National Inst | Adults living in the community with SARS-CoV-2 infection confirmed by polymerase chain reaction assay and who were symptomatic within 7 days of the first dose of study medication (fever, cough, myalgia, mild dyspnea, diarrhea, vomiting, anosmia, ageusia, sore throat) | Fluvoxamine | 50mg of fluvoxamine(ormatching placebo) in the evening immediately after the baseline assessment and confirmation of eligibility, then for 2 days at a dose of 100mg twice daily as tolerated, and then increasing to a dose of 100 mg 3 times daily as tolerated through day 15  then stopped | NR |
|  |  |  | Placebo |  |  |
| Li L 2020 | Chinese Academy of Medical Sciences Innovation  Fund for Medical Sciences (CIFMS) grants  and the Nonprofit  Central Research Institute Fund of Chinese  Academy of Medical Sciences grant | Positive for COVID-19 by RT-PCR test  result within 72 hours prior to randomization; pneumonia confirmed by chest imaging; clinical symptoms meeting the definitions of severe or life-threatening COVID-19.  Severe COVID-19: respiratory distress (≥30 breaths/min; in resting state, oxygen saturation of 93% or less on room air; or PaO2/FIO2 of 300 or less. LifethreateningCOVID-19: respiratory failure requiring mechanical ventilation; shock; or other organ failure (apart from lung) requiring intensive care unit (ICU) monitoring | c-Plasma | 4-13 ml/kg body weight | Support assistance for COVID-19, based primarily on China's evolving national COVID-19 treatment guidelines and hospital practice. Possible treatments include antiviral drugs, antibacterial drugs, steroids, human immunoglobulins, Chinese herbal medicines and other drugs. |
|  |  |  | Standard care |  |  |
| Li T 2020 | Special Project for Significant New Drug Research and  Development in the Major National Science and Technology Projects of China | According to National Health Commission of People’s Republic of China the diagnosis and treatment plan (5th Edition) for the novel coronavirus disease.  Mild: clinical symptoms were mild and no pneumonia manifestations on chest radiograph. Moderate: respiratory and other symptoms onset, and with pneumonia manifestations on chest radiograph.  Severe (or critical): any of the following is fulfilled: 1) respiratory distress ≥ 30 times / min; 2) SpO2 ≤ 93% in 52 resting state; 3) oxygenation index ≤ 300mmHg (1 mmHg=0.133kPa); 4) respiratory failure occurs and requires mechanical ventilation; 5) shock appears; 6) combined with other organ failure, ICU monitoring and treatment required | Bromhexine Hydrochloride | Tablets (32 mg tid) after meals for 14 consecutive 76 days. Treatment was discontinued once the patient met the discharge criteria | Antiviral drugs, including arbidol hydrochloride granules (0.1g-73 0.2g tid) and recombinant human interferon α2b spray (0.083ml tid), |
|  |  |  | Standard care |  |  |
| Lopes 2020 | FAPESP, CNPq  and CAPES grants | RT-PCR in nasopharyngeal swab specimens and lung computed tomography scan involvement compatible with COVID-19 pneumonia; body weight > 50 kg; normal levels of serum Ca2+ and K+; QT interval < 450 ms at 12 derivations electrocardiogram (according to the Bazett formula) and negative serum or urinary β-HCG if woman under 50 | Colchicine | 0.5 mg thrice daily for 5 days, then 0.5 mg twice daily for 5 days; if body weight ≥ 80 kg, the first dose was 1.0 mg | Azithromycin 500 mg once daily for up to 7 days; hydroxychloroquine 400 mg twice daily for 2 days, then 400 mg once daily for up to 8 days and unfractionated heparin 5000 UI thrice daily until the end of hospitalization. Methylprednisolone 0.5 mg/kg/day for 5 days could be added if the need for supplemental oxygen was 6 L/min or more. |
|  |  |  | Placebo |  |  |
| Lou 2020 | Zhejiang Provincial Science and technology department key R  & D plan emergency project | COVID-19: positive results of throat swab or blood samples by real-time RT-PCR assay for 2019-nCoV | Baloxavir | 80 mg once a day orally on Day 1 and Day 1 4; for patients who are still positive in virological test, they can be given again on Day 7, no more than three additional doses; | Existing antiviral treatment: lopinavir/ritonavir (400mg/100mg, bid, po.) or darunavir/cobicistat (800mg/150mg, qd, po.) and arbidol (200mg, tid, po.). All of them were used in combination with interferon-α inhalation (100,000 iu, tid or qid) |
|  |  |  | Favipiravir | 1600 mg or 2200mg orally, followed by 600 mg each time, three times a day, and the duration of administration was not more than 14 days |  |
|  |  |  | Existing antiviral treatment |  |  |
| Maldonado 2020 | NR | Confirmation of SARS-CoV-2 infection by viral PCR, clinical symptoms and signs such as fever, fatigue, dry cough, anorexia, myalgia, dyspnea, sputum production, dysgeusia, and anosmia, and infiltrates in chest imaging studies (plain radiography or tomography). | Pentoxifylline | 400 mg every 8 h from admission to discharge | NR |
|  |  |  | Standard care |  |  |
| Mansour 2020 | Public grants provided by the University of Campinas and Sao Paulo Research Foundation | Symptom duration of 12 days or less at recruitment, diagnosis of SARS-CoV-2 by RT-PCR method according to Berlin-Charité 56 protocol, diagnosis of typical COVID-19 pneumonia confirmed by chest CT scan and marked by two expert pulmonary radiologists, SpO2 ≤ 94% in ambient air or Pa02 / FiO2 ≤ 300 mmHg | Icatibant | 30 mg (3.0 ml of 10 mg/ml solution) subcutaneous injections in the abdominal area were administered at intervals of 8 h for 4 days | Upon medical  decision, patients received antibiotics, antithrombotic therapy, oxygen support, non-invasive  and invasive mechanical ventilation, vasopressor drugs, stress doses of  corticosteroids and renal support therapy |
|  |  |  | Inhibitor of C1 esterase/kallikrein | 20 IU / kg body weight on day 1 shortly after recruitment and on day 4 (each vial contains 500 IU of C1 esterase / kallikrein inhibitor as a lyophilized product for reconstitution with 10 ml of sterile water for injection |  |
|  |  |  | Standard care |  |  |
| Mehboob 2020 | NR | NR | Aprepitant plus corticosteroid (dexamethasone) | 80 mg tablets 1 in a day | NR |
|  |  |  | Corticosteroide (dexamethasone) | 20 mg |  |
| Miller 2020 | CalciMedica, Inc. (La Jolla, CA, USA) | RT-PCR and pneumonia from chest imaging. In addition, ≥ 1 symptom such as: fever, cough, sore throat, malaise, headache, muscle pain, dyspnoea, confusion, or difficulty breathing, and ≥ 1 clinical sign suggesting respiratory compromise (respiratory rate ≥ 30 breaths per minute, cardiac ≥ 125 bpm, SpO2 <93% on ambient air or requiring> 2 L of oxygen from nasal cannula to maintain SpO2 ≥ 93%, or PaO2 / FiO2 <300.)  Severe: receiving low-flow supplemental oxygen  Critical: receiving high-flow supplemental oxygen  through a high-flow nasal cannula | Auxora^a^ | Initial dose 2.0 mg/kg (max 250 mg), and subsequent doses were 1.6 mg/kg (max 200 mg) at 24 and 48 h | Antiviral agents, but investigational therapies and immunosuppressive medications were not permitted. At the discretion of the site investigators, patients treated with either Auxora or standard of care alone were able to receive convalescent plasma if they required invasive mechanical ventilation. |
|  |  |  | Standad care^a^ |  |  |
| Mitjà O, 2020 | Crowdfunding campaign JoEmCorono (https://www.yomecorono.com/) and Laboratorios Rubió, Laboratorios Gebro Pharma, Zurich Seguros, SYNLAB Barcelona, and Generalitat de Catalunya. Laboratorios Rubió also contributed to the study with the required doses of hydroxychloroquine (Dolquine®). | Mild symptoms of Covid-19 (e.g. fever, acute cough, shortness of breath, sudden loss of smell or taste or flu-like illness) for less than 5 days prior to enrolment, not hospitalized positive for SARS-CoV-2 PCR test with nasopharyngeal swab | Hydroxychloroquine | 800 mg per day 1, followed by 400 mg 1 time per day for 6 days | NR |
|  |  |  | Standard Care |  |  |
| Monk 2020 | Synairgen Research | Adults aged 18 years or older, admitted to hospital with COVID-19 symptoms. Confirmed SARS-CoV-2 test result in a UK National Health Service (NHS)  diagnostic, qualitative RT-PCR assay or a positive point of-care test (FebriDx, Lumos Diagnostics, Sarasota, FL, USA) within the previous 24 h | Inhaled nebulised interferon beta-1a  (SNG001) | 6 MIU interferon beta-1a delivered via the I-neb nebuliser (Philips Respironics,  Murrysville, PA, USA) once daily for up to 14 days | NR |
|  |  |  | Placebo |  |  |
| Morteza 2020 | Research deputy of Qazvin University of  Medical Sciences and Science and Technology Park, Qazvin, Iran | Clinical symptoms of COVID-19 pneumonia: cough (with or without sputum), fever, pleuritic chest pain or dyspnea; mild to severe COVID-19 disease confirmed by chest computed tomography (CT) scan findings compatible with COVID-19 or positive RT-PRC test | Ivermectin single dose | 400mcg/Kg, 2 pills per day | Standard care: Hydroxychloroquine 200mg/kg twice per day is considered Iran health ministry Standard Care |
|  |  |  | Ivermectin single dose | 200mcg/Kg, 2 pills per day |  |
|  |  |  | Ivermectin three high doses | 400, 200, 200 mcg/Kg, 4 pills in 1, 3 and 5 interval days |  |
|  |  |  | Ivermectin three low doses | 200, 200, 200 mcg/Kg , 3  pills in 1, 3 and 5 interval days |  |
|  |  |  | Standard care |  |  |
|  |  |  | Placebo |  |  |
| Nojomi 2020 | University of Medical Sciences. Iran | Positive for COVID-19 by RT-PCR or computed  tomography (CT) scan imaging (pneumonia), and oxygen saturation of 94% or less. | Umifenovir plus Hydroxychloroquine | 400 mg Hydroxychloroquine, twice daily on first day, followed by 200 mg Umifenovir, 3 times daily, for 7 to 14 days, based on the severity of disease. | NR |
|  |  |  | Lopinavir/Ritonavir plus Hydroxychloroquine |  |  |
| Omrani 2020 | Internal institutional funds of the Hamad Medical Corporation (government  health service of the State of Qatar). | SARS-CoV-2 PCR-positive  males and females with mild or no symptoms. Were excluded patients with the following characteristics: hospitalization, tachypnoea (respirations >29/minute), or hypoxemia (pulse oximetry on room air <93%); treatment was also recommended for any patient with chest X-ray abnormality who had risk factors of older age (>60), immunocompromise,  or co-morbidity (e.g. diabetes or hypertension). | Hydroxychloroquine plus azithromycin | Hydroxychloroquine: 600 mg day-one dose, 600 mg subsequent daily dose  Azithromycin: 500 mg day one,  250 mg daily on days two through five | NR |
|  |  |  | Hydroxychloroquine | Hydroxychloroquine: 600 mg day-one dose, 600 mg subsequent daily dose |  |
|  |  |  | Placebo |  |  |
| Pan (SOLIDARITY trial), 2020 | World Health Organization; Gilead Sciences (drug donation) | Diagnosis of COVID-19, not known to have received any study drug, | Remdesivir  Standard care | Intravenous 200 mg on day 0 and 100 mg on days 1 through 9 | Local standard of care |
|  |  |  | Hydroxychloroquine  Standard care | Oral four tablets at hour 0, four tablets at hour 6, and, starting at hour 12, two tablets twice dailyfor 10 days, each tablet contained 200 mg |  |
|  |  |  | Lopinavir-Ritonavir  Standard care | Oral two tablets twice daily for 14 days, each tablet contained 200 mg |  |
|  |  |  | Interferon β 1a  Standard care | Mainly subcutaneous three doses over a period of 6 days (the day of randomization and days 3 and 6) of 44 μg. Where intravenous interferon was available, patients receiving high-flow oxygen, ventilation, or extracorporeal membrane oxygenation (ECMO) were instead to be given 10 μg intravenously daily for 6 days |  |
| Rahmani 2020 | No fund | Positive PCR and clinical symptoms/signs of pneumonia (including dyspnea, cough and fever), SpO2 ≤ 93% in ambient air or PaO2/FiO2 < 300 or SPO2/FiO2 < 315 and lung involvement in chest  imaging | Interferon β 1b | 250 mcg subcutaneously every other day for two consecutive weeks. | Lopinavir/ritonavir (400/100 mg BD) or atazanavir/ritonavir (300/100 mg daily) plus hydroxychloroquine (400 mg BD in first day and then 200 mg BD) for7–10 days.  Other supportive cares such as fluid therapy, stress ulcer prophylaxis, deep vein thrombosis, treatment of electrolyte disorders and antibiotic therapy were considered according to the hospital protocols. |
|  |  |  | Standard care |  |  |
| Recovery Trial | University of Oxford from UK Research and Innovation/National Institute for Health Research (NIHR) and by core funding provided by NIHR Oxford Biomedical Research Centre, Wellcome,  the Bill and Melinda Gates Foundation, the Department for International Development, Health Data Research UK, the Medical Research Council Population Health Research Unit, and NIHR Clinical Trials Unit Support Funding ritonavir for use in the trial. Other medications, including  dexamethasone, that were used in the trial were supplied by the  National Health Service (NHS). | SARS-CoV-2 infection (clinically suspect or laboratory confirmed) | Hydroxychloroquine  Standard Care | 4 tablets: 800 mg at 0 and 6 hours then 2 tablets: 400 mg 12 hours after the initial dose every 12 hours over the following 9 days. | Usual local hospital care. |
|  |  |  | Dexamethasone  Standard care | oral or intravenous 6mg 1 time a day for 10 days |  |
|  |  |  | Lopinavir/Ritornavir  Standard care | 400mg/100mg every 12 hours |  |
|  |  |  |  |  |  |
|  |  |  |  |  |  |
|  |  |  |  |  |  |
|  |  |  |  |  |  |
| REMAP-CAP trial 2020 | Platform for European Preparedness Against (Re-) emerging Epidemics (PREPARE) consortium by the European Union, FP7-HEALTH-2013-INNOVATION-1, the Australian National Health and Medical Research Council, the  New Zealand Health Research Council,  the Canadian Institute of Health Research  Strategy for Patient-Oriented Research Innovative  Clinical Trials Program, the UK  National Institute for Health Research (NIHR) and  the NIHR Imperial Biomedical Research Centre, the  Health Research Board of Ireland, the UPMC Learning While Doing  Program, the Breast Cancer Research Foundation,  the French Ministry of Health, and the Minderoo Foundation. | Severe patients as: patients admitted to an intensive care unit (ICU) for provision of respiratory or cardiovascular organ support. | Corticosteroid (Hydrocortisone)_fixed dose | Intravenous 50 mg, every 6 hours for 7 days | All patients admitted to an ICU with severe CAP different component therapies: treatment of the underlying infection (including antibacterial and antiviral agents); the optional use of agents, such as  corticosteroids, that modulate the host immune response to infection; and multiple supportive therapies that are used to manage organ systems that have failed or prevent complications of critical illness and its treatment.  In addition to assignment to interventions in the corticosteroid domain, participants could be randomly assigned to other interventions within other therapeutic domains, depending on whether the site was active for that domain, patient eligibility |
|  |  |  | Corticosteroid (Hydrocortisone)_shock-dependent | Intravenous 50 mg, every 6 hours while in shock for up to 28 days |  |
|  |  |  | Standard care |  |  |
| Ren 2020 | Henan Genuine Biotech Co. | SARS-CoV-2 positive RT-PCR, or blood or respiratory samples that have been tested highly coincident with SARS-CoV-2 by viral gene sequencing; the confirmation of COVID-19 according to the diagnostic criteria of the "latest clinical guidelines for new coronaviruses" published by the World Health Organization (WHO) on January 28, 2020, and the diagnostic criteria of the "Diagnosis and treatment program trial version 5 (or 6) guidelines "published by the National Health Commission of the People's Republic of China Laboratory test and CT images | Azvudina | 1 tablet of 5 mg/day | Standard antiviral drugs: interferon alpha, kaletra and ribavirin and interferon alpha, kaletra and ribavirin, chloroquine phosphate and hydroxychloroquine sulfate  Symptomatic treatment: antibiotic therapy and traditional Chinese medicine |
|  |  |  | Standard Care |  |  |
| Rocco 2020 | Brazilian Council for Scientific and Technological Development  (CNPq), Brazilian Ministry of Science, Technology, and Innovation for Virus Network. Brasil | Positive for SARS-CoV-2 infection by RT-PCR test and clinical symptoms of Covid-19 (defined for the purposes of this trial as dry cough, fever, and/or fatigue) of no longer than 3 days’ duration | Nitazoxanide | 500 mg oral solution, 20 mg/mL [25 mL],  three times daily for 5 days | NR |
|  |  |  | Placebo |  |  |
| Rosas 2020 | Roche/Genentech; National Institutes of Health; Merck and Livanova | Severe COVID-19 pneumonia confirmed by positive PCR test in any body fluid and evidenced by bilateral chest infiltrates on chest x-ray or computed tomography were enrolled. Blood oxygen saturation ≤93% or partial pressure of oxygen/fraction of inspired oxygen <300 mm/Hg. | Tocilizumab | Intravenous 8 mg/kg infusion, maximum 800 mg. If clinical signs or symptoms did not improve or worsened a second infusion could be administered 8 to 24 hours after the first. | Antiviral treatment, low-dose steroids, convalescent plasma, supportive care. |
|  |  |  | Placebo |  |  |
| Sadeghi 2020 | Internal funding of the Digestive Disease  Research Institute of Tehran University of Medical Sciences. | Positive qualitative RT–PCR for SARS-CoV-2 and/or features consistent with COVID-19 on a chest CT scan. Additionally, signs of disease severity defined as fever (oral temperature 37.8C at any time prior to enrolment) and at least one of respiratory rate> 24 / min, O2 saturation <94%, or PaO2 / FiO2 ratio <300 mgHg | Sofosbuvir | Single daily oral tablet containing 400mg sofosbuvir and 60mg daclatasvir | Hydroxychloroquine 200mg twice daily with or without lopinavir/ritonavir  200mg/50mg twice daily. |
|  |  |  | Standard Care |  |  |
| Sakoulas 2020 | Supported, in part, by Octapharma USA, Hoboken, NJ | Positive for COVID-19 infection confirmed by PCR assay and moderate-to-severe hypoxia (sPo2 ≤ 96% on ≥ 4 L O2 by nasal cannula) but not on mechanical ventilation | Immunoglobulin | 0.5-g/kg IV Octagam 10% , daily for 3 days plus with 40 mg methylprednisolone,  30 minutes before infusion for 3 days | One of two IVIG subjects and two of seven control subjects also received concomitant convalescent plasma. Concomitant glucocorticoid therapy was given to five of the seven control subjects who progressed to mechanical ventilation.  Remdesivir was given to one of two IVIG patients and three of seven SOC subjects that required mechanical ventilation |
|  |  |  | Standard care | Glucocorticoids, convalescent plasma, and remdesivir or other treatments not included in the trial. |  |
| Salama 2020 | Genentech | Positive for SARS CoV-2 confirmed by polymerase-chain-reaction test and pneumonia confirmed by radiographic imaging. | Tocilizumab | One or two doses of either intravenous tocilizumab (8 mg per kilogram of body weight, to a maximum of 800 mg per dose) | All patients received standard care according to local practice which could include antiviral treatment, the limited use of systemic glucocorticoids (recommended dose, ≤1 mg per kilogram of body weight of methylprednisolone or equivalent), and supportive care |
|  |  |  | Placebo |  |  |
| Salvarani 2020 | Local resources, the Italian Ministry of Health and Roche | COVID-19 pneumonia confirmed by a positive RT-PCR assay for SARS-CoV-2 in a respiratory tract specimen. A PaO2/FIO2 ratio between 200 and 300 mm/Hg, an inflammatory phenotype defined by a temperature greater than 38 °C during the last 2 days, and/or serum C-reactive protein (CRP) levels of 10mg/dL or greater and/or CRP level increased to at least twice the admission measurement | Tocilizumab | Intravenous within 8 hours from randomization at a dose of 8mg/kg up to a maximum of 800 mg, followed by a second dose after 12hours. | Supportive care following the treatment protocols of each center including hydroxychloroquine, heparin and LMWH, antiretrovirals (darunavir/cobicistat, darunavir/ritonavir, or lopinavir/ritonavir. No remdesivir was administered), azithromycin |
|  |  |  | Standard care |  |  |
| Sekhavati 2020 | Tehran University of Medical Sciences’ research center | Reverse-transcriptase–polymerase-chain-reaction (RT-PCR) testing, and a lung CT-Scan.  The inclusion criteria were a positive RT-PCR test and significant findings compatible with radiographic imaging of a COVID-19 pulmonary involvement | Azytromicin plus Lopinavir/Ritonavir plus Hydroxychloroquine | 500 mg, twice-daily oral lopinavir/ritonavir 400/100 mg, and daily 400 mg of oral hydroxychloroquine. | NR |
|  |  |  | Lopinavir/Ritonavir plus Hydroxychloroquine | twice-daily oral lopinavir/ritonavir 400/100 mg, and oral daily 400 mg of hydroxychloroquine |  |
| Self 2020 | National Heart, Lung, and Blood Institute (NHLBI), NCATS, Harvard Catalyst, Sandoz | Symptoms of acute respiratory infection, one or more of : cough, fever (> 37.5° C / 99.5° F), shortness of breath (operationalized as any of the following: subjective shortness of breath reported by patient or surrogate; tachypnea with respiratory rate ≥22 /minute; hypoxemia, defined as SpO2 <92% on room air, new receipt of supplemental oxygen to maintain SpO2 ≥92%, or increased supplemental oxygen to maintain SpO2 ≥92% for a patient on chronic oxygen therapy), sore throat; Laboratory-confirmed SARS-CoV-2 infection within the past 10 days prior to randomization | Hydroxychloroquine | 400mg in pill form twice a day for the first 2 doses and then 200 mg in pill form twice a day for the subsequent 8 doses, for a total of 10 doses over 5days | Receipt of Concomitant Medications After Randomization During Hospitalization: antivirals (open label hydroxychloroquine, remdesivir, others) n=66 (27%); immunomodulator (cotticosteroids, tocilizumab, other) n=50 (20%); antibacterial (azithromycin) n=47 (19%); convalescent plasma n=19 (8%)  Receipt of Concomitant Medications After Randomization During Hospitalization: antivirals (open label hydroxychloroquine, remdesivir, others) n=56 (23%); immunomodulator (cotticosteroids, tocilizumab, other) n=69 (29%); antibacterial (azithromycin) n=44 (19%); convalescent plasma n=25 (11%) |
|  |  |  | Placebo | same volume of hyrdoxychloroquine group |  |
| Shi 2020 | The National Key R&D Program of China; The Innovation Groups of the National Natural Science Foundation of China; The National Science and Technology Major Project | severe COVID-19 with laboratory-confirmed SARS-CoV-2 143 infection by RT-PCR, 1) severe COVID-19 diagnosed after onset of disease; chest computed tomography (CT) imaging confirmed pneumonia combined with lung damage.  Severe (Guidelines issued by the National Health Commission of China version 7.0: 1) dyspnoea (respiratory rate ≥ 30 times/min); 2) oxygen saturation of 93% or lower on room air; 3) PaO2/FiO2 ≤ 300 mmHg; 4) pulmonary imaging showing that the foci progressed by > 50% in 24-48 hours. | Umbilical cord_ mesenchymal stem cells (hUC-MSC) | 4.0 × 10^7^ MSCs in a volume of 100 ml/bag | According to the “Chinese Clinical Guidance for COVID-19  Pneumonia Diagnosis and Treatment (7th edition)” compiled by the National Health Commission of China |
|  |  |  | Placebo |  |  |
| Shu 2020 | The National Natural Science  Foundation of China and the Key Research and Development  Project of Jiangsu Province | Based on WHO interim guidance and a new coronavirus pneumonia diagnosis and treatment program (6th ed.) (in Chinese). Severe: (A) an epidemiological history; (B) etiological evidence (i.e., a positive SARS-CoV-2 nucleic acid test by the RT-PCR assay for SARS-CoV-2 RNA from the Chinese Center for Disease Control and Prevention; and (C) CT imaging indicators of pneumonia. In addition, these factors should coincide with any of the following criteria: (a) respiratory distress, respiration rate ≥ 30 times/min; (b) oxygen saturation ≤ 93% in the resting state; and (c) PaO2/FiO2 ≤ 300 mmHg (1 mmHg = 0.133 kPa). | Umbilical cord_ mesenchymal stem cells (hUC-MSC) | Suspended in 100 ml of normal saline, and the total number of transplanted cells was calculated as 2 × 10^6^ cells/kg. The infusion was from the patients’ right cubital veins and lasted approximately 1 h (35 drops/min). | (1) supplemental oxygen (noninvasive or invasive ventilation), (2) antiviral agents (abidor/oseltamivir), (3) antibiotic agents (moxifloxacin is taken orally; if there is clear evidence of bacteriological infection, the choice of antibacterial drugs is based on a drug sensitivity test), a (4) glucocorticoid therapy (1–2 mg/kg, less than a week). |
|  |  |  | Standard care |  |  |
| Spinner 2020 | Gilead Sciences Inc. | SARS-CoV-2 infection confirmed by PCR assay within 4 days before randomization.  Moderate COVID-19 pneumonia: any radiographic evidence of pulmonary infiltrates and oxygen saturation >94% on room air | Remdesivir 5 days | Remdesivir intravenous 200mg on day 1 and 100mg on the following days | According to local practice |
|  |  |  | Remdesivir 10 days |  |  |
|  |  |  | Standard Care |  |  |
| Stone 2020 | Genentech | Confirmed SARS-CoV-2 infection by nasopharyngeal swab PCR or serum assay for IgM antibody. Requiring hospital but not mechanical ventilation Oxygen supplementation not greater than 10L delivered by any device.  Severe COVID- 19 (at least 2 of the following): Fever > 38C within 72 hours, Unable to provide verbal informed consent or have verbal agreement to participate through attestation and signature of a  Need for supplemental O2 to maintain saturation > 92% AND at least 1 of the following:  • Ferritin > 500 ng/ml  • CRP > 50 mg/L  • LDH >250 U/L  • D-dimer > 1000 ng/mL | Tocilizumab | Intravenous 8mg/kg (800 mg max) | Patients received the standard treatment for COVID-19 Massachusetts General Hospital (MGH) guidance: details NR |
|  |  |  | Placebo |  |  |
| Tang 2020 | Shanghai Pharmaceuticals Holding Co.,Ltd, R&G  PharmaStudies Co., Ltd. | SARS-CoV-2 infection confirmed in upper or lower respiratory tract specimens with RT-PCR | Hydroxychloroquine | 1200mg for 3 days, followed by 800mg | Standard Care: according to "the updating National clinical practice guidelines for COVID-19 in China n=37 (49%), virazole n=13 (17%), lopinavir/ritonavir n=13 (17%), oseltamivir n=8 (11%), entecavir n=1 (1%), antibiotics n=32 (43%), glucocorticoid n=6 (8%) |
|  |  |  | Standard Care |  | n=33 (44%), virazole n=15 (20%), lopinavir/ritonavir n=12 (16%), oseltamivir n=9 (12%), entecavir n=1 (1%), antibiotics n=27 (36%), glucocorticoid n=4 (5%) |
| Tomazini 2020 | Coalition COVID-19 Brazil. The  Laboratórios Farmacêuticos provided the study  drug, distribution logistics, and insurance for the  study patients. | Confirmed or suspected COVID-19 infection. Due to the possibility of false negative tests associated, the committee took into account the epidemiology (travel or residence in a city where community transmission is reported or contact with a confirmed case in the last 14 days prior to symptoms onset), timing of testing from symptoms’ onset, clinical symptoms and analysis of chest image (computed tomography scan of the lungs, or chest X-ray) | Corticosteroid (Dexamethasone) | 20mg intravenously once daily for 5 days, followed by 10 mg intravenously once daily for additional  5 days or until ICU discharge, whichever occurred first. | Following best practice guidelines and their institutional protocol for the care of critically ill patients with COVID-19. |
|  |  |  | Standard care |  |  |
| Ulrich 2020 | National Institutes of Health, National Center for Advancing Translational Sciences, New York State Department of Health. | Positive SARS-CoV-2 reverse transcriptase polymerase chain reaction (RT-PCR), at least one COVID-19 symptom | Hydroxychloroquine | Hydroxychloroquine sulfate 200mg tablets | NR |
|  |  |  | Placebo | Calcium citrate 200mg tablets |  |
| Vlaar 2020 | InflaRx GmbH | Severe pneumonia with pulmonary infiltrates consistent with pneumonia or a clinical history of severe shortness of breath within the past 14 days,  or need for non-invasive or invasive ventilation;  and had a PaO2/FiO2 ratio between 100 and 250 mmHg in supine position; and PCR confirmed SARS-CoV-2 infection. | Anti-C5a antibody (IFX-1) | 800 mg intravenously, for a maximum of seven doses. Five treatments (day 1, 2, 4, 8, and 15) were administered to all patients randomized to the IFX-1 group alive and hospitalized. Treatment at day 22 was administered in patients who were still intubated at time of administration. One additional administration of IFX-1 could be given between day 11 and 13 at investigator discretion if signs of weakening of any clinical improvement  were detected. Treatment with IFX-1 was discontinued if patients were discharged from the hospital | Best supportive care: intensive care therapy according to current guidelines, evidence and best practice including but not limited to lung protective ventilation, thrombosis prophylaxis, renal replacement therapy when indicated and access to advanced therapies including extra corporal membrane oxygenation (ECMO). |
|  |  |  | Best supportive care |  |  |
| Wang 2020 | Gilead Sciences Inc. | SARS-CoV-2 confirmed by RT-PCR positive, pneumonia confirmed by chest imaging, oxygen saturation of 94% or less or a PaO2/FiO2 ratio of 300 mm Hg or less, within 12 days of symptom onset. | Remdesivir | 200 mg on day 1 followed by 100 mg on days 2-10 in single daily infusions. | Other treatments before and after enrollement Interferon alpha-2b (29%); lopinavir-ritonavir (28%); vasopressin (16%); Renal replacement therapy (2%); Non-invasive mechanical ventilation (9%); Invasive mechanical ventilation (7%); ECMO or mechanical ventilation (1%); Antibiotics (90%); Corticosteroids (65%) |
|  |  |  | Placebo |  | Other treatments before and after enrollement Interferon alpha-2b (38% ); lopinavir-ritonavir (29%); vasopressin (17%); Renal replacement therapy (4%); Non-invasive mechanical ventilation (4%); Invasive mechanical ventilation (13%); ECMO or mechanical ventilation (0%); Antibiotics (94%); Corticosteroids (68%) |
| Wang D 2020 | Department of Science and Technology of Anhui Province and Health Commission of Anhui Province, China National Center for Biotechnology Development. | RT-PCR positive for SARS-CoV-2.  Moderate or severe degree was defined according to the “Diagnosis and Treatment Protocol for Novel Coronavirus Pneumonia (5th or update version).  Moderate: fever or other respiratory symptoms, bilateral pulmonary lesions confirmed by chest imaging.  Severe: respiratory rate ≥30 breaths per min; SpO2 ≤ 93% while breathing room air; PaO2/FiO2 ≤ 300 mm Hg | Tocilizumab | First dose of 400 mg, diluted in 100 ml 0.9% saline, and intravenous dripped  in more than 1 h. A second dose was given if a patient remained febrile for 24 hours after the first dose. | According to the “Diagnosis and Treatment Protocol for Novel Coronavirus Pneumonia (5th or update version)”. |
|  |  |  | Standard care |  |  |
| Wang M 2020 | National Key Research and Development Plan for the Emergency Management of Novel Coronavirus Pneumonia,China and the Wuhan Municipal Key Technology Project on Novel Coronavirus Pneumonia, China | Diagnosis of COVID-19 conforming to the Chinese Guidelines | Leflunomide plus IntInterferon α-2aerferon α-2a | Leflunomide 50 mg, q12h, three consecutive times, orally; then 20 mg, once a day for 8 days; a total course of 10 days plus nebulized.  Interferon α-2a 3 million IU each time, adding 2 ml of sterilized water, atomization inhalation twice daily for 10 days | NR |
|  |  |  | Interferon α-2a |  |  |
| Wu 2020 | Chinese Academy of Engineering Projects for COVID-19 (2020-KYGG-01-04) and Heilongjiang Province Urgent Project-6 for COVID-19. | Laboratory-confirmed SARS-CoV-2 infection by  RT-PCR; chest computed tomography (CT) imaging-confirmed lung damage—namely, multiple small plaques and stromal changes in the lungs, manifested in the outer lung, or with multiple ground glass shadows and infiltration shadows in both lungs hospitalized patients with fever (axillary temperature ≥ 37.0 °C) or respiratory symptoms; a time from symptom onset to randomization of less than 12 d; | Triazavirin | Mild patients: 250 mg orally three times a day for seven consecutive days;  severe patients: 250 mg orally four times a day for seven consecutive days. | Concurrent treatments:  Antimicrobial drugs, Antiviral drugs, Interferon, Arbidol, Ribavirin, Lopinavir, Antibacterial drugs, Hydroxychloroquine, Antifungal drugs, Chinese medicine therapy |
|  |  |  | Placebo |  |  |
| Yueping 2020 | Project 2018ZX10302103-002, 2017ZX10202102-003-004 and Infectious Disease Specialty of Guangzhou High-level Clinical Key Specialty (2019-2021) | SARS-CoV-2 infection confirmed by RT-PCR from pharyngeal swab;  Mild: mild clinical symptoms but no signs of pneumonia on imaging or moderate clinical status, defined as having fever, respiratory symptoms and pneumonia on imaging | Lopinavir–ritonavir | lopinavir 200mg, ritonavir 50mg: orally, q12h, 500 mg each time for 7-14 days | Supportive care and effective oxygen therapy if in need. Any antiviral therapy |
|  |  |  | Umifenovir | 100mg orally: 200mg TID for 7-14 days. |  |
|  |  |  | Standard Care |  |  |
| Zheng 2020 | Public: National Science and Technology Major Project, the National Key Research and Development  Program of China, and Specialized Science and Technology  Project of Hunan Province. | SARS-CoV-2 confirmed by RT-PCR positive Moderate: fever, symptoms of respiratory system changes and pneumonia in CT images. Severe: at least one symptom such as:respiratory distress, respiratory rate ≥30/minute; at rest, arterial oxygen saturation (SaO2)≤ 93%; PaO2/ FiO2≤ 300mmHg. | Novaferon | 40 μg total per day administered twice daily by atomised oxygen inhalation for 15 minutes of 20 μg (2 ml vials) diluted with saline | NR |
|  |  |  | Novaferon plus Lopinavir/Ritonavir | Novaferon: 40 μg total per day administered twice daily by atomised oxygen inhalation for 15 minutes of 20 μg (2 ml vials) diluted with saline; Lopinavir/Ritonavir: 2 tablets daily for a total of 200 mg |  |
|  |  |  | Lopinavir/Ritonavir | 2 tablets daily for a total of 200 mg |  |

RT-PCR: Reverse transcriptional polymerase chain reaction, CT: computed tomography Pa2O: arterial partial pressure of oxygen; FIO2: /fraction of inspired oxygen; SpO2: peripheral oxygen saturation; *: convalescent Plasma; NR: not reported; n: number of participants; a: 26 patients received low flow supplemental oxygen (17 assigned to Auxora, 9 assigned to SC) and 4 patients received high flow supplemental oxygen (3 assigned to Auxora, 1 assigned to SC)

### **Appendix 5. Evaluation of the transitivity assumption.**

**Table S2. Evaluation of the transitivity assumption for the main effect modifiers across trial comparisons**. The following characteristics have been evaluated in all trials included in the network irrespectively of the outcome being reported: Age, gender, disease severity (mild to moderate, severe, critical).

Most of the comparisons had similar mean age and gender but there were a few comparisons which had different disease severity.

| **COMPARISON** | **N of comparisons studied in RCTs (%) (n= 96)** | **Peer reviewed**  **% (n =96)** | **EFFECT MODIFIERS** | | | | |
| --- | --- | --- | --- | --- | --- | --- | --- |
|  |  |  | **Gender (n= 94)** | **Age (n= 94)** | **Disease severity (n= 88)*** | | |
|  |  |  | **Males %**  **[95% CIs]** | **Mean age**  **[95% CIs]** | **Mild to moderate % [95% CIs]** | **Severe % [95% CIs]** | **Critical %**  **[95% CIs]** |
| Anti-C5a_(IFX-1) vs. SC | 1 (0.84) | 0 (0) | 73 | 60.5 |  |  |  |
| Aprepitant+Corticosteroid vs. Corticosteroid | 1 (0.84) | 0 (0) | 61.1 | 55 |  |  |  |
| Auxora vs. SC | 1 (0.84) | 1 (100) | 46.7 | 59.3 | 0 | 100 | 0 |
| Azithromycin vs. Hydroxychloroquine | 1 (0.84) | 1 (100) | 61.6 | 54.5 |  |  |  |
| Azithromycin vs. Lincomycin | 1 (0.84) | 1 (100) | 62.5 | 58.8 | 100 | 0 | 0 |
| Azithromycin+Lopinavir/Ritonavir+Hydroxychloroquine vs. Lopinavir/Ritonavir+Hydroxychloroquine | 1 (0.84) | 1 (100) | 45.9 | 57.1 |  |  |  |
| Azvudine vs. SC | 1 (0.84) | 1 (100) | 60 | 51.2 |  | 0 | 0 |
| Baloxavir vs. Favipiravir | 1 (0.84) | 0 (0) | 73.5 | 55.8 |  |  |  |
| Baloxavir vs. SC | 1 (0.84) | 0 (0) | 70 | 50 |  |  |  |
| Bromhexine vs. SC | 2 (1.68) | 2 (100) | 66.4 [50.5,82.4] | 55.4 [49.2,61.5] |  | 0 | 0 |
| Calcifediol vs. SC | 1 (0.84) | 1 (100) | 59.1 | 53.3 |  |  | 0 |
| Chloroquine vs. Hydroxychloroquine | 1 (0.84) | 0 (0) | 41.8 | 45.5 | 100 | 0 | 0 |
| Chloroquine vs. Lopinavir+Ritonavir | 1 (0.84) | 1 (100) | 40.9 | 47.8 | 63.6 | 36.4 | 0 |
| Chloroquine vs. SC | 1 (0.84) | 0 (0) | 45.9 | 47.4 | 100 | 0 | 0 |
| Colchicine vs. SC | 1 (0.84) | 1 (100) | 58.5 | 64 |  |  |  |
| Colchicine vs. SC/placebo | 1 (0.84) | 0 (0) | 40.4 | 50.5 |  |  | 0 |
| Corticosteroid vs. SC | 5 (4.2) | 4 (80) | 62.1 [60.8,63.3] | 63.2 [59.6,66.9] | 4.8 [0,13.5] | 75 [31.4,118.6] | 25 [0,68.5] |
| Corticosteroid vs. SC/placebo | 2 (1.68) | 2 (100) | 67.6 [64.4,70.8] | 59.8 [53.0,66.7] | 0 |  |  |
| Darunavir+Cobicistat vs. SC | 1 (0.84) | 1 (100) | 60 | 47.2 | 100 | 0 | 0 |
| Favipiravir+Interferon β 1b vs. Hydroxychloroquine | 1 (0.84) | 1 (100) | 0 | 55 |  |  | 0 |
| Favipiravir vs. Favipiravir+Tocilizumab | 1 (0.84) | 1 (100) | 52.4 | 73.3 |  |  |  |
| Favipiravir vs. SC | 5 (4.2) | 2 (40) | 58.9 [48.2,69.5] | 44.9 [39.6,50.2] | 100 | 0 | 0 |
| Favipiravir vs. Tocilizumab | 1 (0.84) | 1 (100) | 66.7 | 70.4 |  |  |  |
| Favipiravir vs. Umifenovir | 1 (0.84) | 0 (0) | 46.6 | 0 | 88.6 | 11.4 | 0 |
| Favipiravir+Tocilizumab vs. Tocilizumab | 1 (0.84) | 1 (100) | 47.4 | 73.9 |  |  |  |
| Fluvoxamine vs. SC/placebo | 1 (0.84) | 1 (100) | 28.1 | 45.5 |  |  |  |
| Frozen fresh plasma vs. convalescent Plasma | 1 (0.84) | 0 (0) | 75.9 | 48.2 | 0 | 0 | 100 |
| Human Granulocyte Colony (rhG-CSF) vs. SC | 1 (0.84) | 1 (100) | 56 | 45.5 |  |  |  |
| Hydroxychloroquine vs. Hydroxychloroquine+Azithromycin | 3 (2.52) | 3 (100) | 74.9 [55.7,94.2] | 48.2 [42.2,54.2] | 100 | 0 | 0 |
| Hydroxychloroquine vs. Hydroxychloroquine+Zinc | 1 (0.84) | 1 (100) | 60.8 | 43.6 | 70.2 | 19.9 | 9.9 |
| Hydroxychloroquine vs. SC | 14 (11.76) | 8 (57.1) | 53.6 [44.9,62.3] | 50.2 [43.3,57.1] | 74.8 [43.8,105.9] | 18.3 [-5.1,41.7] | 0 |
| Hydroxychloroquine vs. SC/placebo | 1 (0.84) | 1 (100) | 98.3 | 40.5 | 100 | 0 | 0 |
| Hydroxychloroquine+Azithromycin vs. Ivermectin+Doxycycline | 1 (0.84) | 0 (0) | 77.8 | 33.8 | 100 | 0 |  |
| Hydroxychloroquine+Azithromycin vs. SC | 1 (0.84) | 1 (100) | 55.4 | 49.8 |  | 0 | 0 |
| Hydroxychloroquine+Azithromycin vs. SC/placebo | 1 (0.84) | 1 (100) | 98.7 | 41.5 | 100 | 0 | 0 |
| Icatibant vs. Inhibitor_of_C1_esterase/kallikrein | 1 (0.84) | 0 (0) | 55 | 53 | 0 | 100 | 0 |
| Icatibant vs. SC | 1 (0.84) | 0 (0) | 60 | 50.2 | 0 | 100 | 0 |
| Immunoglobuline vs. SC | 2 (1.68) | 2 (100) | 68.7 [56.4,80.9] | 54.1 [53.4,54.8] | 0 | 100 | 0 |
| Immunoglobuline vs. SC/placebo | 1 (0.84) | 1 (100) | 69.5 | 55.7 | 0 | 100 | 0 |
| Inhibitor of C1 esterase/kallikrein vs. SC | 1 (0.84) | 0 (0) | 45 | 51.7 | 0 | 100 | 0 |
| Interferon α 2b+interferon γ vs. Interferon α 2b | 1 (0.84) | 0 (0) | 53.4 | 36.7 |  | 0 | 0 |
| Interferon α 2a vs. Leflunomide+ Interferon α 2a | 1 (0.84) | 1 (100) | 46.2 | 55.8 |  |  |  |
| Interferon β 1a vs. SC | 2 (1.68) | 2 (100) | 58.7 [52.6,64.7] | 58 |  |  | 0 |
| Interferon β 1b vs. SC | 2 (1.68) | 2 (100) | 59.1 | 58.8 [56.5,61.2] |  | 65.3 | 0 |
| Itolizumab vs. SC | 1 (0.84) | 0 (0) | 87.2 | 49.2 | 0 | 100 | 0 |
| Ivermectin vs. SC | 2 (1.68) | 0 (0) | 51.7 [46.1,57.3] | 48.3 [37.9,58.7] |  | 5.6 [0,13.5] | 0 |
| Ivermectin+Doxycycline vs. SC | 1 (0.84) | 0 (0) | 52 | 48.7 |  | 15.7 | 7.9 |
| LY-CoV555 vs. SC/placebo | 1 (0.84) | 1 (100) | 45 | 45.3 | 100 | 0 | 0 |
| Lopinavir+Ritonavir vs. Lopinavir+Ritonavir+Ribavirin+Interferon β 1b | 1 (0.84) | 1 (100) | 53.3 | 51.3 |  | 0 | 0 |
| Lopinavir+Ritonavir vs. Novaferon | 1 (0.84) | 1 (100) | 49.2 | 45.7 | 94.9 | 5.1 | 0 |
| Lopinavir+Ritonavir vs. Novaferon+Lopinavir+Ritonavir | 1 (0.84) | 1 (100) | 42.4 | 45 | 94.9 | 5.1 | 0 |
| Lopinavir+Ritonavir vs. SC | 4 (3.36) | 4 (100) | 55.7 [48.7,62.7] | 53.3 [46.6,59.9] | 0 | 100 | 0 |
| Lopinavir+Ritonavir vs. Umifenovir | 1 (0.84) | 1 (100) | 47.8 | 50.6 |  |  |  |
| Lopinavir/Ritonavir+Hydroxychloroquine vs. Umifenovir+Hydroxycloroquine | 1 (0.84) | 0 (0) | 60 | 54.6 | 77 | 23 | 0 |
| Lopinavir/Ritonavir+interferon α vs. Ribavirin+ Interferon α | 1 (0.84) | 1 (100) | 54 | 41.9 | 100 | 0 | 0 |
| Lopinavir/Ritonavir+interferon α vs. Ribavirin+Interferon α+Lopinavir/Ritonavir | 1 (0.84) | 1 (100) | 41.2 | 43.5 | 100 | 0 | 0 |
| Nitazoxamide vs. SC | 1 (0.84) | 0 (0) | 0 | 47 |  | 0 | 0 |
| Novaferon vs. Novaferon+Lopinavir+Ritonavir | 1 (0.84) | 1 (100) | 50 | 49.5 | 93.3 | 6.7 | 0 |
| Peginterferon λ 1a vs. SC/placebo | 1 (0.84) | 0 (0) | 57.5 | 35.5 |  | 0 | 0 |
| Pentoxyfilline vs. SC | 1 (0.84) | 1 (100) | 55.3 | 57.6 |  |  |  |
| Remdesivir vs. SC | 2 (1.68) | 2 (100) | 62.1 [61.0,63.3] | 57 | 100 | 0 | 0 |
| Remdesivir vs. SC/placebo | 2 (1.68) | 2 (100) | 61.7 [57.9,65.5] | 62.1 [57.6,66.7] | 0 | 94.3 [86.1,102.4] | 0 |
| Ribavirin+Interferon α ± vs. Ribavirin+Interferon α +Lopinavir/Ritonavir | 1 (0.84) | 1 (100) | 41.7 | 42 | 100 | 0 | 0 |
| Ruxolitinib vs. SC | 1 (0.84) | 1 (100) | 58.5 | 63.5 | 0 | 100 | 0 |
| SC vs. Sofosbuvir+Daclatasvir | 2 (1.68) | 1 (50) | 47.5 [41.9,53.1] | 54.5 [46.8,62.3] | 82 | 18 | 0 |
| SC vs. Sofosbuvir+Daclatasvir+Ribavirin | 1 (0.84) | 1 (100) | 37.5 | 52.5 | 100 | 0 | 0 |
| SC vs. Telmisartan | 1 (0.84) | 0 (0) | 61.4 | 61.9 |  |  |  |
| SC vs. Tocilizumab | 3 (2.52) | 2 (66.7) | 66.3 [62.0,70.7] | 63 [61.1,64.9] | 0 | 100 | 0 |
| SC vs. Umbilical-cord cells (hUC-MSC) | 1 (0.84) | 1 (100) | 55.7 | 58.8 |  |  | 0 |
| SC vs. Umifenovir | 1 (0.84) | 1 (100) | 44.2 | 48.5 |  |  |  |
| SC vs. convalescent Plasma | 6 (5.04) | 2 (33.3) | 69.3 [62.3,76.3] | 59.6 [54.5,64.6] | 60 [15.5,104.5] | 25 [0,68.6] | 0 |
| SC/placebo vs. Tocilizumab | 3 (2.52) | 1 (33.3) | 62.4 [56.4,68.5] | 58.8 [56.4,61.3] | 0 | 99.6 [99.1,100.2] | 0 |
| SC/placebo vs. Triazavirin | 1 (0.84) | 1 (100) | 50 | 56 |  |  | 0 |
| SC/placebo vs. Umbilical-cord cells (hUC-MSC) | 1 (0.84) | 0 (0) | 56 | 60.3 | 0 | 100 | 0 |
| SC/placebo vs. convalescent Plasma | 1 (0.84) | 1 (100) | 67.6 | 62.3 | 0 | 100 | 0 |
| **Total** | **119 (100)** | **78 (65.5)** | **59 [56.0,62.1]** | **53 [51.2,55.3]** | **54 [38.6,69.4]** | **32.5 [20.2,44.8]** | **2.6 [0,5.9]** |

Legend: * Information on disease severity was missing for 8 studies and was incomplete on 38 studies (i.e. different categories were lumped together, such as moderate to severe, and it was not possible differentiate the percentage of moderate and severe within the trials). CIs = confidence intervals; n = number of studies; RCT = randomised controlled trials; SC = standard care.

### **Appendix 6. Risk of bias assessment**


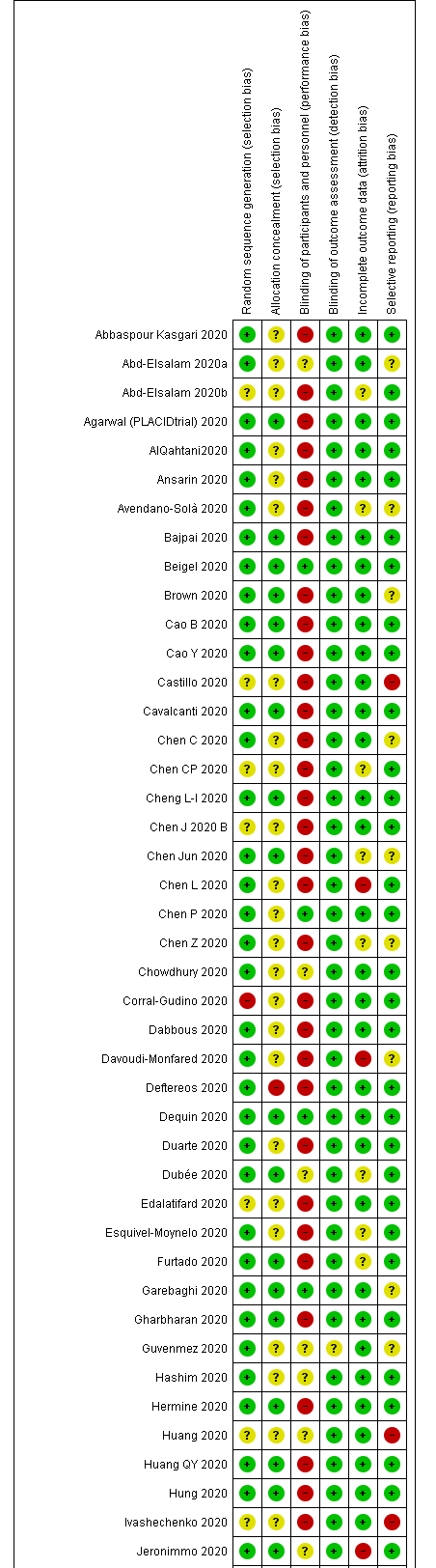
**
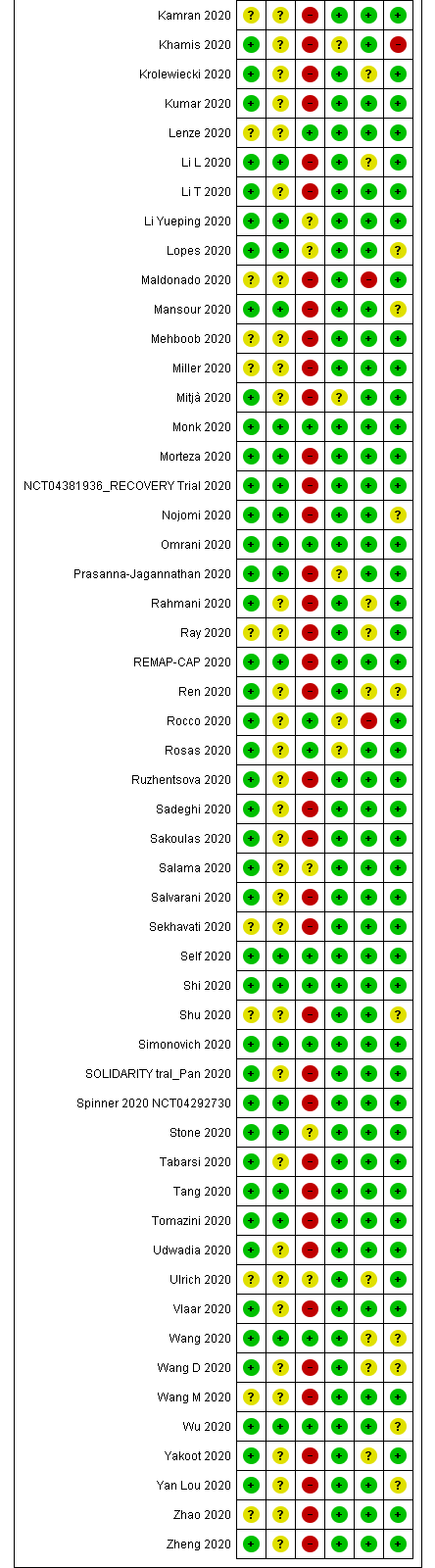
**

Low risk of bias


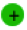


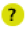
 Unclear risk of bias


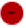
 High risk of bias

**Figure S1**. Risk of bias summary: These are review authors' judgments about each risk of bias item for each included study

**
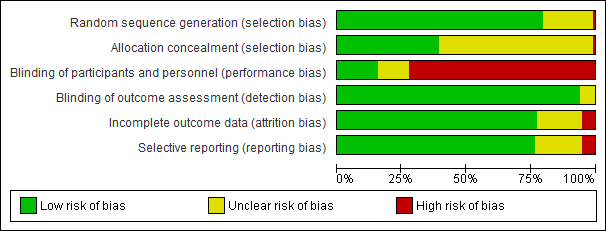
**

**Figure S2.** Risk of bias graph: These are review authors' judgments about each risk of bias item presented as percentages across all included studies

### **Appendix 7. Results from pairwise meta-analysis for each outcome: numbers, estimates and heterogeneity**

**Table S3.** Summary numbers of studies and patients from pair-wise meta-analysis of direct comparisons

|  | **Primary outcome** | **Secondary efficacy outcomes** | | | | | | **Safety outcomes** | |
| --- | --- | --- | --- | --- | --- | --- | --- | --- | --- |
|  | **All-cause mortality at the end of treatment or longest follow-up**  **(N/n)*** | **SARS-CoV-2 clearance rate**  **(N/n)** | **Time to SARS-CoV-2 clearance**  **(N/n)** | **Length in hospital stay**  **(N/n)** | **Improvement of lung disease on CT (N/n)** | **Progression of COVID-19 disease(N/n)** | **N patients discharged (N/n)** | **Any adverse events**  **(N/n)** | **Serious adverse events**  **(N/n)** |
| **Anti-C5a antibody (IFX-1) vs.** |  |  |  |  |  |  |  |  |  |
| SC | 1/30 | NA | NA | NA | NA | NA | NA | NA | 1/30 |
| **Aprepitant+Corticosteroid vs.** |  |  |  |  |  |  |  |  |  |
| Corticosteroid | 1/18 | NA | NA | NA | NA | NA | 1/18 | NA | NA |
| **Auxora vs.** |  |  |  |  |  |  |  |  |  |
| SC | 1/26 | NA | NA | NA | NA | NA | NA | 1/30 | 1/30 |
| **Azithromycin plus Lopinavir/Ritonavir**  **plus Hydroxychloroquine vs.** |  |  |  |  |  |  |  |  |  |
| Lopinavir/Ritonavir  plus Hydroxychloroquine | 1/111 | NA | NA | 1/111 | NA | NA | NA | NA | NA |
| **Azvudina vs.** |  |  |  |  |  |  |  |  |  |
| SC | 1/20 | 1/20 | 1/20 | NA | NA | NA | NA | 1/20 | 1/20 |
| **Baloxavir vs.** |  |  |  |  |  |  |  |  |  |
| SC | 1/20 | 1/20 | NA | NA | NA | NA | NA | NA | NA |
| Favipiravir | 1/19 | 1/19 | NA | NA | NA | NA | NA | NA | NA |
| **Bromhexine Hydrochloride** |  |  |  |  |  |  |  |  |  |
| SC | 1/78 | 1/18 | NA | NA | 1/18 | NA | 1/18 | 1/78 | NA |
| **Calcifediol vs.** |  |  |  |  |  |  |  |  |  |
| SC | 1/76 | NA | NA | NA | NA | NA | 1/76 | NA | NA |
| **Chloroquine vs.** |  |  |  |  |  |  |  |  |  |
| Lopinavir/Ritonavir | NA | 1/22 | NA | NA | 1/22 | NA | 1/22 | NA | NA |
| SC | 1/39 | NA | NA | NA | NA | 1/39 | NA | 1/39 | 1/39 |
| **Colchicine vs.** |  |  |  |  |  |  |  |  |  |
| SC/Placebo | 2/143 | NA | NA | NA | NA | NA | NA | 1/105 | NA |
| **Corticosteroids vs.** |  |  |  |  |  |  |  |  |  |
| SC/Placebo | 7/7652 | NA | NA | NA | NA | NA | 2/6724 | 1/62 | 3/686 |
| **Darunavir/Cobicistat vs.** |  |  |  |  |  |  |  |  |  |
| SC | 1/30 | 1/30 | 1/30 | NA | NA | 1/30 | NA | 1/30 | NA |
| **Favipiravir vs.** |  |  |  |  |  |  |  |  |  |
| SC | 4/327 | 5/494 | 2/318 | 1/150 | 2/228 | 1/168 | 2/207 | 3/371 | 2/311 |
| Tocilizumab | NA | NA | NA | NA | 1/12 | NA | NA | 1/12 | 1/12 |
| Umifenovir | 1/236 | NA | NA | NA | NA | NA | NA | 1/236 | NA |
| **Favipiravir plus**  **Interferon beta 1b vs.** |  |  |  |  |  |  |  |  |  |
| Hydroxychloroquine | 1/89 | NA | NA | NA | NA | NA | 1/89 | NA | NA |
| **Favipiravir plus**  **Tocilizumab vs.** |  |  |  |  |  |  |  |  |  |
| Favipiravir | NA | NA | NA | NA | 1/21 | NA | NA | 1/21 | 1/21 |
| Tocilizumab | NA | NA | NA | NA | 1/19 | NA | NA | 1/19 | 1/19 |
| **Fluvoxamine vs.** |  |  |  |  |  |  |  |  |  |
| SC/Placebo | 1/152 | NA | NA | NA | NA | 1/152 | NA | 1/152 | 1/152 |
| **Hydroxychloroquine vs.** |  |  |  |  |  |  |  |  |  |
| SC/Placebo | 12/8767 | 7/1319 | 2/346 | 3/770 | 1/62 | 7/6910 | 3/5643 | 9/1761 | 8/2148 |
| Azithromycin | 1/85 | NA | NA | NA | NA | NA | NA | 1/83 | NA |
| Chloroquine | 1/53 | NA | NA | NA | NA | 1/53 | NA | 1/53 | 1/53 |
| **Hydroxychloroquine plus Azithromycin vs.** |  |  |  |  |  |  |  |  |  |
| Hydroxychloroquine | 3/1189 | 1/304 | NA | 1/438 | NA | NA | 2/885 | 2/742 | 3/1181 |
| SC | 2/748 | 1/304 | NA | 1/444 | NA | NA | 1/444 | 2/748 | 2/748 |
| **Hydroxychloroquine plus**  **Zinc vs.** |  |  |  |  |  |  |  |  |  |
| Hydroxychloroquine | 1/191 | NA | NA | NA | NA | NA | 1/191 | NA | NA |
| **Human-Granulocyte-Colony–Stimulating Factor(rhG-CSF) vs.** |  |  |  |  |  |  |  |  |  |
| SC | 1/200 | NA | NA | NA | NA | 1/200 | NA | 1/200 | 1/200 |
| **Icatibant vs.** |  |  |  |  |  |  |  |  |  |
| Inhibitor C1 esterase/kallikrein | 1/20 | NA | NA | 1/20 | NA | NA | NA | NA | NA |
| SC | 1/20 | NA | NA | 1/20 | NA | NA | NA | NA | NA |
| **Immunoglobulin vs.** |  |  |  |  |  |  |  |  |  |
| SC/Placebo | 3/177 | NA | NA | NA | NA | NA | NA | 1/34 | NA |
| **Inhibitor of C1 esterase/kallikrein** |  |  |  |  |  |  |  |  |  |
| SC | 1/20 | NA | NA | 1/20 | NA | NA | NA | NA | NA |
| **Interferon alpha 2b plus Interferon gamma vs** |  |  |  |  |  |  |  |  |  |
| Interferon alpha 2b | 1/63 | 1/63 | NA | NA | NA | 1/63 | 1/63 | 1/63 | NA |
| **Interferon beta 1a vs** |  |  |  |  |  |  |  |  |  |
| SC/Placebo | 3/4290 | NA | NA | 1/92 | NA | 1/3831 | 2/190 | 2/190 | 1/98 |
| **Interferon beta 1b vs** |  |  |  |  |  |  |  |  |  |
| SC | 1/80 | NA | NA | NA | NA | NA | 1/80 | NA | NA |
| **Itolizumab vs.** |  |  |  |  |  |  |  |  |  |
| SC | 1/32 | NA | NA | NA | NA | NA | NA | 1/32 | NA |
| **Ivermectin vs.** |  |  |  |  |  |  |  |  |  |
| SC/Placebo | 1/90 | NA | NA | NA | NA | NA | NA | 1/45 | 1/45 |
| **Ivermectin plus Doxycycline vs.** |  |  |  |  |  |  |  |  |  |
| Hydroxychloroquine plus Azithromycin | 1/125 | NA | NA | NA | 1/84 | 1/34 | NA | 1/125 | NA |
| SC | 1/140 | NA | NA | NA | NA | 1/129 | NA | NA | NA |
| **Leflunomide plus Interferon alpha 2a vs.** |  |  |  |  |  |  |  |  |  |
| Interferon alpha 2a | 1/52 | NA | NA | NA | NA | 1/52 | NA | NA | 1/52 |
| **Lincomycin vs** |  |  |  |  |  |  |  |  |  |
| Azithromycin | NA | 1/24 | NA | NA | NA | NA | NA | NA | NA |
| **Lopinavir/Ritonavir vs.** |  |  |  |  |  |  |  |  |  |
| SC | 3/8010 | 2/250 | 1/51 | NA | 1/42 | 3/7432 | 1/5040 | 2/245 | 2/245 |
| Umifenovir | NA | 1/69 | 1/69 | NA | 1/61 | 1/69 | NA | 1/69 | 1/69 |
| **Lopinavir/Ritonavir + Interferon alpha vs.** |  |  |  |  |  |  |  |  |  |
| Ribavirin + Interferon alpha | 1/69 | 1/69 | 1/69 | NA | NA | 1/69 | NA | 1/69 | 1/69 |
| **Lopinavir/Ritonavir plus Ribavirin plus Interferon beta1b vs.** |  |  |  |  |  |  |  |  |  |
| Lopinavir/Ritonavir | 1/127 | NA | 1/127 | 1/127 | NA | NA | NA | 1/127 | 1/127 |
| **LY-CoV555 vs.** |  |  |  |  |  |  |  |  |  |
| SC/Placebo | 1/467 | NA | NA | NA | NA | NA | NA | 1/452 | 1/452 |
| **Nitazoxamide vs.** |  |  |  |  |  |  |  |  |  |
| SC/Placebo | NA | 1/475 | NA | NA | NA | NA | NA | 1/392 | 1/392 |
| **Novaferon vs.** |  |  |  |  |  |  |  |  |  |
| Lopinavir/Ritonavir | NA | 1/59 | NA | NA | NA | 1/56 | NA | 1/59 | 1/59 |
| **Novaferon + Lopinavir/Ritonavir vs.** |  |  |  |  |  |  |  |  |  |
| Lopinavir/Ritonavir | NA | 1/59 | NA | NA | NA | 1/56 | NA | 1/59 | 1/59 |
| Novaferon | NA | 1/60 | NA | NA | NA | 1/56 | NA | 1/60 | 1/60 |
| **c-Plasma vs.** |  |  |  |  |  |  |  |  |  |
| SC/Placebo | 7/1187 | 3/648 | NA | 3/523 | NA | 3/837 | 2/436 | 4/555 | 2/419 |
| fresh frozen Plasma | 1/29 | NA | NA | NA | NA | 1/29 | NA | NA | NA |
| **Pentoxyfilline vs.** |  |  |  |  |  |  |  |  |  |
| SC | 1/54 | NA | NA | 1/54 | NA | 1/54 | NA | 1/54 | NA |
| **Perginterferon lambda 1a vs.** |  |  |  |  |  |  |  |  |  |
| SC/Placebo | NA | NA | 1/120 | NA | NA | NA | NA | 1/120 | 1/120 |
| **Remdesivir vs.** |  |  |  |  |  |  |  |  |  |
| SC/Placebo | 4/7345 | 1/236 | NA | NA | NA | 2/5560 | 3/1894 | 3/1877 | 3/1877 |
| **Ribavirin plus Lopinavir/Ritornavir plus Interferon alpha** |  |  |  |  |  |  |  |  |  |
| Ribavirin+ Interferon alpha | 1/65 | 1/65 | 1/65 | NA | NA | 1/65 | NA | 1/65 | 1/65 |
| Lopinavir/Ritonavir | 1/68 | 1/68 | NA | NA | NA | 1/68 | NA | 1/68 | 1/68 |
| **Ruxolitinib vs.** |  |  |  |  |  |  |  |  |  |
| SC | 1/41 | NA | 1/41 | NA | 1/41 | NA | NA | 1/41 | 1/41 |
| **Sofosbuvir plus Daclastavir**  **plus Ribavirin** |  |  |  |  |  |  |  |  |  |
| SC | 1/48 | NA | NA | NA | NA | NA | NA | NA | NA |
| **Sofosbuvir plus Daclastavir** |  |  |  |  |  |  |  |  |  |
| SC | 2/155 | 1/89 | NA | NA | NA | 1/89 | NA | NA | 2/155 |
| **Telmisartan vs.** |  |  |  |  |  |  |  |  |  |
| SC | 1/82 | NA | NA | 1/82 | NA | NA | 1/82 | NA | NA |
| **Tocilizumab vs.**  SC/Placebo |  |  |  |  |  |  |  |  |  |
|  | 5/1324 | NA | NA | 2/829 | NA | 3/495 | 2/365 | 4/1024 | 6/1390 |
| **Triazavirin vs.** |  |  |  |  |  |  |  |  |  |
| SC/Placebo | 1/52 | 1/52 | NA | NA | 1/43 | NA | NA | 1/52 | 1/52 |
| **Umbilical cord mesenchymal stem cells (hUC-MSC) vs.** |  |  |  |  |  |  |  |  |  |
| SC/Placebo | 2/141 | NA | NA | NA | NA | 1/41 | 1/41 | 1/100 | NA |
| **Umifenovir vs.** |  |  |  |  |  |  |  |  |  |
| SC | NA | 1/52 | 1/52 | NA | 1/47 | 1/52 | NA | 1/52 | 1/52 |
| **Umifenovir plus Hydroxychloroquine vs.** |  |  |  |  |  |  |  |  |  |
| Lopinavir/Ritonavir plus Hydroxychloroquine | 1/100 | NA | NA | 1/100 | NA | NA | NA | 1/100 | NA |

* N= number of studies; n= number of patients; NA= not available; SC= standard care.

**Table S4.** Summary estimates from pairwise meta-analysis of direct comparisons

|  | **Primary outcome** | **Secondary efficacy outcomes** | | | | | | **Safety outcomes** | | |  |
| --- | --- | --- | --- | --- | --- | --- | --- | --- | --- | --- | --- |
|  | **All-cause mortality at the end of treatment or longest follow-up**  **RR (95% CI)*** | **SARS-CoV-2 clearance rate**  **RR (95% CI)** | **Time to SARS-CoV-2 clearance**  **HR/SMD (95%CI)** | **Length in hospital stay**  **HR/SMD (95%CI)** | **Improvement of lung disease on CT**  **RR (95% CI)** | **Progression of COVID-19 disease**  **RR (95% CI)** | **N patients discharged**  **RR (95% CI)** | | **Any adverse event**  **RR (95% CI)** | **Serious adverse event**  **RR (95% CI)** | |
| **Anti-C5a antibody (IFX-1) vs.** |  |  |  |  |  |  |  | |  |  | |
| SC | 0.50 (0.11,2.33) | NA | NA | NA | NA | NA | NA | | NA | 1.29 (0.65,2.54) | |
| **Aprepitant+Corticosteroid vs.** |  |  |  |  |  |  |  | |  |  | |
| Corticosteroid | 1.25 (0.09,17.02) | NA | NA | NA | NA | NA | 1.25 (0.09,17.02) | | NA | NA | |
| **Auxora vs.** |  |  |  |  |  |  |  | |  |  | |
| SC | 0.53 (0.09,3.16) | NA | NA | NA | NA | NA | NA | | 0.94 (0.63,1.40) | 0.60 (0.24,1.49) | |
| **Azithromycin plus Lopinavir/Ritonavir**  **plus Hydroxychloroquine vs.** |  |  |  |  |  |  |  | |  |  | |
| Lopinavir/Ritonavir  plus Hydroxychloroquine | 0.33 (0.01,7.87) | NA | NA | **-046 (-0.84,-0.08)^b^** | NA | NA | NA | | NA | NA | |
| **Azvudine vs.** |  |  |  |  |  |  |  | |  |  | |
| SC | 0 | **2.33 (1.13,4.80)** | **-1.27 (-2.25, -0.29)^b^** | NA | NA | NA | NA | | 0.14 (0.01,2.45) | 0 | |
| **Baloxavir vs.** |  |  |  |  |  |  |  | |  |  | |
| SC | 0 | 0.71 (0.47,1.09) | NA | NA | NA | NA | NA | | NA | NA | |
| Favipiravir | 0 | 0.90 (0.53,1.54) | NA | NA | NA | NA | NA | | NA | NA | |
| **Bromhexine Hydrochloride** |  |  |  |  |  |  |  | |  |  | |
| SC | 0.09 (0.01,1.59) | 0.95 (0.72,1.27) | NA | NA | 2.00 (0.60,6.64) | NA | 2.50 (0.78,7.97) | | 0 | NA | |
| **Calcifediol vs.** |  |  |  |  |  |  |  | |  |  | |
| SC | 0.11 (0.01,2.13) | NA | NA | NA | NA | NA | 1.09 (0.96,1.23) | | NA | NA | |
| **Chloroquine vs.** |  |  |  |  |  |  |  | |  |  | |
| Lopinavir/Ritonavir | NA | 1.20 (0.82,1.77) | NA | NA | 2.40 (0.25,22.75) | NA | **1.91 (1.09,3.34)** | | NA | NA | |
| SC | 0 | NA | NA | NA | NA | 0 | NA | | 2.24 (0.55,9.13) | 0 | |
| **Colchicine vs.** |  |  |  |  |  |  |  | |  |  | |
| SC/Placebo | 0.23 (0.03,1.97) | NA | NA | NA | NA | NA | NA | | **2.17 (1.29,3.65)** | NA | |
| **Corticosteroids vs.** |  |  |  |  |  |  |  | |  |  | |
| SC/Placebo | 0.88 (0.76,1.02) | NA | NA | NA | NA | NA | 1.25 (0.82,1.91) | | 0.82 (0.12,5.48) | 1.47 (0.31,7.04) | |
| **Darunavir/Cobicistat vs.** |  |  |  |  |  |  |  | |  |  | |
| SC | 0 | 0.78 (0.39,1.54) | 0.82 (0.36,1.87)^a^ | NA | NA | 3.00 (0.13,68.26) | NA | | 1.14 (0.56,2.35) | NA | |
| **Favipiravir vs.** |  |  |  |  |  |  |  | |  |  | |
| SC | 0.56 (0.09,3.51) | 1.01 (0.93,1.09) | 1.32 (1.03,1.69)^a^ | 1.41 (0.97,2.03)^a^ | 1.14 (0.90,1.44) | 1.50 (0.16,14.09) | 1.02 (0.91,1.15) | | 1.92 (0.83,4.43) | 0.99 (0.11,8.85) | |
| Tolicizumab | NA | NA | NA | NA | 3.16^a^(0.62,16.10) | NA | NA | | 0.71 (0.15,3.50) | 0 | |
| Umifenovir | 0 | NA | NA | NA | NA | NA | NA | | 1.37 (0.90,2.08) | NA | |
| **Favipiravir plus Interferon beta 1b vs.** |  |  |  |  |  |  |  | |  |  | |
| Hydroxychloroquine | 0.85 (0.28,2.59) | NA | NA | NA | NA | NA | 0.96 (0.72,1.28) | | NA | NA | |
| **Favipiravir plus Tocilizumab vs.** |  |  |  |  |  |  |  | |  |  | |
| Favipiravir | NA | NA | NA | NA | 2.66^a^ (1.08,6.53) | NA | NA | | 2.25 (0.65,7.73) | 0 | |
| Tocilizumab | NA | NA | NA | NA | 1.28^a^ (0.39,4.23) | NA | NA | | 1.61 (0.51,5.04) | 0 | |
| **Fluvoxamine vs.** |  |  |  |  |  |  |  | |  |  | |
| SC/Placebo | 0 | NA | NA | NA | NA | 0.30 (0.01,7.26) | NA | | 1.65 (0.64,4.23) | 0.18 (0.02,1.50) | |
| **Hydroxychloroquine vs.** |  |  |  |  |  |  |  | |  |  | |
| SC/Placebo | 1.07 (0.98,1.18) | 0.99 (0.91,1.07) | 0.85 (0.58,1.23)^a^  -0.23  (-051,0.05)^b^ | 0.06  (-0.15,0.27)^b^ | **3.80 (1.62,8.89)** | 1.07 (0.90,1.27) | 0.97 (0.94,1.01) | | **1.99 (1.13,3.51)** | 1.01 (0.65,1.56) | |
| Azithromicin | 6.14 (0.77,48.87) | NA | NA | NA | NA | NA | NA | | 0.95 (0.88,1.03) | NA | |
| Chloroquine | 0 | NA | NA | NA | NA | 0 | NA | | 1.00 (0.46,2.20) | 0 | |
| **Hydroxychloroquine plus Azithromycin vs.** |  |  |  |  |  |  |  | |  |  | |
| Hydroxychloroquine | 0.87 (0.44,1.71) | 0.71 (0.47,1.08) | NA | 0.07  (-0.12,0.26)^b^ | NA | NA | 1.04 (0.68,1.59) | | 1.00 (0.56,1.77) | 1.12 (0.89,1.41) | |
| SC/Placebo | 0.52 (0.13,2.07) | 0.67 (0.45,1.00) | NA | 0.08  (-0.11,0.27)^b^ | NA | NA | 0.99 (0.91,1.07) | | **1.39 (1.06,1.82)** | 1.05 (0.21,5.13) | |
| **Hydroxychloroquine plus**  **Zinc vs.** |  |  |  |  |  |  |  | |  |  | |
| Hydroxychloroquine | 0.99 (0.30,3.31) | NA | NA | -0.09^b^(-0.37,122) | NA | NA | NA | | NA | NA | |
| **Human-Granulocyte-Colony–Stimulating Factor(rhG-CSF) vs.** |  |  |  |  |  |  |  | |  |  | |
| SC | **0.25 (0.07,0.86)** | NA | NA | NA | NA | **0.13 (0.03,0.57)** | NA | | **2.02 (1.62,2.50)** | 0.72 (0.49,1.05) | |
| **Icatibant vs.** |  |  |  |  |  |  |  | |  |  | |
| Inhibitor C1 esterase/kallikrein | 0.33 (0.02,7.32) | NA | NA | -0.30  (-1.19,0.58)^b^ | NA | NA | NA | | NA | NA | |
| SC | 0.33 (0.02,7.32) | NA | NA | 0.06  (-0.82,0.94)^b^ | NA | NA | NA | | NA | NA | |
| **Immunoglobulin vs.** |  |  |  |  |  |  |  | |  |  | |
| SC/placebo | 0.65 (0.29,1.44) | NA | NA | NA | 2.15 (0.48,9.74) | NA | NA | | 0 | NA | |
| **Inhibitor of C1 esterase/kallikrein** |  |  |  |  |  |  |  | |  |  | |
| SC | 1.00 (0.07,13.87) | NA | NA | 0.39  (-0.50,1.27)^b^ | NA | NA | NA | | NA | NA | |
| **Interferon alpha 2b plus Interferon gamma vs** |  |  |  |  |  |  |  | |  |  | |
| Interferon alpha 2b | 0 | **1.49 (1.01,2.19)** | NA | NA | NA | 0 | 1.10 (0.97,1.24) | | 1.38 (0.63,3.02) | NA | |
| **Interferon beta 1a vs** |  |  |  |  |  |  |  | |  |  | |
| SC/Placebo | 0.69 (0.30, 1.61) | NA | NA | 1.37^a^(0.85,2.21)  0.32 ^b^  (-0.09,0.73) | NA | 1.00 (0.83,1.43) | 1.19 (0.98,1.92) | | 3.14 (0.13,78.69) | NA | |
| **Interferon beta 1b vs** |  |  |  |  |  |  |  | |  |  | |
| SC | 0.33 (0.04,3.07) | NA | NA | NA | NA | NA | 1.44 (0.96,2.18) | | NA | NA | |
| **Itolizumab vs.** |  |  |  |  |  |  |  | |  |  | |
| SC | 0.07 (0.00,1.21) | NA | NA | NA | NA | NA | NA | | 2.05 (0.93,4.48) | NA | |
| **Ivermectin vs.** |  |  |  |  |  |  |  | |  |  | |
| SC/Placebo | 0.18 (0.02,1.34) | NA | NA | NA | NA | NA | NA | | 1.30 (0.57,2.96) | 1.55 (0.07,35.89) | |
| **Ivermectin plus Doxycycline vs.** |  |  |  |  |  |  |  | |  |  | |
| Hydroxychloroquine plus Azithromycin | 1.09 (0.98,1.22) | NA | NA | NA | NA | NA | NA | | 0.72 (0.45,1.16) | NA | |
| SC | 0.33 (0.07,1.60) | NA | NA | NA | NA | 0.43 (0.12,1.59) | NA | | NA | NA | |
| **Leflunomide plus Interferon alpha 2a vs.** |  |  |  |  |  |  |  | |  |  | |
| Interferon alpha 2a | 0 | NA | NA | NA | NA | 0 | NA | | NA | 2.50 (0.90,6.96) | |
| **Lincomycin vs** |  |  |  |  |  |  |  | |  |  | |
| Azithromycin | NA | **2.50 (1.08,5.79)** | NA | NA | NA | NA | NA | | NA | NA | |
| **Lopinavir/Ritonavir vs.** |  |  |  |  |  |  |  | |  |  | |
| SC | 1.02 (0.92,1.12) | 1.00 (0.75,1.32) | -0.06  (-0.64,0.52)^b^ | NA | NA | 1.10 (0.94,1.30) | NA | | 2.59 (0.17,38.90) | 0.63 (0.39,1.03) | |
| Umifenovir | NA | 0.93 (0.79,1.11) | -0.20  (-0.49,0.45)^b^ | NA | 1.08 (0.79,1.47) | 2.75 (0.79,9.49) | NA | | 2.47 (0.97,6.26) | 3.09 (0.13,73.21) | |
| **Lopinavir/Ritonavir + Interferon alpha vs.** |  |  |  |  |  |  |  | |  |  | |
| Ribavirin + Interferon alpha | 0 | 1.19 (0.78,1.81) | 1.50 (0.89,2.53)^a^ | NA | NA | 1.83 (0.17,19.29) | NA | | 1.04 (0.77,1.40) | 0 | |
| **Lopinavir/Ritonavir + Ribavirin + Interferon beta1b vs.** |  |  |  |  |  |  |  | |  |  | |
| Lopinavir/Ritonavir | 0 | NA | **4.37 (1.86,10.27)^a^** | **2.72 (1.20,6.16)^a^** | NA | NA | NA | | 0.98 (0.67,1.43) | 0.16 (0.01,3.87) | |
| **LY-CoV555 vs.** |  |  |  |  |  |  |  | |  |  | |
| SC/Placebo | 0 | NA | NA | NA | NA | NA | NA | | 0.91 (0.64,1.30) | 0.15 (0.01,3.78) | |
| **Nitazoxamide vs.** |  |  |  |  |  |  |  | |  |  | |
| SC/Placebo | NA | **1.60 (1.10,2.33)** | NA | NA | NA | NA | NA | | 1.02 (0.76,1.38) | 1.00 (0.06,15.83) | |
| **Novaferon vs.** |  |  |  |  |  |  |  | |  |  | |
| Lopinavir/Ritonavir | NA | 1.10 (0.68,1.75) | NA | NA | NA | 0.11 (0.01,1.97) | NA | | 0.93 (0.76,1.14) | 0 | |
| **Novaferon + Lopinavir/Ritonavir vs.** |  |  |  |  |  |  |  | |  |  | |
| Lopinavir/Ritonavir | NA | 1.35 (0.89,2.06) | NA | NA | NA | 0.11 (0.01,1.97) | NA | | 0.93 (0.76,1.14) | 0 | |
| Novaferon | NA | 1.24 (0.84,1.83) | NA | NA | NA | 0 | NA | | 1.030 (0.80,1.25) | 0 | |
| **c-Plasma vs.** |  |  |  |  |  |  |  | |  |  | |
| SC/Placebo | 0.94 (0.70,1.24) | **1.82 (1.19,2.77)** | NA | 1.08 ^b^ (0.80,1.48) | NA | 0.88 (0.57,1.37) | 1.1 (0.78,1.58**)** | | 1.08 (0.91,1.28) | 0 | |
| fresh frozen Plasma |  | NA | NA | NA | NA |  | NA | | NA | NA | |
| **Pentoxyfilline vs** |  |  |  |  |  |  |  | |  |  | |
| SC | 0.38 (0.09,1.50) | NA | NA | -0.34^b^  (-0.91,0.23) | NA | 0.50 (0.11,2.23) | NA | | 1.54 (0.07,36.04) | NA | |
| **Perginterferon lambda 1a vs.** |  |  |  |  |  |  |  | |  |  | |
| SC/Placebo | NA | NA | 0.81^a^(0.56,1.17) | NA | NA | NA | NA | | 1.19 (0.75,1.88) | 1.00 (0.15,6.87) | |
| **Remdesivir vs.** |  |  |  |  |  |  |  | |  |  | |
| SC/Placebo | 0.93 (0.81,1.07) | 0.91 (0.75,1.10) | NA | NA | NA | 0.48 (0.07,3.51) | **1.13 (1.02,1.24)** | | 1.01 (0.88,1.16) | **0.75 (0.63,0.89)** | |
| **Ribavirin plus Lopinavir/Ritornavir plus Interferon alpha** |  |  |  |  |  |  |  | |  |  | |
| Ribavirin+ Interferon alpha | 0 | 0.91 (0.55,1.49) | 1.39 (0.80,2.42)^a^ | NA | NA | 2.06 (0.20,21.64) | NA | | **1.35 (1.06,1.71)** | 0 | |
| Lopinavir/Ritonavir | 0 | 0.77 (0.49,1.20) | NA | NA | NA | 1.13 (0.17,7.53) | NA | | **1.30 (1.04,1.62)** | 0 | |
| **Ruxolitinib vs.** |  |  |  |  |  |  |  | |  |  | |
| SC | 0.15 (0.01,2.73) | NA | 1.28(0.44,3.69) ^a^ | NA | **1.45 (1.01,2.10)** | NA | NA | | 1.12 (0.79,1.59) | 0.12 (0.01,2.03) | |
| **Sofosbuvi plus Daclastavir**  **plus Ribavirin** |  |  |  |  |  |  |  | |  |  | |
| SC | 0.14 (0.01,2.62) | NA | NA | NA | NA | NA | NA | | NA | NA | |
| **Sofosbuvir plus Daclastavir** |  |  |  |  |  |  |  | |  |  | |
| SC | 0.51 (0.16,2.31) | 1.06 (0.76,1.47) | NA | NA | NA | 0.41 (0.08,2.00) | NA | | NA | 0 | |
| **Telmisartan vs.** |  |  |  |  |  |  |  | |  |  | |
| SC | 1.00 (0.15,6.76) | NA | NA | **2.02 (1.14,3.58)^a^** | NA | NA | **1.61 (1.08,2.40)** | | NA | NA | |
| **Tocilizumab vs.**  SC/Placebo |  |  |  |  |  |  |  | |  |  | |
|  | 1.10 (0.80,1.51) | NA | NA | **1.24 (1.03,1.49)^a^** | NA | 0.68 (0.43,1.09) | 1.01 (0.94,1.09) | | 1.03 (0.80,1.33) | 0.88 (0.73,1.07) | |
| **Triazavirin vs.** |  |  |  |  |  |  |  | |  |  | |
| SC/Placebo | 0.33 (0.01,7.82) | 1.14 (0.92,1.42) | NA | NA | 1.92 (0.85,4.33) | NA | NA | | 0.60 (0.26,1.41) | 0.80 (0.24,2.65) | |
| **Umbilical cord mesenchymal stem cells (hUC-MSC) vs.** |  |  |  |  |  |  |  | |  |  | |
| SC/Placebo | 0.33 (0.02,5.94) | NA | NA | NA | NA | 0.26 (0.01,4.43) | 2.42 (0.85,6.85) | | 0.92 (0.65,1.31) | NA | |
| **Umifenovir vs.** |  |  |  |  |  |  |  | |  |  | |
| SC | NA | 1.20 (0.90,1.59) | -0.04 ^b^  (-0.62,0.54) | NA | **0.75 (0.57,0.98)** | 1.46 (0.16,12.99) | NA | | 5.50 (0.32,94.06) | 0 | |
| **Umifenovir plus Hydroxychloroquine vs.** |  |  |  |  |  |  |  | |  |  | |
| Lopinavir/Ritonavir plus Hydroxychloroquine | 0.50 (0.05,5.34) | NA | NA | **-0.48 (-0.88,-0.08)^b^** | NA | NA | NA | | **0.25 (0.08,0.83)** | NA | |

Significant results are bolded and underscored. Regarding dichotomous outcomes: for all-cause mortality, adverse events and serious adverse events, an RR above 1 favours the second treatment, for all others outcome an RR above 1 favours the first treatment. Regarding continuous outcomes: a SMD above 0 favours the first treatment and a HR above 1 favours first treatment.

CI= confidence interval, HR= hazard ratio, NA= not available, RR= risk ratio, SC= standard care, SMD= standardized mean difference.

a: HR, b: SMD

**Table S5.** Heterogeneity test result, I2 and heterogeneity estimate

1. **All-cause mortality at the end of treatment or at the longest follow-up**

| **Comparison** | **No. of studies** | **P-value** | **I^2^** | **τ^2^** |
| --- | --- | --- | --- | --- |
| Corticosteroids vs SC | 7 | 0.12 | 41.0% | 0.01 |
| Favipiravir vs SC | 4 | 0.75 | 0.0% | 0.00 |
| Hydroxychloroquine vs SC/Placebo | 12 | 0.86 | 0.0% | 0.00 |
| Hydroxychloroquine+Azythromicin vs Hydroxychloroquine | 3 | 0.22 | 35.0% | 0.13 |
| Immunoglobulin vs SC/Placebo | 3 | 0.10 | 56.0% | 0.26 |
| Interferon beta 1a vs SC/Placebo | 3 | 0.03 | 71.0% | 0.34 |
| Lopinavir/Ritonavir vs SC | 3 | 0.55 | 0.0% | 0.00 |
| c-Plasma vs SC/Placebo | 7 | 0.42 | 0.0% | 0.00 |
| Remdesivir vs SC/Placebo | 4 | 0.38 | 2.0% | 0.00 |
| Sofosbuvir/Daclastavir vs SC | 2 | 0.72 | 0.0% | 0.00 |
| Tocilizumab vs SC/Placebo | 5 | 0.94 | 0.0% | 0.00 |

1. **SARS-CoV-2 clearance rate**

| **Comparison** | **No. of studies** | **P-value** | **I^2^** | **τ^2^** |
| --- | --- | --- | --- | --- |
| Favipiravir vs SC | 5 | 0.23 | 29.0% | 0.00 |
| Hydroxychloroquine vs SC/Placebo | 6 | 0.91 | 0.0% | 0.00 |
| Lopinavir/Ritonavir vs SC | 2 | 0.22 | 34.0% | 0.01 |
| c-Plasma vs SC/Placebo | 3 | 0.02 | 70.0% | 0.12 |

1. **Length of stay in hospital**

| **Comparison** | **No. of studies** | **P-value** | **I^2^** | **τ^2^** |
| --- | --- | --- | --- | --- |
| c-Plasma vs SC | 3 | 0.25 | 29.0% | 0.02 |
| Tocilizumab vs SC/Placebo | 2 | 0.42 | 0.0% | 0.00 |

1. **Progression of COVID-19 disease**

| **Comparison** | **No. of studies** | **P-value** | **I^2^** | **τ^2^** |
| --- | --- | --- | --- | --- |
| Hydroxychloroquine vs SC/Placebo | 7 | 0.67 | 0.0% | 0.00 |
| Lopinavir/Ritonavir vs SC | 3 | 0.33 | 10.0% | 0.00 |
| c-Plasma vs SC/Placebo | 3 | 0.84 | 0.0% | 0.00 |
| Remdesivir vs SC/Placebo | 2 | 0.06 | 72% | 1.59 |
| Tocilizumab vs SC/Placebo | 3 | 0.47 | 0.0% | 0.00 |

1. **N patients discharged at longest follow-up**

| **Comparison** | **No. of studies** | **P-value** | **I^2^** | **τ^2^** |
| --- | --- | --- | --- | --- |
| Corticosteroids vs SC/Placebo | 2 | 0.05 | 75.0% | 0.07 |
| Favipiravir vs SC | 2 | 0.14 | 53.0% | 0.00 |
| Hydroxychloroquine vs SC/Placebo | 3 | 0.61 | 0.0% | 0.00 |
| Hydroxychloroquine+Azythromicin vs Hydroxychloroquine | 2 | 0.04 | 77.0% | 0.07 |
| Interferon beta 1a va SC/Placebo | 2 | 0.38 | 0.0% | 0.00 |
| c-Plasma vs SC/Placebo | 2 | 0.11 | 60.0% | 0.04 |
| Remdesivir vs SC/Placebo | 3 | 0.43 | 0% | 0.00 |
| Tocilizumab vs SC/Placebo | 2 | 0.40 | 0.0% | 0.00 |

1. **Any adverse events**

| **Comparison** | **No. of studies** | **P-value** | **I^2^** | **τ^2^** |
| --- | --- | --- | --- | --- |
| Favipiravir vs SC | 3 | 0.006 | 81.0% | 0.43 |
| Hydroxychloroquine vs SC/Placebo | 7 | <0.00001 | 90.0% | 0.53 |
| Hydroxychloroquine+Azythromicin vs Hydroxychloroquine | 2 | 0.25 | 25.0% | 0.08 |
| Hydroxychloroquine+Azythromicin vs SC/Placebo | 2 | 0.68 | 0.0% | 0.00 |
| Interferon beta 1a va SC/Placebo | 2 | 0.001 | 90.0% | 4.92 |
| Lopinavir/Ritonavir vs SC | 2 | 0.04 | 75.0% | 3.05 |
| c-Plasma vs SC/Plasma | 4 | 0.43 | 0.0% | 0.00 |
| Remdesivir vs SC/Placebo | 3 | 0.08 | 61.0% | 0.01 |
| Tocilizumab vs SC/Placebo | 4 | 0.01 | 73.0% | 0.04 |

1. **Serious adverse events**

| **Comparison** | **No. of studies** | **P-value** | **I^2^** | **τ^2^** |
| --- | --- | --- | --- | --- |
| Corticosteroids vs SC/Placebo | 3 | 0.14 | 49.0% | 0.96 |
| Favipiravir vs SC | 2 | 0.37 | 0.0% | 0.00 |
| Hydroxychloroquine vs SC/Placebo | 5 | 0.81 | 0.0% | 0.00 |
| Hydroxychloroquine+Azythromicin vs Hydroxychloroquine | 3 | 0.73 | 0.0% | 0.00 |
| Lopinavir/Ritonavir vs SC | 2 | 0.50 | 0.0% | 0.00 |
| Remdesivir vs SC/Placebo | 3 | 0.50 | 0.0% | 0.00 |
| Tocilizumab vs SC/Placebo | 6 | 0.75 | 0.0% | 0.00 |

### **Appendix 8. Network of eligible comparisons for all the studies**

Figure S3.

### **Appendix 9. Assessment of inconsistency results for each outcome: global, local and from the node-splitting model.**

**Table S6. Evaluation of the global inconsistency**

| **Model assumption** | **SD Heterogeneity** | **Chi–squared inconsistency** | **P-value** | |
| --- | --- | --- | --- | --- |
| *All-cause mortality* | | | |  |
| Consistency | 1.807 e-10 | Chi–squared (2) = 1.41 | 0.4949 | |
| Inconsistency | 3.213 e-08 |  |  |  |
| *Adverse events* | | | |  |
| Consistency | 0.1247 | Chi–squared (4) = 4.71 | 0.3181 | |
| Inconsistency | 0.2965 |  |  |  |
| *Serious adverse events* | | | |  |
| Consistency | 8.501 e-08 | Chi–squared (3) = 0.12 | 0.9899 | |
| Inconsistency | 6.894 e-09 |  |  |  |

**Table S7. Evaluation of the local inconsistency**

Tests of local inconsistency revealed that the percentages for inconsistent loops were to be expected according to empirical data with the methods of Veroniki et al (Int J Epidemiol 2013; 42:332-45).

1. **Local inconsistency all-cause mortality**

| **Loop** | | | | **IF** | | **seIF** | | **z-value** | | **p-value** | | **95% CI** | | **Loop Heterog tau2** | |
| --- | --- | --- | --- | --- | --- | --- | --- | --- | --- | --- | --- | --- | --- | --- | --- |
|  |  |  |  | |  | |  | |  | |  | |  | |  |
| SC-Hydroxychloroquine Hydroxychloroquine+Azithromycin | | | | 0.634 | | 0.678 | | 0.935 | | 0.35 | | (0.00,1.96) | | 0 | |

1. **Local inconsistency any adverse event**

| **Loop** | | | **IF** | **seIF** | **z-value** | **p-value** | **95% CI** | **Loop Heterog tau2** |
| --- | --- | --- | --- | --- | --- | --- | --- | --- |
|  |  |  |  |  |  |  |  |  |
| SC-Lopinavir/Ritonavir-Umifenovir | | | 2.576 | 1.524 | 1.690 | 0.091 | (0.00,5.56) | 0 |
| SC- Favipiravir-Umifenovir | | | 1.435 | 1.738 | 0.826 | 0.409 | (0.00,4.84) | 0.338 |
| SC- Favipiravir-Tocilizumab | | | 0.786 | 1.434 | 0.548 | 0.584 | (0.00,3.60) | 0.067 |
| SC- Hydroxychloroquine-Hydroxychloroquine+Azithromycin | | | 0.013 | 0.451 | 0.029 | 0.977 | (0.00,0.90) | 0.067 |

1. **Local inconsistency serious adverse events**

| **Loop** | | | **IF** | **seIF** | **z-value** | **p-value** | **95% CI** | **Loop Heterog tau2** |
| --- | --- | --- | --- | --- | --- | --- | --- | --- |
|  |  |  |  |  |  |  |  |  |
| SC-Favipiravir-Tocilizumab | | | 0.498 | 1.988 | 0.251 | 0.802 | (0.00,4.40) | 0 |
| SC- Hydroxychloroquine-Hydroxychloroquine+Azithromycin | | | 0.182 | 0.807 | 0.226 | 0.822 | (0.00,1.76) | 0 |

### **Appendix 10. Comparison-adjusted funnel plot for each outcome from the network meta-analysis.**

**Figure S4.** Comparison-adjusted funnel plot for all-cause mortality at the end of treatment or at longest follow-up **(A),** any adverse event **(B),** serious adverse events **(C).**

**A.**


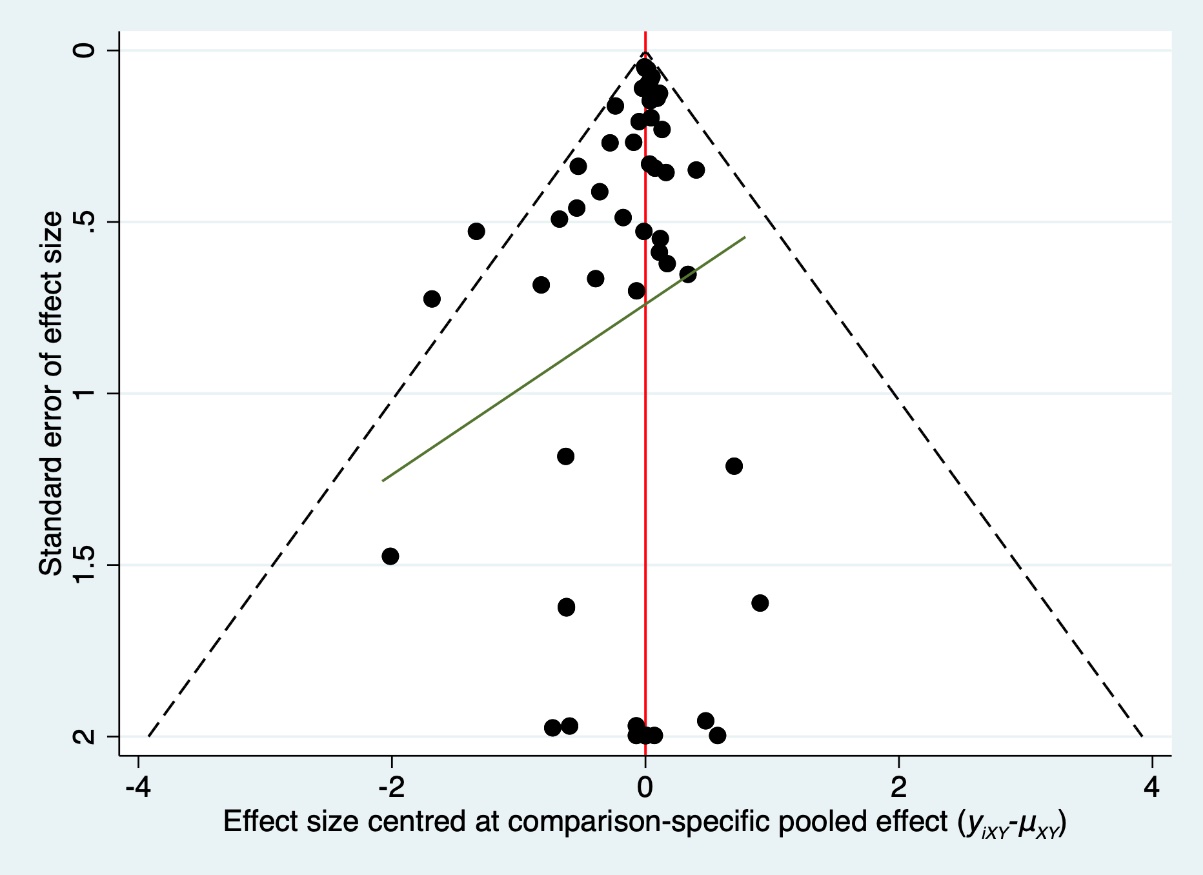


**B.**

**
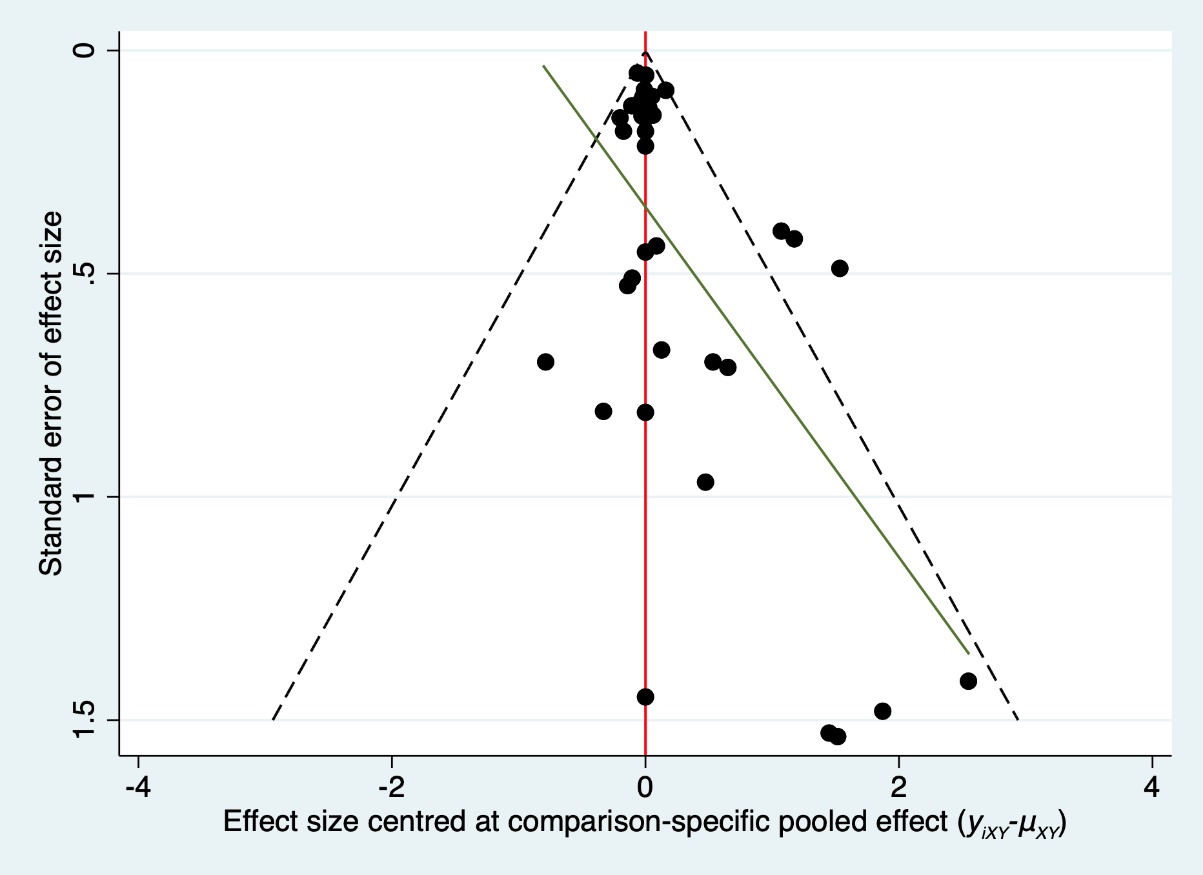
**

**C**


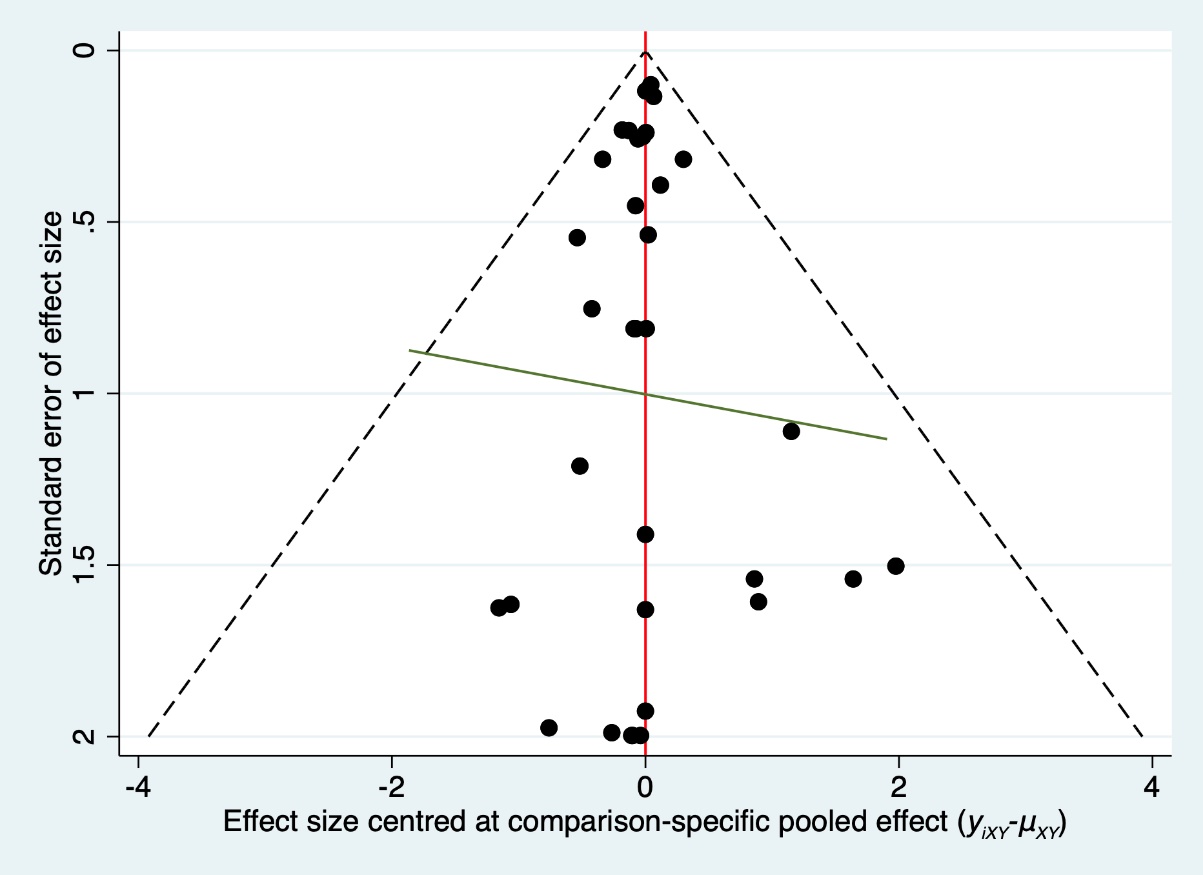


### **Appendix 11. Treatment ranking and SUCRA plot for each outcome.**

**Table S8.** SUCRA treatment ranking for All cause Mortality

| **Treatment** | **SUCRA** | **PrBest** | **MeanRank** |
| --- | --- | --- | --- |
| Corticosteroids | 73.9 | 2.4 | 3.9 |
| Favipiravir | 66.7 | 17 | 4.7 |
| Remdesivir | 63.9 | 1.3 | 5 |
| LY-CoV555 | 62.9 | 40.5 | 5.1 |
| Plasma | 58.7 | 2.9 | 5.5 |
| Umifenovir | 58 | 34.5 | 5.6 |
| SC | 48 | 0 | 6.7 |
| Lopinavir/Ritonavir | 43.2 | 0 | 7.3 |
| Hydroxychloroquine+Azithromycin | 36.5 | 0.5 | 8 |
| Tocilizumab | 32.3 | 0.7 | 8.4 |
| IFN_β-1a | 28.2 | 0.1 | 8.9 |
| Hydroxychloroquine | 27.7 | 0 | 9 |

**Figure S5.** SUCRA plot for All-cause Mortality

Cumulative probability plots (Random Effects model):


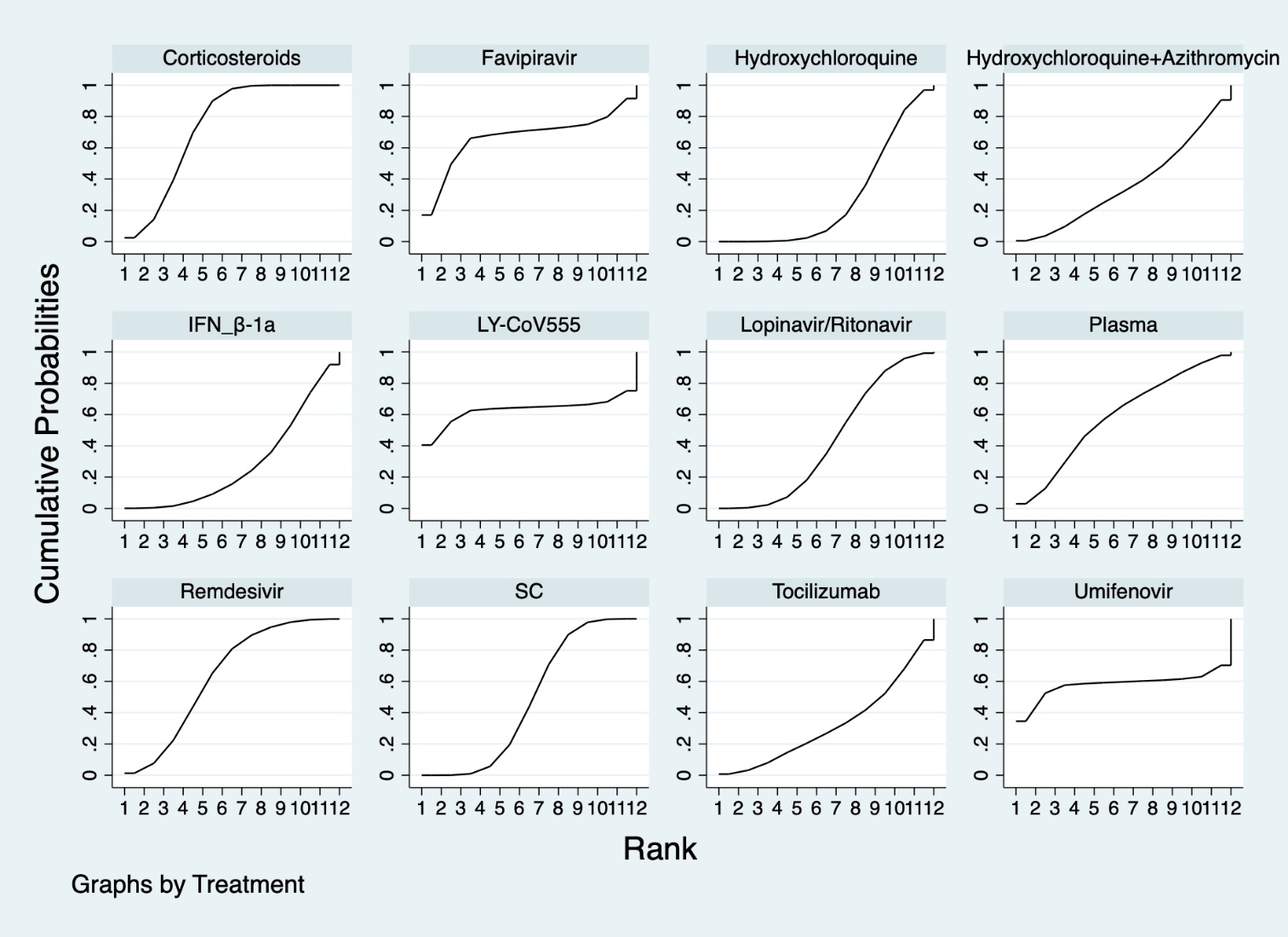


**Table S9.** SUCRA treatment ranking for any adverse event.

| **Treatment** | **SUCRA** | **PrBest** | **MeanRank** |
| --- | --- | --- | --- |
| Corticosteroids | 92.4 | 77.5 | 1.8 |
| Umifenovir | 71.3 | 10.3 | 4.2 |
| LY-CoV555 | 67.6 | 7.1 | 4.6 |
| SC | 59.9 | 0.1 | 5.4 |
| Tocilizumab | 58.3 | 0.7 | 5.6 |
| Remdesivir | 56.6 | 0.3 | 5.8 |
| Nitazoxamide | 53.8 | 2.5 | 6.1 |
| Plasma | 43.7 | 0.4 | 7.2 |
| Lopinavir/Ritonavir | 42.6 | 0.9 | 7.3 |
| Hydroxychloroquine | 25.6 | 0 | 9.2 |
| Hydroxychloroquine+Azithromycin | 15.3 | 0 | 10.3 |
| Favipiravir | 13.1 | 0 | 10.6 |

**Figure S6.** SUCRA plot for any adverse event

Cumulative probability plots (Random Effects model):


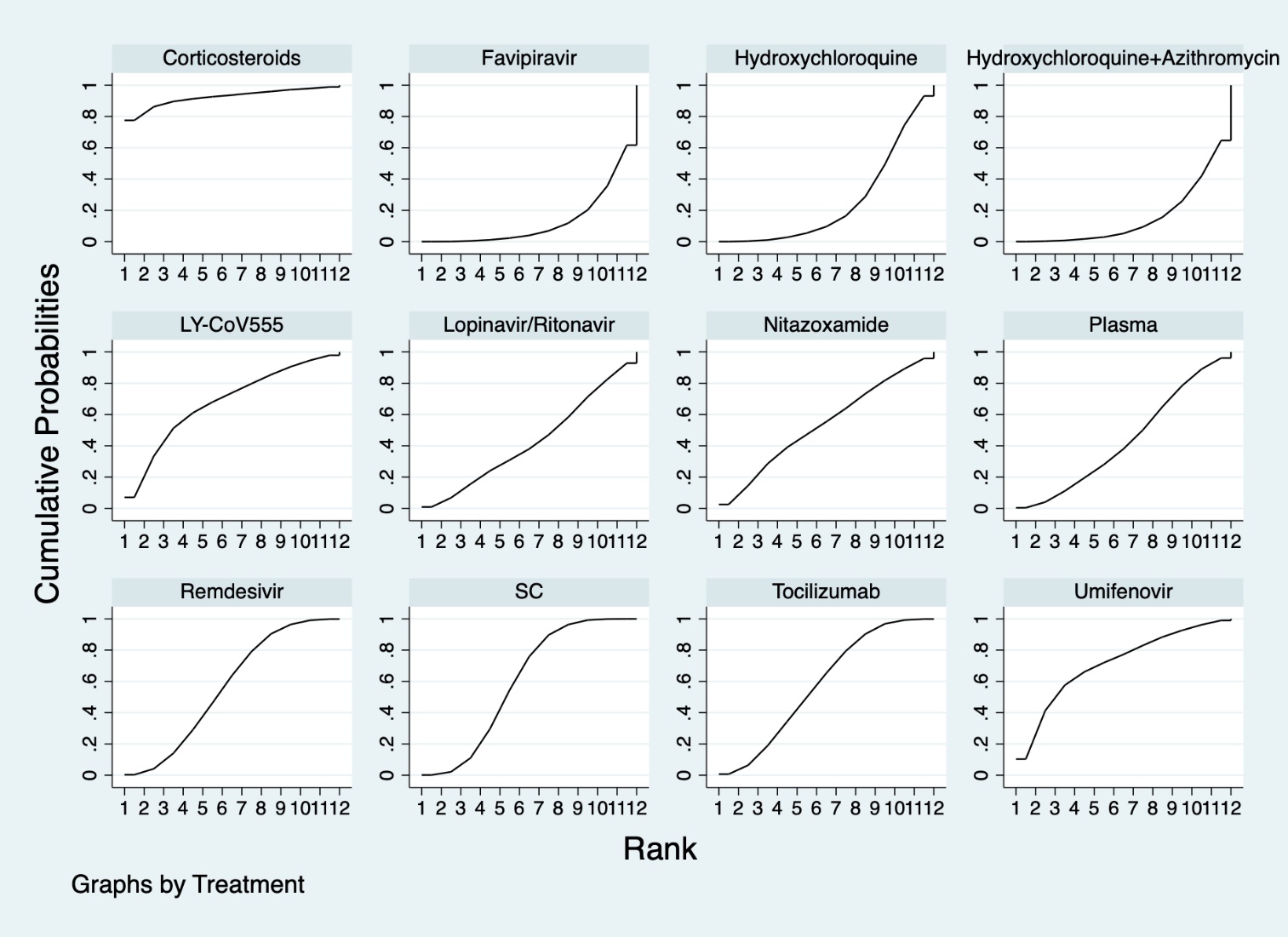


**Table S10.** SUCRA treatment ranking for serious adverse events

| **Treatment** | **SUCRA** | **PrBest** | **MeanRank** |
| --- | --- | --- | --- |
| LY-CoV555 | 85.2 | 70.6 | 2.5 |
| Lopinavir/Ritonavir | 79.5 | 8.1 | 3 |
| Remdesivir | 72.4 | 1.4 | 3.8 |
| Tocilizumab | 56.2 | 0.2 | 5.4 |
| Corticosteroids | 48.9 | 3 | 6.1 |
| Nitazoxamide | 44.7 | 14.5 | 6.5 |
| SC | 40.6 | 0 | 6.9 |
| Favipiravir | 40.6 | 1.9 | 6.9 |
| Hydroxychloroquine | 36.3 | 0.2 | 7.4 |
| Hydroxychloroquine+Azithromycin | 24.3 | 0 | 8.6 |
| Plasma | 21.4 | 0 | 8.9 |

**Figure S7.** SUCRA plot for serious adverse events

Cumulative probability plots (Random Effects model):


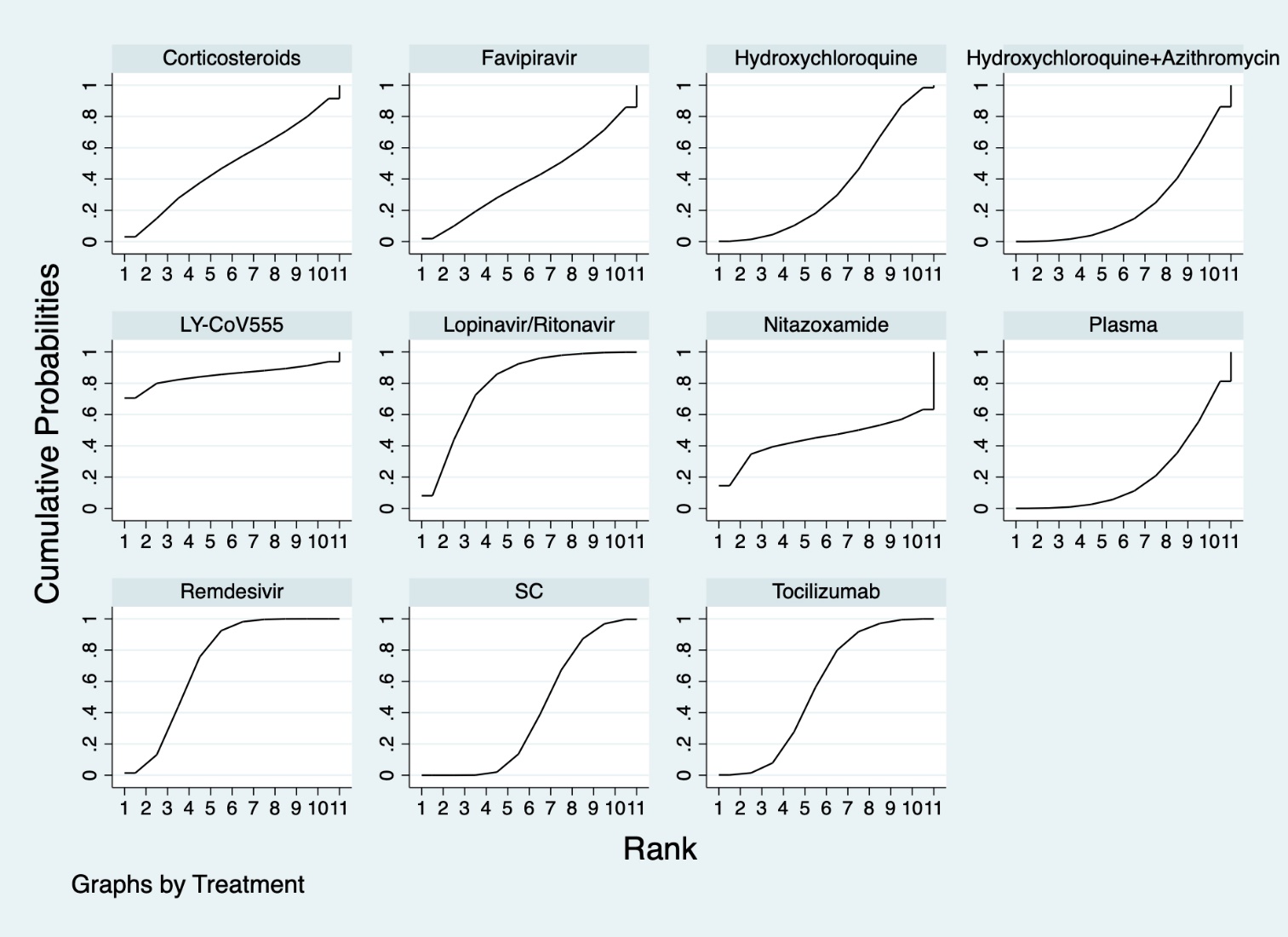


### **Appendix 12. Evaluation of the quality of evidence using GRADE framework for primary outcomes.**

**All-cause mortality**

| **Comparison** | **Number of studies** | **Within-study bias** | **Reporting bias** | **Indirectness** | **Imprecision** | **Heterogeneity** | **Incoherence** | **Confidence rating** |
| --- | --- | --- | --- | --- | --- | --- | --- | --- |
| **Corticosteroids:SC** | 7 | Some concerns | Undetected | No concerns | No concerns | No concerns | No concerns | Moderate |
| **Favipiravir:SC** | 4 | Some concerns | Undetected | No concerns | Major concerns | No concerns | No concerns | Very low |
| **Favipiravir:Umifenovir** | 1 | Major concerns | Undetected | No concerns | Major concerns | No concerns | No concerns | Very low |
| **Hydroxychloroquine:Hydroxychloroquine+Azithromycin** | 3 | Some concerns | Undetected | No concerns | Major concerns | No concerns | No concerns | Very low |
| **Hydroxychloroquine:SC** | 12 | Some concerns | Undetected | No concerns | No concerns | No concerns | No concerns | Moderate |
| **Hydroxychloroquine+Azithromycin:SC** | 2 | Some concerns | Undetected | No concerns | Major concerns | No concerns | No concerns | Very low |
| **Interferon β- 1a:SC** | 2 | Some concerns | Undetected | No concerns | Some concerns | No concerns | No concerns | Low |
| **LY-CoV555:SC** | 1 | No concerns | Undetected | No concerns | Major concerns | No concerns | No concerns | Low |
| **Lopinavir/Ritonavir:SC** | 3 | Some concerns | Undetected | No concerns | No concerns | No concerns | No concerns | Moderate |
| **Plasma:SC** | 7 | Some concerns | Undetected | No concerns | Major concerns | No concerns | No concerns | Very low |
| **Remdesivir:SC** | 4 | Some concerns | Undetected | No concerns | Some concerns | No concerns | No concerns | Low |
| **SC:Tocilizumab** | 5 | No concerns | Undetected | No concerns | Major concerns | No concerns | No concerns | Low |
| **Corticosteroids:Favipiravir** | 0 | Some concerns | Undetected | No concerns | Major concerns | No concerns | No concerns | Very low |
| **Corticosteroids:Hydroxychloroquine** | 0 | Some concerns | Undetected | No concerns | No concerns | No concerns | No concerns | Moderate |
| **Corticosteroids:Hydroxychloroquine+Azithromycin** | 0 | Some concerns | Undetected | No concerns | Some concerns | No concerns | No concerns | Low |
| **Corticosteroids:Interferon β- 1a** | 0 | Some concerns | Undetected | No concerns | No concerns | Some concerns | No concerns | Low |
| **Corticosteroids:LY-CoV555** | 0 | No concerns | Undetected | No concerns | Major concerns | No concerns | No concerns | Low |
| **Corticosteroids:Lopinavir/Ritonavir** | 0 | Some concerns | Undetected | No concerns | Some concerns | No concerns | No concerns | Low |
| **Corticosteroids:Plasma** | 0 | Some concerns | Undetected | No concerns | Major concerns | No concerns | No concerns | Very low |
| **Corticosteroids:Remdesivir** | 0 | Some concerns | Undetected | No concerns | Some concerns | No concerns | No concerns | Low |
| **Corticosteroids:Tocilizumab** | 0 | Some concerns | Undetected | No concerns | Some concerns | No concerns | No concerns | Low |
| **Corticosteroids:Umifenovir** | 0 | Some concerns | Undetected | No concerns | Major concerns | No concerns | No concerns | Very low |
| **Favipiravir:Hydroxychloroquine** | 0 | Some concerns | Undetected | No concerns | Major concerns | No concerns | No concerns | Very low |
| **Favipiravir:Hydroxychloroquine+Azithromycin** | 0 | Some concerns | Undetected | No concerns | Major concerns | No concerns | No concerns | Very low |
| **Favipiravir:Interferon β- 1a** | 0 | Some concerns | Undetected | No concerns | Major concerns | No concerns | No concerns | Very low |
| **Favipiravir:LY-CoV555** | 0 | No concerns | Undetected | No concerns | Major concerns | No concerns | No concerns | Low |
| **Favipiravir:Lopinavir/Ritonavir** | 0 | Some concerns | Undetected | No concerns | Major concerns | No concerns | No concerns | Very low |
| **Favipiravir:Plasma** | 0 | Some concerns | Undetected | No concerns | Major concerns | No concerns | No concerns | Very low |
| **Favipiravir:Remdesivir** | 0 | Some concerns | Undetected | No concerns | Major concerns | No concerns | No concerns | Very low |
| **Favipiravir:Tocilizumab** | 0 | No concerns | Undetected | No concerns | Major concerns | No concerns | No concerns | Low |
| **Hydroxychloroquine:Interferon β- 1a** | 0 | Some concerns | Undetected | No concerns | Major concerns | No concerns | No concerns | Very low |
| **Hydroxychloroquine:LY-CoV555** | 0 | No concerns | Undetected | No concerns | Major concerns | No concerns | No concerns | Low |
| **Hydroxychloroquine:Lopinavir/Ritonavir** | 0 | Some concerns | Undetected | No concerns | Some concerns | No concerns | No concerns | Low |
| **Hydroxychloroquine:Plasma** | 0 | Some concerns | Undetected | No concerns | Some concerns | No concerns | No concerns | Low |
| **Hydroxychloroquine:Remdesivir** | 0 | Some concerns | Undetected | No concerns | Some concerns | No concerns | No concerns | Low |
| **Hydroxychloroquine:Tocilizumab** | 0 | Some concerns | Undetected | No concerns | Major concerns | No concerns | No concerns | Very low |
| **Hydroxychloroquine:Umifenovir** | 0 | Some concerns | Undetected | No concerns | Major concerns | No concerns | No concerns | Very low |
| **Hydroxychloroquine+Azithromycin:Interferon β- 1a** | 0 | Some concerns | Undetected | No concerns | Major concerns | No concerns | No concerns | Very low |
| **Hydroxychloroquine+Azithromycin:LY-CoV555** | 0 | Some concerns | Undetected | No concerns | Major concerns | No concerns | No concerns | Very low |
| **Hydroxychloroquine+Azithromycin:Lopinavir/Ritonavir** | 0 | Some concerns | Undetected | No concerns | Major concerns | No concerns | No concerns | Very low |
| **Hydroxychloroquine+Azithromycin:Plasma** | 0 | Some concerns | Undetected | No concerns | Major concerns | No concerns | No concerns | Very low |
| **Hydroxychloroquine+Azithromycin:Remdesivir** | 0 | Some concerns | Undetected | No concerns | Major concerns | No concerns | No concerns | Very low |
| **Hydroxychloroquine+Azithromycin:Tocilizumab** | 0 | Some concerns | Undetected | No concerns | Major concerns | No concerns | No concerns | Very low |
| **Hydroxychloroquine+Azithromycin:Umifenovir** | 0 | Some concerns | Undetected | No concerns | Major concerns | No concerns | No concerns | Very low |
| **Interferon β- 1a:LY-CoV555** | 0 | No concerns | Undetected | No concerns | Major concerns | No concerns | No concerns | Low |
| **Interferon β- 1a:Lopinavir/Ritonavir** | 0 | Some concerns | Undetected | No concerns | Some concerns | No concerns | No concerns | Low |
| **Interferon β- 1a:Plasma** | 0 | Some concerns | Undetected | No concerns | Major concerns | No concerns | No concerns | Very low |
| **Interferon β- 1a:Remdesivir** | 0 | Some concerns | Undetected | No concerns | Some concerns | No concerns | No concerns | Low |
| **Interferon β- 1a:Tocilizumab** | 0 | Some concerns | Undetected | No concerns | Major concerns | No concerns | No concerns | Very low |
| **Interferon β- 1a:Umifenovir** | 0 | Some concerns | Undetected | No concerns | Major concerns | No concerns | No concerns | Very low |
| **Lopinavir/Ritonavir:LY-CoV555** | 0 | No concerns | Undetected | No concerns | Major concerns | No concerns | No concerns | Low |
| **LY-CoV555:Plasma** | 0 | No concerns | Undetected | No concerns | Major concerns | No concerns | No concerns | Low |
| **LY-CoV555:Remdesivir** | 0 | No concerns | Undetected | No concerns | Major concerns | No concerns | No concerns | Low |
| **LY-CoV555:Tocilizumab** | 0 | No concerns | Undetected | No concerns | Major concerns | No concerns | No concerns | Low |
| **LY-CoV555:Umifenovir** | 0 | Major concerns | Undetected | No concerns | Major concerns | No concerns | No concerns | Very low |
| **Lopinavir/Ritonavir:Plasma** | 0 | Some concerns | Undetected | No concerns | Major concerns | No concerns | No concerns | Very low |
| **Lopinavir/Ritonavir:Remdesivir** | 0 | Some concerns | Undetected | No concerns | Some concerns | No concerns | No concerns | Low |
| **Lopinavir/Ritonavir:Tocilizumab** | 0 | Some concerns | Undetected | No concerns | Major concerns | No concerns | No concerns | Very low |
| **Lopinavir/Ritonavir:Umifenovir** | 0 | Some concerns | Undetected | No concerns | Major concerns | No concerns | No concerns | Very low |
| **Plasma:Remdesivir** | 0 | Some concerns | Undetected | No concerns | Major concerns | No concerns | No concerns | Very low |
| **Plasma:Tocilizumab** | 0 | No concerns | Undetected | No concerns | Major concerns | No concerns | No concerns | Low |
| **Plasma:Umifenovir** | 0 | Major concerns | Undetected | No concerns | Major concerns | No concerns | No concerns | Very low |
| **Remdesivir:Tocilizumab** | 0 | No concerns | Undetected | No concerns | Major concerns | No concerns | No concerns | Low |
| **Remdesivir:Umifenovir** | 0 | Major concerns | Undetected | No concerns | Major concerns | No concerns | No concerns | Very low |
| **SC:Umifenovir** | 0 | Major concerns | Undetected | No concerns | Major concerns | No concerns | No concerns | Very low |
| **Tocilizumab:Umifenovir** | 0 | Major concerns | Undetected | No concerns | Major concerns | No concerns | No concerns | Very low |

**Adverse events**

| **Comparison** | **Number of studies** | **Within-study bias** | **Reporting bias** | **Indirectness** | **Imprecision** | **Heterogeneity** | **Incoherence** | **Confidence rating** |
| --- | --- | --- | --- | --- | --- | --- | --- | --- |
| **Corticosteroids:SC** | 2 | Some concerns | Undetected | No concerns | Major concerns | No concerns | No concerns | Very low |
| **Favipiravir:SC** | 3 | Some concerns | Undetected | No concerns | No concerns | Some concerns | No concerns | Low |
| **Favipiravir:Tocilizumab** | 1 | Some concerns | Undetected | No concerns | Some concerns | Some concerns | No concerns | Very low |
| **Favipiravir:Umifenovir** | 1 | Major concerns | Undetected | No concerns | Some concerns | Some concerns | No concerns | Very low |
| **Hydroxychloroquine:Hydroxychloroquine+Azithromycin** | 2 | Some concerns | Undetected | No concerns | Major concerns | No concerns | No concerns | Very low |
| **Hydroxychloroquine:SC** | 8 | Some concerns | Undetected | No concerns | Some concerns | Some concerns | No concerns | Very low |
| **Hydroxychloroquine+Azithromycin:SC** | 2 | Some concerns | Undetected | No concerns | Some concerns | Some concerns | No concerns | Very low |
| **LY-CoV555:SC** | 1 | No concerns | Undetected | No concerns | Major concerns | No concerns | No concerns | Low |
| **Lopinavir/Ritonavir:SC** | 2 | Some concerns | Undetected | No concerns | Major concerns | No concerns | No concerns | Very low |
| **Lopinavir/Ritonavir:Umifenovir** | 1 | No concerns | Undetected | No concerns | Major concerns | No concerns | No concerns | Low |
| **Nitazoxamide:SC** | 1 | Major concerns | Undetected | No concerns | Major concerns | No concerns | No concerns | Very low |
| **Plasma:SC** | 4 | No concerns | Undetected | No concerns | Major concerns | No concerns | No concerns | Low |
| **Remdesivir:SC** | 3 | No concerns | Undetected | No concerns | Major concerns | No concerns | No concerns | Low |
| **SC:Tocilizumab** | 4 | No concerns | Undetected | No concerns | Major concerns | No concerns | No concerns | Low |
| **SC:Umifenovir** | 1 | Some concerns | Undetected | No concerns | Major concerns | No concerns | No concerns | Very low |
| **Corticosteroids:Favipiravir** | 0 | Some concerns | Undetected | No concerns | Some concerns | No concerns | No concerns | Low |
| **Corticosteroids:Hydroxychloroquine** | 0 | Some concerns | Undetected | No concerns | Some concerns | Some concerns | No concerns | Very low |
| **Corticosteroids:Hydroxychloroquine+Azithromycin** | 0 | Some concerns | Undetected | No concerns | Some concerns | Some concerns | No concerns | Very low |
| **Corticosteroids:LY-CoV555** | 0 | No concerns | Undetected | No concerns | Major concerns | No concerns | No concerns | Low |
| **Corticosteroids:Lopinavir/Ritonavir** | 0 | Some concerns | Undetected | No concerns | Major concerns | No concerns | No concerns | Very low |
| **Corticosteroids:Nitazoxamide** | 0 | Major concerns | Undetected | No concerns | Major concerns | No concerns | No concerns | Very low |
| **Corticosteroids:Plasma** | 0 | No concerns | Undetected | No concerns | Major concerns | No concerns | No concerns | Low |
| **Corticosteroids:Remdesivir** | 0 | Some concerns | Undetected | No concerns | Major concerns | No concerns | No concerns | Very low |
| **Corticosteroids:Tocilizumab** | 0 | Some concerns | Undetected | No concerns | Major concerns | No concerns | No concerns | Very low |
| **Corticosteroids:Umifenovir** | 0 | Some concerns | Undetected | No concerns | Major concerns | No concerns | No concerns | Very low |
| **Favipiravir:Hydroxychloroquine** | 0 | Some concerns | Undetected | No concerns | Major concerns | No concerns | No concerns | Very low |
| **Favipiravir:Hydroxychloroquine+Azithromycin** | 0 | Some concerns | Undetected | No concerns | Major concerns | No concerns | No concerns | Very low |
| **Favipiravir:LY-CoV555** | 0 | No concerns | Undetected | No concerns | Some concerns | Some concerns | No concerns | Low |
| **Favipiravir:Lopinavir/Ritonavir** | 0 | Some concerns | Undetected | No concerns | Major concerns | No concerns | No concerns | Very low |
| **Favipiravir:Nitazoxamide** | 0 | Major concerns | Undetected | No concerns | Major concerns | No concerns | No concerns | Very low |
| **Favipiravir:Plasma** | 0 | No concerns | Undetected | No concerns | Major concerns | No concerns | No concerns | Low |
| **Favipiravir:Remdesivir** | 0 | Some concerns | Undetected | No concerns | Some concerns | Some concerns | No concerns | Very low |
| **Hydroxychloroquine:LY-CoV555** | 0 | No concerns | Undetected | No concerns | Major concerns | No concerns | No concerns | Low |
| **Hydroxychloroquine:Lopinavir/Ritonavir** | 0 | Some concerns | Undetected | No concerns | Major concerns | No concerns | No concerns | Very low |
| **Hydroxychloroquine:Nitazoxamide** | 0 | Major concerns | Undetected | No concerns | Major concerns | No concerns | No concerns | Very low |
| **Hydroxychloroquine:Plasma** | 0 | No concerns | Undetected | No concerns | Major concerns | No concerns | No concerns | Low |
| **Hydroxychloroquine:Remdesivir** | 0 | Some concerns | Undetected | No concerns | Some concerns | Some concerns | No concerns | Very low |
| **Hydroxychloroquine:Tocilizumab** | 0 | No concerns | Undetected | No concerns | Some concerns | Some concerns | No concerns | Low |
| **Hydroxychloroquine:Umifenovir** | 0 | Some concerns | Undetected | No concerns | Major concerns | No concerns | No concerns | Very low |
| **Hydroxychloroquine+Azithromycin:LY-CoV555** | 0 | No concerns | Undetected | No concerns | Major concerns | No concerns | No concerns | Low |
| **Hydroxychloroquine+Azithromycin:Lopinavir/Ritonavir** | 0 | Some concerns | Undetected | No concerns | Major concerns | No concerns | No concerns | Very low |
| **Hydroxychloroquine+Azithromycin:Nitazoxamide** | 0 | Some concerns | Undetected | No concerns | Major concerns | No concerns | No concerns | Very low |
| **Hydroxychloroquine+Azithromycin:Plasma** | 0 | Some concerns | Undetected | No concerns | Major concerns | No concerns | No concerns | Very low |
| **Hydroxychloroquine+Azithromycin:Remdesivir** | 0 | Some concerns | Undetected | No concerns | Some concerns | Some concerns | No concerns | Very low |
| **Hydroxychloroquine+Azithromycin:Tocilizumab** | 0 | Some concerns | Undetected | No concerns | Major concerns | No concerns | No concerns | Very low |
| **Hydroxychloroquine+Azithromycin:Umifenovir** | 0 | Some concerns | Undetected | No concerns | Major concerns | No concerns | No concerns | Very low |
| **Lopinavir/Ritonavir:LY-CoV555** | 0 | No concerns | Undetected | No concerns | Major concerns | No concerns | No concerns | Low |
| **LY-CoV555:Nitazoxamide** | 0 | No concerns | Undetected | No concerns | Major concerns | No concerns | No concerns | Low |
| **LY-CoV555:Plasma** | 0 | No concerns | Undetected | No concerns | Major concerns | No concerns | No concerns | Low |
| **LY-CoV555:Remdesivir** | 0 | No concerns | Undetected | No concerns | Major concerns | No concerns | No concerns | Low |
| **LY-CoV555:Tocilizumab** | 0 | No concerns | Undetected | No concerns | Major concerns | No concerns | No concerns | Low |
| **LY-CoV555:Umifenovir** | 0 | No concerns | Undetected | No concerns | Major concerns | No concerns | No concerns | Low |
| **Lopinavir/Ritonavir:Nitazoxamide** | 0 | Major concerns | Undetected | No concerns | Major concerns | No concerns | No concerns | Very low |
| **Lopinavir/Ritonavir:Plasma** | 0 | Some concerns | Undetected | No concerns | Major concerns | No concerns | No concerns | Very low |
| **Lopinavir/Ritonavir:Remdesivir** | 0 | Some concerns | Undetected | No concerns | Major concerns | No concerns | No concerns | Very low |
| **Lopinavir/Ritonavir:Tocilizumab** | 0 | Some concerns | Undetected | No concerns | Major concerns | No concerns | No concerns | Very low |
| **Nitazoxamide:Plasma** | 0 | Major concerns | Undetected | No concerns | Major concerns | No concerns | No concerns | Very low |
| **Nitazoxamide:Remdesivir** | 0 | Major concerns | Undetected | No concerns | Major concerns | No concerns | No concerns | Very low |
| **Nitazoxamide:Tocilizumab** | 0 | Major concerns | Undetected | No concerns | Major concerns | No concerns | No concerns | Very low |
| **Nitazoxamide:Umifenovir** | 0 | Major concerns | Undetected | No concerns | Major concerns | No concerns | No concerns | Very low |
| **Plasma:Remdesivir** | 0 | No concerns | Undetected | No concerns | Major concerns | No concerns | No concerns | Low |
| **Plasma:Tocilizumab** | 0 | No concerns | Undetected | No concerns | Major concerns | No concerns | No concerns | Low |
| **Plasma:Umifenovir** | 0 | No concerns | Undetected | No concerns | Major concerns | No concerns | No concerns | Low |
| **Remdesivir:Tocilizumab** | 0 | No concerns | Undetected | No concerns | Major concerns | No concerns | No concerns | Low |
| **Remdesivir:Umifenovir** | 0 | Some concerns | Undetected | No concerns | Major concerns | No concerns | No concerns | Very low |
| **Tocilizumab:Umifenovir** | 0 | No concerns | Undetected | No concerns | Major concerns | No concerns | No concerns | Low |

**Serious adverse events**

| **Comparison** | **Number of studies** | **Within-study bias** | **Reporting bias** | **Indirectness** | **Imprecision** | **Heterogeneity** | **Incoherence** | **Confidence rating** |
| --- | --- | --- | --- | --- | --- | --- | --- | --- |
| **Corticosteroids:SC** | 3 | Some concerns | Undetected | No concerns | Major concerns | No concerns | No concerns | Very low |
| **Favipiravir:SC** | 3 | Major concerns | Undetected | No concerns | Major concerns | No concerns | No concerns | Very low |
| **Favipiravir:Tocilizumab** | 1 | Major concerns | Undetected | No concerns | Major concerns | No concerns | No concerns | Very low |
| **Hydroxychloroquine:Hydroxychloroquine+Azithromycin** | 3 | Some concerns | Undetected | No concerns | Some concerns | No concerns | No concerns | Very low |
| **Hydroxychloroquine:SC** | 7 | No concerns | Undetected | No concerns | Major concerns | No concerns | No concerns | Low |
| **Hydroxychloroquine+Azithromycin:SC** | 2 | Some concerns | Undetected | No concerns | Major concerns | No concerns | No concerns | Very low |
| **LY-CoV555:SC** | 1 | No concerns | Undetected | No concerns | Major concerns | No concerns | No concerns | Low |
| **Lopinavir/Ritonavir:SC** | 2 | Some concerns | Undetected | No concerns | Some concerns | No concerns | No concerns | Low |
| **Nitazoxamide:SC** | 1 | Major concerns | Undetected | No concerns | Major concerns | No concerns | No concerns | Very low |
| **Plasma:SC** | 2 | No concerns | Undetected | No concerns | Major concerns | No concerns | No concerns | Low |
| **Remdesivir:SC** | 3 | No concerns | Undetected | No concerns | No concerns | No concerns | No concerns | High |
| **SC:Tocilizumab** | 6 | No concerns | Undetected | No concerns | Some concerns | No concerns | No concerns | Moderate |
| **Corticosteroids:Favipiravir** | 0 | Some concerns | Undetected | No concerns | Major concerns | No concerns | No concerns | Very low |
| **Corticosteroids:Hydroxychloroquine** | 0 | Some concerns | Undetected | No concerns | Major concerns | No concerns | No concerns | Very low |
| **Corticosteroids:Hydroxychloroquine+Azithromycin** | 0 | Some concerns | Undetected | No concerns | Major concerns | No concerns | No concerns | Very low |
| **Corticosteroids:LY-CoV555** | 0 | No concerns | Undetected | No concerns | Major concerns | No concerns | No concerns | Low |
| **Corticosteroids:Lopinavir/Ritonavir** | 0 | Some concerns | Undetected | No concerns | Major concerns | No concerns | No concerns | Very low |
| **Corticosteroids:Nitazoxamide** | 0 | Major concerns | Undetected | No concerns | Major concerns | No concerns | No concerns | Very low |
| **Corticosteroids:Plasma** | 0 | No concerns | Undetected | No concerns | Major concerns | No concerns | No concerns | Low |
| **Corticosteroids:Remdesivir** | 0 | No concerns | Undetected | No concerns | Major concerns | No concerns | No concerns | Low |
| **Corticosteroids:Tocilizumab** | 0 | Some concerns | Undetected | No concerns | Major concerns | No concerns | No concerns | Very low |
| **Favipiravir:Hydroxychloroquine** | 0 | Major concerns | Undetected | No concerns | Major concerns | No concerns | No concerns | Very low |
| **Favipiravir:Hydroxychloroquine+Azithromycin** | 0 | Some concerns | Undetected | No concerns | Major concerns | No concerns | No concerns | Very low |
| **Favipiravir:LY-CoV555** | 0 | No concerns | Undetected | No concerns | Major concerns | No concerns | No concerns | Low |
| **Favipiravir:Lopinavir/Ritonavir** | 0 | Some concerns | Undetected | No concerns | Major concerns | No concerns | No concerns | Very low |
| **Favipiravir:Nitazoxamide** | 0 | Major concerns | Undetected | No concerns | Major concerns | No concerns | No concerns | Very low |
| **Favipiravir:Plasma** | 0 | No concerns | Undetected | No concerns | Major concerns | No concerns | No concerns | Low |
| **Favipiravir:Remdesivir** | 0 | No concerns | Undetected | No concerns | Major concerns | No concerns | No concerns | Low |
| **Hydroxychloroquine:LY-CoV555** | 0 | No concerns | Undetected | No concerns | Major concerns | No concerns | No concerns | Low |
| **Hydroxychloroquine:Lopinavir/Ritonavir** | 0 | Some concerns | Undetected | No concerns | Some concerns | No concerns | No concerns | Low |
| **Hydroxychloroquine:Nitazoxamide** | 0 | Major concerns | Undetected | No concerns | Major concerns | No concerns | No concerns | Very low |
| **Hydroxychloroquine:Plasma** | 0 | No concerns | Undetected | No concerns | Major concerns | No concerns | No concerns | Low |
| **Hydroxychloroquine:Remdesivir** | 0 | No concerns | Undetected | No concerns | Some concerns | No concerns | No concerns | Moderate |
| **Hydroxychloroquine:Tocilizumab** | 0 | No concerns | Undetected | No concerns | Major concerns | No concerns | No concerns | Low |
| **Hydroxychloroquine+Azithromycin:LY-CoV555** | 0 | No concerns | Undetected | No concerns | Major concerns | No concerns | No concerns | Low |
| **Hydroxychloroquine+Azithromycin:Lopinavir/Ritonavir** | 0 | Some concerns | Undetected | No concerns | Some concerns | No concerns | No concerns | Low |
| **Hydroxychloroquine+Azithromycin:Nitazoxamide** | 0 | Some concerns | Undetected | No concerns | Major concerns | No concerns | No concerns | Very low |
| **Hydroxychloroquine+Azithromycin:Plasma** | 0 | No concerns | Undetected | No concerns | Major concerns | No concerns | No concerns | Low |
| **Hydroxychloroquine+Azithromycin:Remdesivir** | 0 | No concerns | Undetected | No concerns | Some concerns | No concerns | No concerns | Moderate |
| **Hydroxychloroquine+Azithromycin:Tocilizumab** | 0 | Some concerns | Undetected | No concerns | Major concerns | No concerns | No concerns | Very low |
| **Lopinavir/Ritonavir:LY-CoV555** | 0 | No concerns | Undetected | No concerns | Major concerns | No concerns | No concerns | Low |
| **LY-CoV555:Nitazoxamide** | 0 | No concerns | Undetected | No concerns | Major concerns | No concerns | No concerns | Low |
| **LY-CoV555:Plasma** | 0 | No concerns | Undetected | No concerns | Major concerns | No concerns | No concerns | Low |
| **LY-CoV555:Remdesivir** | 0 | No concerns | Undetected | No concerns | Major concerns | No concerns | No concerns | Low |
| **LY-CoV555:Tocilizumab** | 0 | No concerns | Undetected | No concerns | Major concerns | No concerns | No concerns | Low |
| **Lopinavir/Ritonavir:Nitazoxamide** | 0 | Major concerns | Undetected | No concerns | Major concerns | No concerns | No concerns | Very low |
| **Lopinavir/Ritonavir:Plasma** | 0 | No concerns | Undetected | No concerns | No concerns | No concerns | No concerns | High |
| **Lopinavir/Ritonavir:Remdesivir** | 0 | Some concerns | Undetected | No concerns | Major concerns | No concerns | No concerns | Very low |
| **Lopinavir/Ritonavir:Tocilizumab** | 0 | Some concerns | Undetected | No concerns | Major concerns | No concerns | No concerns | Very low |
| **Nitazoxamide:Plasma** | 0 | Major concerns | Undetected | No concerns | Major concerns | No concerns | No concerns | Very low |
| **Nitazoxamide:Remdesivir** | 0 | Major concerns | Undetected | No concerns | Major concerns | No concerns | No concerns | Very low |
| **Nitazoxamide:Tocilizumab** | 0 | Major concerns | Undetected | No concerns | Major concerns | No concerns | No concerns | Very low |
| **Plasma:Remdesivir** | 0 | No concerns | Undetected | No concerns | No concerns | No concerns | No concerns | High |
| **Plasma:Tocilizumab** | 0 | No concerns | Undetected | No concerns | Some concerns | No concerns | No concerns | Moderate |
| **Remdesivir:Tocilizumab** | 0 | No concerns | Undetected | No concerns | Some concerns | No concerns | No concerns | Moderate |
